# Associations of Coexisting Metabolic Dysfunction‐Associated Steatotic Liver Disease and Sarcopenia‐Related Abnormalities With Adverse Clinical Outcomes Across Obesity States

**DOI:** 10.1002/mco2.71111

**Published:** 2026-10-29

**Authors:** Hong Fan, Xianhua Mao, Taotao Yan, Scott Barnett, Jing Zhang, Ramsey Cheung, Xuehong Zhang, Mindie H. Nguyen

**Affiliations:** ^1^ Division of Gastroenterology and Hepatology Stanford University Medical Center Palo Alto California USA; ^2^ The Third Unit Department of Hepatology Beijing Youan Hospital Capital Medical University Beijing China; ^3^ Division of Gastroenterology and Hepatology Veterans Affairs Palo Alto Healthcare System Palo Alto California USA; ^4^ Department of Nutrition Harvard T.H. Chan School of Public Health Boston Massachusetts USA; ^5^ Yale University School of Nursing Orange Connecticut USA; ^6^ Department of Epidemiology Yale School of Public Health New Haven Connecticut USA; ^7^ Department of Epidemiology and Population Health Stanford University Medical Center Palo Alto California USA

**Keywords:** cardiovascular disease, hepatocellular carcinoma, metabolic dysfunction‐associated steatotic liver disease, muscle health, sarcopenia

## Abstract

Metabolic dysfunction‐associated steatotic liver disease (MASLD) is associated with adverse liver and non‐liver outcomes, but the impact of coexisting sarcopenia‐related abnormalities (e.g., low muscle strength [MS], muscle mass, and physical performance) remains unclear. In a prospective UK Biobank cohort (*n* = 438,748), we evaluated their joint associations with incidence and risks of all‐cause mortality, cirrhosis, hepatocellular carcinoma (HCC), cardiovascular disease (CVD), chronic kidney disease (CKD), non‐liver cancers, and obesity‐related cancers across MASLD and sarcopenia‐related abnormality status. Over a median follow‐up of 13.8 years, 401 HCC, 1714 cirrhosis, 39,534 CVD, 19,637 CKD, 38,240 non‐liver cancers, 12,536 obesity‐related cancers, and 34,801 deaths occurred. A stepwise increase in both incidence and adjusted risk of liver and non‐liver outcomes was observed across MASLD and sarcopenia strata. Within the coexistence group, the obesity (vs. non‐obesity) subgroup generally had higher risks. For example, for cirrhosis, adjusted hazard ratios increased from 3.93 to 7.36 then 8.48 with MASLD alone, coexisting MASLD with low MS overall and coexisting MASLD, low MS and obesity, respectively. Similar findings were observed in external validation analyses using the 2011–2018 US National Health and Nutrition Examination Survey cohort. Together, these observations inform risk stratification strategy and management of MASLD to include sarcopenia assessment.

## Graphical abstract

Using a population‐based cohort, we evaluated the joint effects of MASLD, obesity, and sarcopenia‐related abnormalities on clinical outcomes.

We found that obesity acts as a key amplifier of risk, particularly when coexisting with MASLD and sarcopenia, leading to up to an eightfold higher risk of death and multi‐system adverse outcomes.

A clear stepwise gradient was observed, with the highest risk among individuals with MASLD, obesity, and sarcopenia‐related abnormalities.

These findings highlight the central role of obesity in driving risk escalation and support integrated risk stratification across metabolic and muscle health.

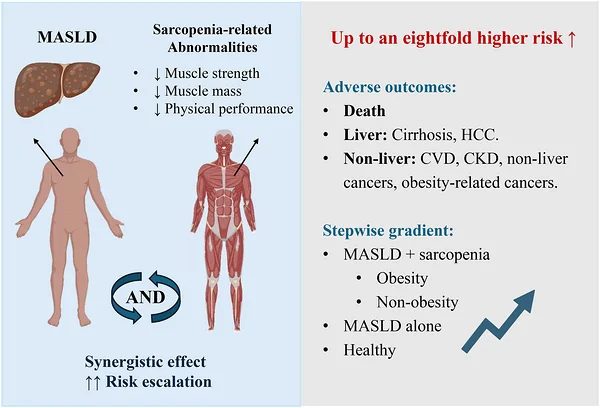

## 1 Introduction

Metabolic dysfunction‐associated steatotic liver disease (MASLD) has emerged as the most common chronic liver disease globally, with its prevalence rising substantially over the past decade partly driven by the epidemic of obesity and metabolic syndrome [1, 2]. Although MASLD is often asymptomatic in its early stages, it can progress to advanced fibrosis, cirrhosis, liver failure, and hepatocellular carcinoma (HCC) [3, 4, 5]. MASLD is also associated with an increased risk of extrahepatic complications, including cardiovascular disease (CVD), chronic kidney disease (CKD), and non‐liver cancers [6, 7, 8]. Identifying factors that contribute to these adverse outcomes is therefore important for improving risk stratification and management of individuals with MASLD.

Sarcopenia, characterized by a progressive loss of skeletal muscle mass and function, is increasingly recognized as a critical determinant of adverse health outcomes, particularly in aging and metabolically compromised populations [9, 10]. MASLD and sarcopenia share several pathophysiological mechanisms, including insulin resistance, systemic inflammation, and mitochondrial dysfunction, which may jointly accelerate disease progression [11]. Although previous epidemiological studies have examined the independent associations of MASLD and sarcopenia with clinical outcomes [12, 13, 14], the long‐term clinical impact of their coexistence remains incompletely understood.

Sarcopenia is currently defined by a stepwise approach incorporating three key components: muscle strength (MS), muscle mass (MM), and physical performance (PP). According to international consensus [15], low MS indicates probable sarcopenia. The additional presence of low MM confirms sarcopenia, while the further presence of low PP indicates severe sarcopenia. Of note, a substantial proportion of individuals with MASLD have obesity, and individuals with obesity typically have greater absolute MS and MM but poorer PP than those without obesity [16, 17, 18, 19]. Therefore, evaluating muscle health in individuals with MASLD without considering obesity status may result in incomplete or potentially misleading risk classification. However, obesity status has not been adequately considered in existing studies evaluating different dimensions of muscle health in MASLD. Furthermore, prospective evidence regarding the associations of these muscle health dimensions with a broad range of liver and non‐liver outcomes remains limited.

In this large‐scale prospective cohort study using data from the UK Biobank, we examined the joint associations of MASLD and abnormalities in MS, MM, and PP with all‐cause mortality and a broad range of liver and non‐liver outcomes across obesity states. As a secondary objective, we performed subgroup analyses according to age, sex, diabetes status, and estimated liver fibrosis severity, as assessed using the Fibrosis‐4 index, to identify populations at particularly high risk. We also evaluated the generalizability of the principal associations in an independent US cohort. By comprehensively assessing multiple dimensions of muscle health while accounting for obesity status, this study aimed to improve risk stratification and inform the integration of muscle health assessment into MASLD management.

## 2 Results

### 2.1 Characteristics of the Study Population

Baseline characteristics of the study population are summarized in Table 1 and Figure 1A–E. Among the 438,748 participants included in the analysis, 274,430 were healthy without MASLD or sarcopenia‐related abnormalities, whereas 164,318 had MASLD, including 92,599 (56.4%) with obesity (MASLD‐O) and 71,719 (43.6%) without obesity (MASLD‐NO). Compared with healthy participants, those with MASLD were slightly older (mean age 57.2 vs. 55.7 years), more frequently male (62.2% vs. 36.6%), especially in the non‐obese subgroup (76.9%), and had markedly higher BMI (31.4 vs. 25.2 kg/m^2^), especially in the obese subgroup (34.2 kg/m^2^). Socioeconomic differences were evident, with participants with MASLD being more likely to live in more deprived areas and have lower educational attainment. Comorbidities were also more prevalent, including diabetes (17.0% vs. 3.6%), hypertension (45.1% vs. 22.6%), and hyperlipidemia (4.4% vs. 1.8%). Biochemical and metabolic profiles indicated more adverse features in participants with MASLD than healthy participants, with higher liver enzymes (ALT, AST, GGT), elevated CRP, triglycerides, and HbA1c, as well as lower HDL‐C. The prevalence of advanced fibrosis, as defined by FIB‐4 >2.67, was slightly higher in MASLD (2.3%) compared with healthy participants (2.0%). Probable sarcopenia defined by low grip strength was present in 5.8% of participants with MASLD (6.5% in MASLD‐O and 4.9% in MASLD‐NO), while confirmed and severe sarcopenia were rare. Figure 1A–E further illustrates that participants with MASLD were more frequently classified into the highest tertile categories of grip strength and skeletal muscle index compared with healthy participants, and those reporting low physical performance were predominantly in the obese subgroup.

**TABLE 1:** Baseline characteristics of study population according to MASLD status.

		MASLD		
	Healthy (*n* = 274,430)	Overall (*n* = 164,318)	MASLD‐NO (*n* = 71,719)	MASLD‐O (*n* = 92,599)
Age (year)	55.66 (8.21)	57.18 (7.84)	57.43 (7.85)	56.98 (7.82)
Male	100,326 (36.6)	102,134 (62.2)	55,163 (76.9)	46,971 (50.7)
White ethnicity	260,335 (94.9)	154,545 (94.1)	67,702 (94.4)	86,843 (93.8)
Townsend deprivation				
Q1	71,998 (26.3)	37,346 (22.8)	18,197 (25.4)	19,149 (20.7)
Q2‐Q3	139,259 (50.8)	79,820 (48.7)	35,674 (49.8)	44,146 (47.8)
Q4	62,694 (22.9)	46,859 (28.6)	17,726 (24.8)	29,133 (31.5)
Education levels				
High	114,047 (42.0)	52,913 (32.2)	25,512 (35.6)	27,401 (29.6)
Low	119,402 (44.0)	74,564 (45.4)	31,685 (44.2)	42,879 (46.3)
Others	38,000 (14.0)	36,716 (22.4)	14,444 (20.2)	22,272 (24.1)
BMI (kg/m^2^)	25.17 (3.08)	31.40 (4.50)	27.74 (1.60)	34.24 (3.94)
Diabetes	9919 (3.6)	27,867 (17.0)	8164 (11.4)	19,703 (21.3)
Hyperlipidemia	4911 (1.8)	7291 (4.4)	2914 (4.1)	4377 (4.7)
Hypertension	61,933 (22.6)	74,110 (45.1)	27,694 (38.6)	46,416 (50.1)
FIB‐4	1.34 (1.38)	1.32 (2.69)	1.34 (1.03)	1.30 (3.46)
FIB‐4 >2.67	4741 (2.0)	3661 (2.3)	1687 (2.4)	1974 (2.2)
Probable sarcopenia	0 (0.0)	9499 (5.8)	3494 (4.9)	6005 (6.5)
Sarcopenia	0 (0.0)	98 (0.1)	82 (0.1)	16 (0.0)
Severe sarcopenia	0 (0.0)	46 (0.0)	38 (0.1)	8 (0.0)
CCI	1.02 (2.09)	1.74 (2.67)	1.56 (2.55)	1.88 (2.75)
Age‐adjusted CCI	2.14 (2.39)	3.01 (2.96)	2.85 (2.84)	3.13 (3.04)
ALT (U/L)	19.45 (9.70)	29.86 (17.15)	30.37 (17.28)	29.47 (17.03)
AST (U/L)	24.57 (8.20)	28.57 (12.66)	29.10 (13.17)	28.17 (12.24)
Albumin (g/L)	45.32 (2.58)	45.11 (2.64)	45.62 (2.58)	44.71 (2.62)
ALP (U/L)	79.54 (23.48)	88.78 (28.49)	88.21 (31.18)	89.22 (26.21)
CRP (mg/L)	1.83 (3.59)	3.60 (4.82)	2.79 (4.26)	4.23 (5.13)
Cholesterol (mmol/L)	5.68 (1.08)	5.71 (1.22)	5.87 (1.22)	5.59 (1.21)
GGT (U/L)	25.72 (18.79)	53.52 (55.99)	59.71 (63.98)	48.73 (48.39)
Glucose (mmol/L)	4.97 (0.89)	5.34 (1.60)	5.20 (1.38)	5.45 (1.74)
HbA1c (mmol/mol)	34.89 (5.05)	37.95 (8.40)	36.76 (7.23)	38.87 (9.10)
HDL‐C (mmol/L)	1.57 (0.38)	1.25 (0.29)	1.25 (0.29)	1.25 (0.29)
LDL‐C (mmol/L)	3.51 (0.82)	3.64 (0.92)	3.74 (0.91)	3.55 (0.91)
Triglycerides (mmol/L)	1.33 (0.61)	2.39 (1.20)	2.56 (1.21)	2.25 (1.17)
PLT (10^9^ cells/L)	252.75 (58.56)	253.49 (61.15)	249.72 (60.03)	256.43 (61.85)

Abbreviations: ALT, alanine aminotransferase; AST, aspartate aminotransferase; ALP, alkaline phosphatase; BMI, body mass index; CCI, Charlson Comorbidity Index; CRP, C‐reactive protein; FIB‐4, Fibrosis‐4 Index; GGT, gamma‐glutamyl transferase; HbA1c, glycated hemoglobin; HDL‐C, high‐density lipoprotein cholesterol; LDL‐C, low‐density lipoprotein cholesterol; MASLD, Metabolic dysfunction‐associated steatotic liver disease; NO, non‐obesity; O, obesity; PLT, platelet count; Q1–Q4, quartiles 1–4.

**FIGURE 1:**
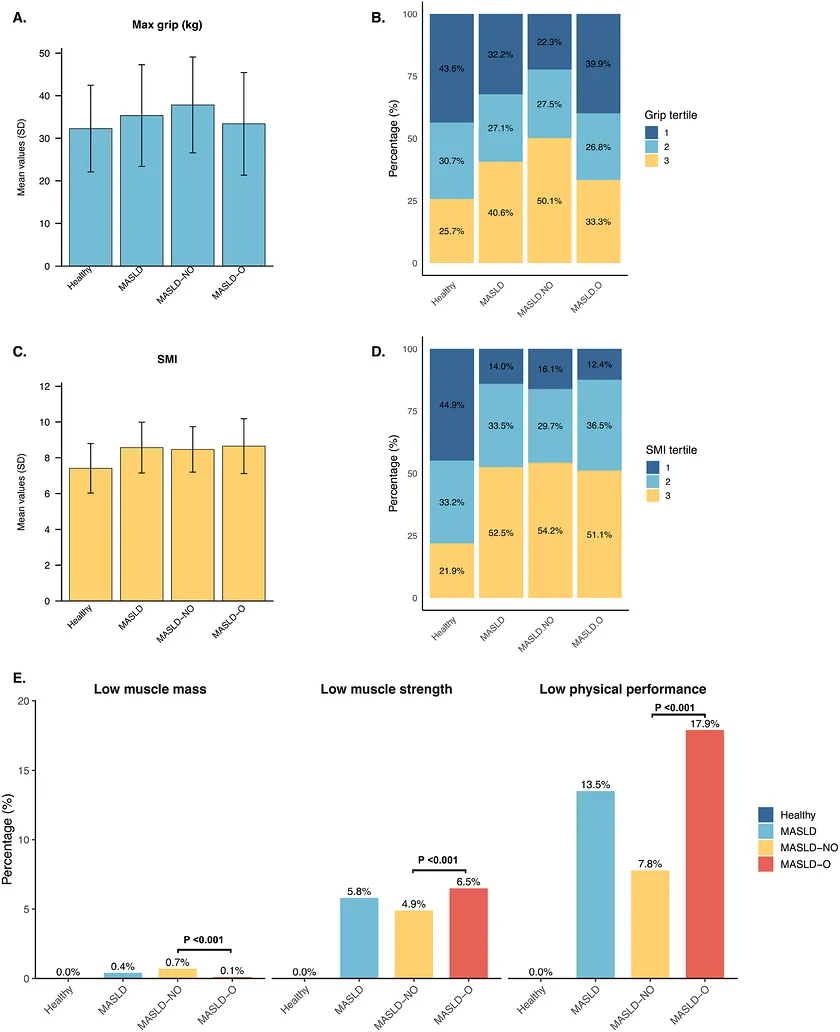
Baseline distribution of muscle strength, muscle mass, and physical performance among healthy individuals and MASLD subgroups. (A) Mean and standard deviation of max handgrip strength; (B) distribution of handgrip strength tertiles; (C) mean and standard deviation of skeletal muscle index (SMI); (D) distribution of SMI tertiles; and (E) prevalence of sarcopenia‐related abnormalities (low muscle strength, low muscle mass, or low physical performance) across healthy individuals, MASLD overall, MASLD without obesity (MASLD‐NO), and MASLD with obesity (MASLD‐O). Max handgrip strength was used to estimate muscle strength, and SMI was used to estimate muscle mass.

Further stratified analyses (Table S2) demonstrated that participants with MASLD with low MS, low MM, or poor PP were generally older, more likely male, and exhibited more adverse metabolic and inflammatory profiles compared with those with normal measures. Among these, obese subgroups consistently showed the most unfavorable cardiometabolic indices.

Diabetes, hypertension, and hyperlipidemia were defined using ICD‐9 and ICD‐10 diagnostic codes. The CCI was calculated using the *comorbidity* package in R based on ICD‐10 diagnostic codes. The age‐adjusted CCI was derived by adding one point for each decade of age above 50 years, following the original Charlson methodology. Continuous variables are presented as mean (SD) and categorical variables as absolute (*n*) and relative frequencies (%).

### 2.2 Clinical Outcomes Incidence

During follow‐up, a total of 401 cases of HCC, 1714 cases of cirrhosis, 39,534 cases of CVD, 19,637 cases of CKD, 38,240 cases of non‐liver cancers, 12,536 cases of obesity‐related cancers, and 34,801 deaths were recorded. The corresponding median follow‐up times were 13.8, 13.8, 13.7, 13.8, 13.7, 13.8, and 13.7 years, respectively. Across all outcomes, incidence rates were lowest in healthy participants, higher in MASLD without sarcopenia‐related abnormalities, and highest in MASLD with sarcopenia‐related abnormalities, particularly in the obese subgroup (Table 2).

**TABLE 2:** Incidence of clinical outcomes according to categories of MASLD and muscle strength (MS), muscle mass (MM), and physical performance (PP).

		MS		MM		PP	
Outcomes	Groups	Incident cases	Incidence (10,000 PYs)	Incident cases	Incidence (10,000 PYs)	Incident cases	Incidence (10,000 PYs)
Death	Healthy	16,765	45.38 (44.69, 46.07)	16,765	45.38 (44.69, 46.07)	16,765	45.38 (44.69, 46.07)
	MASLD alone	16,292	78.94 (77.73, 80.16)	17,927	82.37 (81.17, 83.58)	13,312	69.93 (68.75, 71.13)
	MASLD + SRA	1744	144.17 (137.48, 151.09)	109	129.95 (106.70, 156.76)	4724	167.92 (163.17, 172.78)
	Non‐obesity	632	142.17 (131.30, 153.70)	87	127.03 (101.74, 156.69)	1355	193.39 (183.23, 203.97)
	Obesity	1112	145.32 (136.91, 154.12)	22	142.95 (89.59, 216.43)	3369	159.48 (154.14, 164.95)
Cirrhosis	Healthy	460	1.22 (1.11, 1.33)	460	1.22 (1.11, 1.33)	460	1.22 (1.11, 1.33)
	MASLD alone	1109	5.18 (4.88, 5.50)	1249	5.53 (5.23, 5.84)	900	4.58 (4.29, 4.89)
	MASLD + SRA	145	11.26 (9.50, 13.25)	5	5.60 (1.82, 13.07)	354	11.66 (10.48, 12.94)
	Non‐obesity	42	8.86 (6.38, 11.97)	4	5.49 (1.49, 14.04)	81	10.51 (8.35, 13.07)
	Obesity	103	12.66 (10.33, 15.35)	1	6.12 (0.16, 34.12)	273	12.05 (10.66, 13.57)
HCC	Healthy	115	0.30 (0.25, 0.37)	115	0.30 (0.25, 0.37)	115	0.30 (0.25, 0.37)
	MASLD alone	252	1.17 (1.03, 1.33)	284	1.25 (1.11, 1.41)	209	1.06 (0.92, 1.22)
	MASLD + SRA	34	2.62 (1.81, 3.66)	2	2.23 (0.27, 8.06)	77	2.51 (1.98, 3.14)
	Non‐obesity	13	2.72 (1.45, 4.65)	2	2.74 (0.33, 9.89)	25	3.22 (2.08, 4.75)
	Obesity	21	2.56 (1.59, 3.92)	0	—	52	2.28 (1.70, 2.98)
CVD	Healthy	18,437	50.78 (50.05, 51.52)	18,437	50.78 (50.05, 51.52)	18,437	50.78 (50.05, 51.52)
	MASLD alone	19,306	98.15 (96.77, 99.55)	21,020	101.53 (100.16, 102.91)	16,441	89.97 (88.60, 91.36)
	MASLD + SRA	1791	160.72 (153.36, 168.34)	77	96.07 (75.82, 120.07)	4656	185.44 (180.15, 190.84)
	Non‐obesity	621	150.35 (138.75, 162.65)	61	93.43 (71.47, 120.01)	1241	201.12 (190.08, 212.63)
	Obesity	1170	166.83 (157.41, 176.67)	16	107.67 (61.54, 174.85)	3415	180.33 (174.33, 186.48)
CKD	Healthy	7967	21.27 (20.81, 21.74)	7967	21.27 (20.81, 21.74)	7967	21.27 (20.81, 21.74)
	MASLD alone	10,422	49.72 (48.77, 50.68)	11,619	52.57 (51.61, 53.53)	8444	43.73 (42.80, 44.67)
	MASLD + SRA	1248	101.58 (96.02, 107.37)	51	58.53 (43.58, 76.96)	3226	112.02 (108.19, 115.96)
	Non‐obesity	372	81.42 (73.36, 90.13)	46	64.97 (47.57, 86.66)	687	93.02 (86.19, 100.24)
	Obesity	876	113.51 (106.12, 121.28)	5	30.62 (9.94, 71.45)	2539	118.58 (114.01, 123.28)
Non‐liver cancers	Healthy	20,721	57.76 (56.98, 58.56)	20,721	57.76 (56.98, 58.56)	20,721	57.76 (56.98, 58.56)
	MASLD alone	16,435	82.44 (81.18, 83.71)	17,450	82.92 (81.69, 84.16)	14,876	81.10 (79.80, 82.41)
	MASLD + SRA	1084	91.07 (85.73, 96.65)	69	85.42 (66.46, 108.10)	2643	94.98 (91.39, 98.67)
	Non‐obesity	384	87.78 (79.22, 97.01)	57	86.29 (65.35, 111.79)	740	106.57 (99.02, 114.53)
	Obesity	700	92.98 (86.22, 100.13)	12	81.52 (42.12, 142.40)	1903	91.13 (87.08, 95.32)
Obesity‐related cancers	Healthy	6578	17.69 (17.27, 18.12)	6578	17.69 (17.27, 18.12)	6578	17.69 (17.27, 18.12)
	MASLD alone	5571	26.59 (25.89, 27.29)	5928	26.78 (26.11, 27.47)	4954	25.73 (25.02, 26.46)
	MASLD + SRA	387	30.62 (27.65, 33.83)	30	34.74 (23.44, 49.59)	1004	33.86 (31.80, 36.02)
	Non‐obesity	117	25.04 (20.71, 30.01)	24	34.01 (21.79, 50.60)	238	31.56 (27.68, 35.83)
	Obesity	270	33.89 (29.97, 38.19)	6	38.00 (13.94, 82.71)	766	34.64 (32.23, 37.19)

Abbreviations: MS, muscle strength; MM, muscle mass; PP, physical performance; NO, non‐obesity; O, obesity; HCC, hepatocellular carcinoma; CVD, cardiovascular disease; CKD, chronic kidney disease; PY, person‐years. SRA, sarcopenia‐related abnormalities.

When stratified by MS, MM, and PP, consistent trends were observed. Participants with MASLD and low MS, low MM, or low PP all exhibited substantially higher incidence rates of liver and non‐liver adverse outcomes compared with participants with MASLD and normal measures. The most pronounced differences were observed in obese participants with MASLD and low MS, with incidence rates several‐fold higher than those observed in healthy participants across liver and non‐liver outcomes (Table 2). Similarly, participants with slow PP had more than twofold higher incidence of CVD and CKD compared with those healthy participants (Table 2). Despite smaller sample sizes, low MM was also consistently associated with increased risks, particularly for cirrhosis, HCC, CKD, and all‐cause mortality (Table 2). These findings underscore that sarcopenia‐related abnormalities, even when assessed by simple measures such as grip strength or walking pace, identify MASLD participants at particularly high risk of adverse outcomes.

### 2.3 Associations Between MASLD and Sarcopenia‐Related Abnormalities With Clinical Outcomes

In multivariable‐adjusted Cox regression analyses (Tables S5–S7, Figure 2A–C), participants with MASLD and sarcopenia‐related abnormalities (low MS, low MM, or impaired PP) were consistently associated with higher risks of adverse outcomes than those with preserved muscle function. The strongest associations were observed for liver‐related outcomes. For example, MASLD with low MS was associated with a nearly eightfold higher risk of cirrhosis (aHR 7.36, 95% CI 6.00–9.02) and a fivefold higher risk of HCC (aHR 5.29, 95% CI 3.51–7.96) compared with healthy participants, with risks being particularly pronounced in the obese subgroup (cirrhosis aHR 8.48, 95% CI 6.73–10.68; HCC aHR 5.69, 95% CI 3.49–9.28). Impaired PP also conferred substantially increased risks, with aHR 7.55 (95% CI 6.45–8.83) for cirrhosis, aHR 5.03 (95% CI 3.66–6.92) for HCC, and aHR 1.83 (95% CI 1.75–1.92) for CKD, again with stronger effects in the obese subgroup. For CVD and CKD, participants with MASLD and low MS had a higher risk of CVD (aHR 1.31, 95% CI 1.25–1.38) and CKD (aHR 1.68, 95% CI 1.57–1.79). Impaired PP conferred the greatest risk for CVD (aHR 1.43, 95% CI 1.38–1.49). Associations with non‐liver and obesity‐related cancers were more modest, particularly for low MM, where estimates were imprecise with wide confidence intervals. Collectively, these findings highlight that sarcopenia‐related abnormalities markedly amplify the risks conferred by MASLD, especially for cirrhosis, HCC, and cardiometabolic complications, with the greatest excess risks observed in obese participants.

**FIGURE 2:**
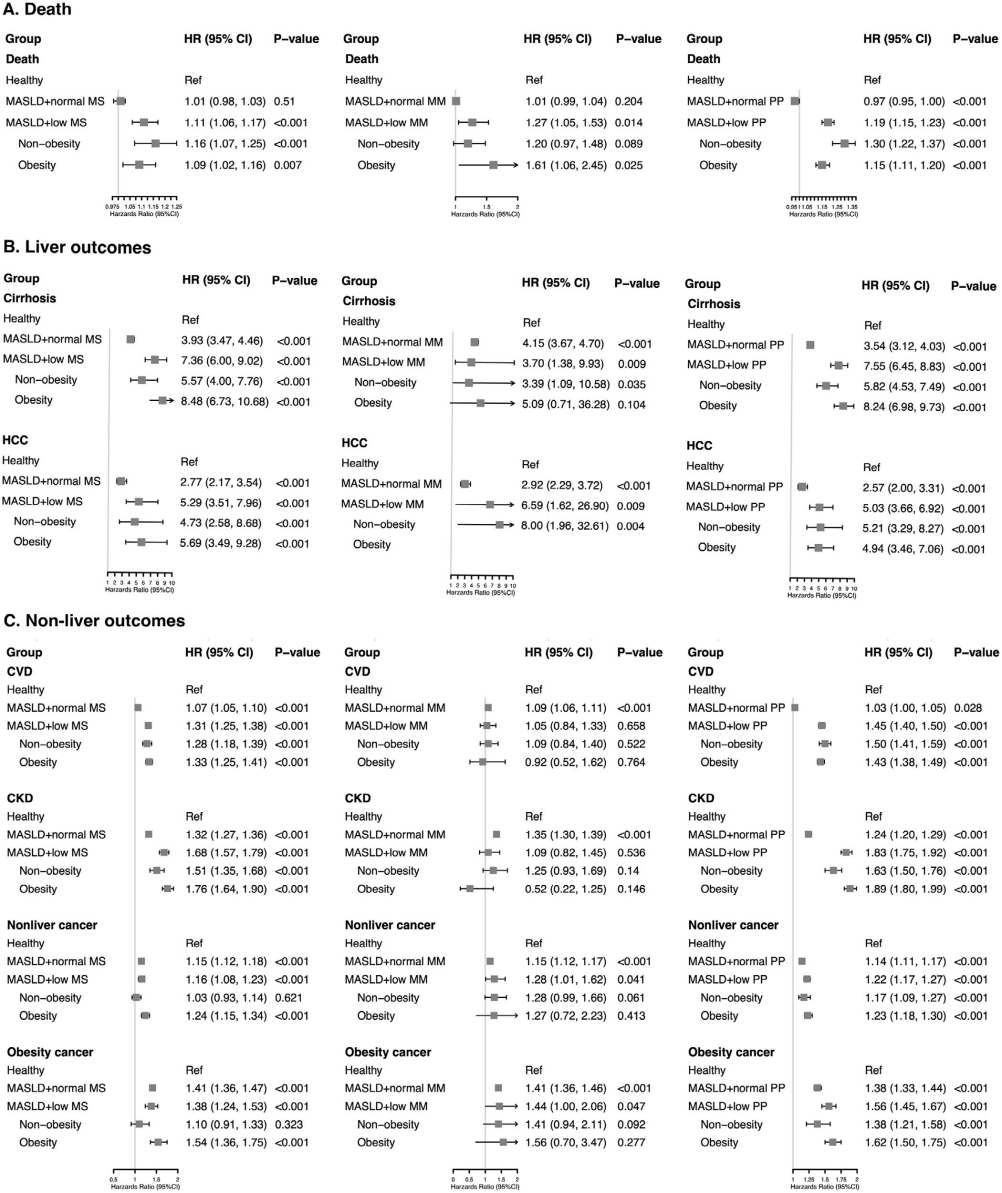
Association between combined MASLD/sarcopenia‐related abnormalities (including muscle strength [MS], muscle mass [MM], physical performance [PP]) status and clinical outcomes. (A) Death; (B) liver outcomes; (C) non‐liver outcomes. Forest plots show adjusted hazard ratios (HRs) with 95% confidence intervals (CIs) from Model 2. Model 2 was adjusted for age, sex, ethnicity, education level, and the Townsend deprivation index, as well as outcome‐specific covariates: the Charlson Comorbidity Index (CCI) for all‐cause mortality; the Fibrosis‐4 Index (FIB‐4) for cirrhosis, hepatocellular carcinoma (HCC), non‐liver cancer, and obesity‐related cancers; and FIB‐4 together with diabetes, hypertension, and hyperlipidemia for chronic kidney disease (CKD) and cardiovascular disease (CVD).

### 2.4 Subgroup and Sensitivity Analysis

Subgroup analyses showed that the stepwise trend of risk across groups remained consistent, with the highest hazards observed in MASLD with sarcopenia‐related abnormalities and more pronounced effects in obese participants (Tables 3, 4, 5, 6, S8–S19, and S23–S34). Older participants with coexisting MASLD and sarcopenia‐related abnormalities had two‐ to threefold higher incidence rates than younger participants, and for CVD and obesity‐related cancers, risks in older MASLD with low MM were nearly fivefold those of younger counterparts (Tables 3 and S23–S25). Men generally had about twice the incidence of adverse outcomes compared with women, with the disparity most striking for HCC, where rates in men were approximately fivefold those in women (Tables 4 and S26–S28). Participants with diabetes had about twofold higher risks (Tables 5 and S29–S31), while those with advanced fibrosis (FIB‐4 >2.67) exhibited particularly elevated risks for liver‐related outcomes, exceeding 20‐fold for cirrhosis and 30‐fold for HCC compared with those with FIB‐4 < 1.3 (Tables 6 and S32–S34). Cox regression analyses showed that the HRs associated with MASLD and sarcopenia‐related abnormalities were broadly similar across subgroups, consistent with the main analysis. Sensitivity analyses when restricted to White participants yielded results consistent with the main analysis (Tables S20–S22 and S35–S37). External validation using NHANES 2011–2018 supported the main findings from the UK Biobank (Tables S38–S39). Findings indicated that the associations were robust across subgroups and analytic approaches.

**TABLE 3:** Associations of combined MASLD and muscle strength (MS), muscle mass (MM), and physical performance (PP) status with clinical outcomes, stratified by age.

		MS, HR (95% CI)		MM, HR (95% CI)		PP, HR (95% CI)	
Outcomes	Groups	Younger	Older	Younger	Older	Younger	Older
Death	Healthy	Ref	Ref	Ref	Ref	Ref	Ref
	MASLD alone	1.03 (0.98, 1.08)	1.00 (0.98, 1.03)	1.03 (0.98, 1.08)	1.01 (0.99, 1.04)	1.01 (0.96, 1.07)	0.96 (0.94, 0.99)
	MASLD + SRA	1.05 (0.93, 1.19)	1.13 (1.07, 1.19)	1.41 (0.82, 2.43)	1.23 (1.01, 1.51)	1.09 (1.01, 1.19)	1.22 (1.18, 1.27)
	Non‐obesity	1.19 (0.96, 1.47)	1.18 (1.08, 1.28)	2.30 (1.27, 4.16)	1.15 (0.91, 1.44)	1.09 (0.95, 1.26)	1.33 (1.25, 1.42)
	Obesity	1.00 (0.86, 1.16)	1.10 (1.03, 1.18)	0.45 (0.11, 1.80)	1.73 (1.12, 2.69)	1.09 (1.00, 1.20)	1.18 (1.13, 1.23)
Cirrhosis	Healthy	Ref	Ref	Ref	Ref	Ref	Ref
	MASLD alone	3.44 (2.85, 4.16)	4.29 (3.63, 5.08)	3.61 (2.99, 4.35)	4.54 (3.84, 5.36)	3.18 (2.62, 3.86)	3.80 (3.19, 4.51)
	MASLD + SRA	6.89 (4.82, 9.86)	7.55 (5.85, 9.73)	—	4.86 (1.80, 13.11)	6.26 (4.82, 8.12)	8.32 (6.81, 10.17)
	Non‐obesity	6.67 (3.83, 11.60)	5.34 (3.53, 8.08)	—	4.43 (1.41, 13.89)	3.98 (2.45, 6.47)	7.00 (5.19, 9.44)
	Obesity	7.02 (4.58, 10.77)	8.98 (6.76, 11.92)	—	6.83 (0.95, 48.81)	7.16 (5.44, 9.43)	8.85 (7.16, 10.95)
HCC	Healthy	Ref	Ref	Ref	Ref	Ref	Ref
	MASLD alone	1.92 (1.20, 3.10)	3.13 (2.44, 4.01)	2.03 (1.27, 3.24)	3.28 (2.50, 4.32)	1.73 (1.05, 2.83)	2.93 (2.32, 3.70)
	MASLD + SRA	4.06 (1.54, 10.72)	5.63 (3.79, 8.37)	—	8.30 (2.07, 33.36)	4.04 (2.07, 7.90)	5.38 (4.04, 7.17)
	Non‐obesity	4.14 (0.97, 17.72)	5.11 (2.72, 9.60)	—	10.01 (2.49, 40.22)	4.57 (1.71, 12.23)	5.56 (3.49, 8.86)
	Obesity	4.01 (1.20, 13.39)	6.01 (3.68, 9.81)	—	—	3.80 (1.76, 8.23)	5.30 (3.79, 7.40)
CVD	Healthy	Ref	Ref	Ref	Ref	Ref	Ref
	MASLD alone	1.07 (1.02, 1.12)	1.06 (1.04, 1.09)	1.08 (1.04, 1.13)	1.08 (1.05, 1.11)	1.04 (1.00, 1.09)	1.01 (0.99, 1.04)
	MASLD + SRA	1.38 (1.23, 1.55)	1.29 (1.22, 1.37)	0.74 (0.28, 1.98)	1.07 (0.84, 1.37)	1.40 (1.30, 1.51)	1.46 (1.40, 1.52)
	Non‐obesity	1.49 (1.24, 1.80)	1.25 (1.14, 1.36)	0.81 (0.26, 2.50)	1.10 (0.85, 1.43)	1.55 (1.36, 1.76)	1.48 (1.38, 1.59)
	Obesity	1.33 (1.16, 1.53)	1.32 (1.23, 1.42)	0.60 (0.08, 4.24)	0.95 (0.52, 1.71)	1.35 (1.25, 1.47)	1.45 (1.39, 1.52)
CKD	Healthy	Ref	Ref	Ref	Ref	Ref	Ref
	MASLD alone	1.24 (1.15, 1.34)	1.33 (1.28, 1.38)	1.26 (1.17, 1.36)	1.36 (1.32, 1.41)	1.96 (1.82, 2.11)	1.26 (1.21, 1.31)
	MASLD + SRA	1.61 (1.36, 1.91)	1.69 (1.58, 1.81)	1.12 (0.36, 3.47)	1.11 (0.83, 1.49)	1.81 (1.62, 2.03)	1.83 (1.74, 1.92)
	Non‐obesity	1.49 (1.09, 2.02)	1.52 (1.35, 1.70)	1.02 (0.26, 4.10)	1.28 (0.94, 1.75)	3.79 (3.11, 4.63)	1.60 (1.47, 1.75)
	Obesity	1.66 (1.37, 2.01)	1.78 (1.64, 1.93)	1.37 (0.19, 9.73)	0.46 (0.17, 1.23)	5.08 (4.55, 5.67)	1.91 (1.80, 2.01)
Non‐liver cancers	Healthy	Ref	Ref	Ref	Ref	Ref	Ref
	MASLD alone	1.18 (1.14, 1.23)	1.14 (1.11, 1.17)	1.18 (1.14, 1.23)	1.14 (1.11, 1.17)	1.16 (1.12, 1.21)	1.13 (1.10, 1.16)
	MASLD + SRA	1.20 (1.04, 1.39)	1.15 (1.07, 1.24)	1.34 (0.67, 2.69)	1.35 (1.05, 1.74)	1.34 (1.23, 1.47)	1.18 (1.12, 1.23)
	Non‐obesity	0.93 (0.71, 1.22)	1.05 (0.94, 1.17)	1.04 (0.43, 2.51)	1.39 (1.06, 1.83)	1.25 (1.05, 1.48)	1.15 (1.05, 1.25)
	Obesity	1.36 (1.15, 1.62)	1.22 (1.12, 1.34)	2.59 (0.84, 8.04)	1.17 (0.61, 2.24)	1.38 (1.25, 1.52)	1.19 (1.12, 1.26)
Obesity‐related cancer	Healthy	Ref	Ref	Ref	Ref	Ref	Ref
	MASLD alone	1.48 (1.38, 1.59)	1.38 (1.32, 1.45)	1.49 (1.39, 1.60)	1.38 (1.31, 1.44)	1.44 (1.34, 1.55)	1.36 (1.29, 1.43)
	MASLD + SRA	1.64 (1.30, 2.06)	1.32 (1.17, 1.49)	0.47 (0.07, 3.33)	1.60 (1.11, 2.30)	1.84 (1.60, 2.11)	1.47 (1.36, 1.60)
	Non‐obesity	1.32 (0.86, 2.04)	1.06 (0.86, 1.30)	—	1.61 (1.08, 2.41)	1.47 (1.09, 1.99)	1.34 (1.16, 1.56)
	Obesity	1.80 (1.37, 2.36)	1.48 (1.29, 1.71)	2.23 (0.31, 15.83)	1.53 (0.63, 3.67)	1.94 (1.67, 2.26)	1.52 (1.39, 1.66)

*Note*: Younger and older participants were defined as those aged <57 years and ≥57 years, respectively, based on the median age of the study population. All estimates were derived from Model 2, which adjusted for age, sex, ethnicity, education level, and the Townsend deprivation index, with additional outcome‐specific adjustments for the Charlson Comorbidity Index (CCI) for all‐cause mortality; the Fibrosis‐4 Index (FIB‐4) for cirrhosis, HCC, non‐liver cancer, and obesity‐related cancer; and FIB‐4, diabetes, hypertension, and hyperlipidemia for CKD and CVD. The stratification variable was omitted from the corresponding adjusted model.

Abbreviations: MS, muscle strength; MM, muscle mass; PP, physical performance; HCC, hepatocellular carcinoma; CVD, cardiovascular disease; CKD, chronic kidney disease; SRA, sarcopenia‐related abnormalities; HR, hazard ratio; CI, confidence interval.

**TABLE 4:** Associations of combined MASLD and muscle strength (MS), muscle mass (MM), and physical performance (PP) status with clinical outcomes, stratified by sex.

		MS, HR (95% CI)		MM, HR (95% CI)		PP, HR (95% CI)	
Outcomes	Groups	Male	Female	Male	Female	Male	Female
Death	Healthy	Ref	Ref	Ref	Ref	Ref	Ref
	MASLD alone	1.01 (0.98, 1.04)	0.98 (0.95, 1.02)	1.02 (0.99, 1.05)	0.98 (0.94, 1.01)	0.98 (0.95, 1.01)	0.93 (0.90, 0.97)
	MASLD + SRA	2.06 (1.93, 2.19)	0.94 (0.87, 1.02)	1.71 (1.01, 2.90)	1.15 (0.94, 1.40)	1.24 (1.19, 1.30)	1.11 (1.05, 1.17)
	Non‐obesity	1.23 (1.12, 1.36)	1.05 (0.90, 1.23)	1.78 (1.05, 3.00)	1.06 (0.84, 1.34)	1.37 (1.28, 1.46)	1.19 (1.06, 1.33)
	Obesity	1.21 (1.11, 1.32)	0.92 (0.84, 1.00)	—	1.57 (1.03, 2.38)	1.18 (1.12, 1.24)	1.10 (1.03, 1.16)
Cirrhosis	Healthy	Ref	Ref	Ref	Ref	Ref	Ref
	MASLD alone	3.34 (2.85, 3.92)	4.94 (4.06, 6.02)	3.46 (2.95, 4.05)	5.35 (4.41, 6.49)	3.01 (2.55, 3.54)	4.61 (3.76, 5.65)
	MASLD + SRA	5.66 (4.30, 7.44)	10.30 (7.59, 13.97)	—	5.25 (1.94, 14.21)	6.73 (5.50, 8.24)	8.56 (6.68, 10.96)
	Non‐obesity	4.93 (3.35, 7.28)	6.27 (3.29, 11.95)	—	4.92 (1.56, 15.47)	5.04 (3.76, 6.77)	7.37 (4.49, 12.09)
	Obesity	6.29 (4.51, 8.77)	11.74 (8.49, 16.23)	—	6.56 (0.92, 46.98)	7.71 (6.20, 9.59)	8.80 (6.80, 11.39)
HCC	Healthy	Ref	Ref	Ref	Ref	Ref	Ref
	MASLD alone	3.29 (2.41, 4.50)	2.03 (1.31, 3.13)	3.48 (2.56, 4.74)	2.08 (1.36, 3.17)	3.06 (2.23, 4.21)	1.87 (1.18, 2.97)
	MASLD + SRA	7.01 (4.32, 11.35)	2.78 (1.17, 6.65)	19.34 (2.66, 140.49)	3.50 (0.48, 25.60)	6.05 (4.23, 8.66)	2.98 (1.60, 5.54)
	Non‐obesity	6.79 (3.60, 12.83)	NA (NA, NA)	20.58 (2.83, 149.51)	4.43 (0.61, 32.38)	6.51 (3.90, 10.86)	2.51 (0.60, 10.47)
	Obesity	7.20 (3.95, 13.12)	3.81 (1.59, 9.10)	—	—	6.38 (4.12, 9.89)	3.07 (1.59, 5.92)
CVD	Healthy	Ref	Ref	Ref	Ref	Ref	Ref
	MASLD alone	1.05 (1.02, 1.08)	1.09 (1.05, 1.13)	1.06 (1.03, 1.09)	1.12 (1.08, 1.16)	1.02 (0.99, 1.05)	1.02 (0.98, 1.06)
	MASLD + SRA	1.23 (1.15, 1.32)	1.40 (1.29, 1.51)	0.87 (0.42, 1.84)	1.06 (0.83, 1.36)	1.40 (1.34, 1.47)	1.51 (1.43, 1.59)
	Non‐obesity	1.21 (1.10, 1.34)	1.43 (1.24, 1.65)	1.00 (0.48, 2.09)	1.08 (0.82, 1.42)	1.39 (1.30, 1.50)	1.83 (1.64, 2.05)
	Obesity	1.25 (1.15, 1.37)	1.39 (1.27, 1.52)	—	0.98 (0.56, 1.72)	1.40 (1.33, 1.48)	1.45 (1.37, 1.54)
CKD	Healthy	Ref	Ref	Ref	Ref	Ref	Ref
	MASLD alone	1.25 (1.19, 1.31)	1.40 (1.34, 1.47)	1.27 (1.21, 1.33)	1.43 (1.37, 1.50)	1.20 (1.15, 1.26)	1.29 (1.22, 1.35)
	MASLD + SRA	1.63 (1.49, 1.79)	1.71 (1.57, 1.87)	1.33 (1.20, 1.48)	1.13 (0.84, 1.52)	1.33 (1.20, 1.48)	1.97 (1.84, 2.10)
	Non‐obesity	1.54 (1.34, 1.76)	1.40 (1.17, 1.69)	1.32 (0.50, 3.53)	1.29 (0.94, 1.77)	1.54 (1.39, 1.71)	1.78 (1.55, 2.05)
	Obesity	1.70 (1.52, 1.90)	1.82 (1.65, 2.00)	—	0.57 (0.24, 1.37)	1.78 (1.66, 1.92)	2.00 (1.87, 2.14)
Non‐liver cancers	Healthy	Ref	Ref	Ref	Ref	Ref	Ref
	MASLD alone	1.09 (1.06, 1.12)	1.29 (1.24, 1.34)	1.09 (1.06, 1.12)	1.28 (1.24, 1.33)	1.09 (1.06, 1.12)	1.26 (1.21, 1.31)
	MASLD + SRA	1.10 (1.01, 1.19)	1.26 (1.14, 1.39)	0.85 (0.41, 1.79)	1.51 (1.18, 1.94)	1.11 (1.05, 1.17)	1.40 (1.31, 1.50)
	Non‐obesity	1.03 (0.92, 1.16)	0.94 (0.75, 1.16)	0.91 (0.43, 1.91)	1.52 (1.15, 2.00)	1.09 (1.00, 1.19)	1.40 (1.20, 1.63)
	Obesity	1.16 (1.04, 1.29)	1.38 (1.23, 1.54)	—	1.51 (0.86, 2.66)	1.12 (1.05, 1.19)	1.40 (1.31, 1.51)
Obesity‐related cancer	Healthy	Ref	Ref	Ref	Ref	Ref	Ref
	MASLD alone	1.36 (1.29, 1.44)	1.48 (1.40, 1.56)	1.36 (1.29, 1.44)	1.46 (1.38, 1.54)	1.35 (1.28, 1.43)	1.42 (1.34, 1.50)
	MASLD + SRA	1.47 (1.27, 1.70)	1.29 (1.10, 1.50)	0.55 (0.08, 3.90)	1.61 (1.12, 2.32)	1.45 (1.31, 1.61)	1.65 (1.50, 1.82)
	Non‐obesity	1.26 (1.01, 1.57)	0.82 (0.57, 1.17)	0.58 (0.08, 4.11)	1.59 (1.06, 2.40)	1.32 (1.12, 1.56)	1.44 (1.13, 1.82)
	Obesity	1.67 (1.38, 2.01)	1.47 (1.24, 1.73)	—	1.69 (0.76, 3.78)	1.52 (1.35, 1.72)	1.69 (1.53, 1.87)

*Note*: All estimates were derived from Model 2, which adjusted for age, sex, ethnicity, education level, and the Townsend deprivation index, with additional outcome‐specific adjustments for the Charlson Comorbidity Index (CCI) for all‐cause mortality; the Fibrosis‐4 Index (FIB‐4) for cirrhosis, HCC, non‐liver cancer, and obesity‐related cancer; and FIB‐4, diabetes, hypertension, and hyperlipidemia for CKD and CVD. The stratification variable was omitted from the corresponding adjusted model.

Abbreviations: MS, muscle strength; MM, muscle mass; PP, physical performance; HCC, hepatocellular carcinoma; CVD, cardiovascular disease; CKD, chronic kidney disease; SRA, sarcopenia‐related abnormalities; HR, hazard ratio; CI, confidence interval.

**TABLE 5:** Associations of combined MASLD and muscle strength (MS), muscle mass (MM), and physical performance (PP) status with clinical outcomes, stratified by diabetes.

		MS, HR (95% CI)		MM, HR (95% CI)		PP, HR (95% CI)	
Outcomes	Groups	DM	Non‐DM	DM	Non‐DM	DM	Non‐DM
Death	Healthy	Ref	Ref	Ref	Ref	Ref	Ref
	MASLD alone	1.04 (0.98, 1.10)	1.06 (1.03, 1.09)	1.06 (1.00, 1.12)	1.07 (1.04, 1.10)	0.96 (0.91, 1.02)	1.01 (0.99, 1.04)
	MASLD + SRA	1.23 (1.12, 1.34)	1.18 (1.11, 1.26)	1.56 (1.01, 2.39)	1.09 (0.88, 1.34)	1.31 (1.22, 1.40)	1.35 (1.29, 1.41)
	Non‐obesity	1.28 (1.10, 1.48)	1.12 (1.02, 1.23)	1.53 (0.95, 2.46)	1.02 (0.81, 1.29)	1.37 (1.22, 1.52)	1.27 (1.19, 1.36)
	Obesity	1.21 (1.10, 1.34)	1.23 (1.13, 1.34)	1.69 (0.63, 4.51)	1.44 (0.91, 2.29)	1.29 (1.20, 1.39)	1.40 (1.33, 1.47)
Cirrhosis	Healthy	Ref	Ref	Ref	Ref	Ref	Ref
	MASLD alone	2.91 (2.18, 3.89)	2.73 (2.36, 3.16)	3.06 (2.30, 4.08)	2.82 (2.44, 3.26)	2.65 (1.97, 3.55)	2.60 (2.24, 3.02)
	MASLD + SRA	4.64 (3.27, 6.57)	4.25 (3.12, 5.80)	6.07 (1.89, 19.53)	1.29 (0.18, 9.20)	4.35 (3.19, 5.91)	4.27 (3.42, 5.33)
	Non‐obesity	4.03 (2.40, 6.78)	3.99 (2.52, 6.30)	5.11 (1.24, 21.07)	1.54 (0.22, 11.02)	3.61 (2.35, 5.56)	4.47 (3.22, 6.21)
	Obesity	4.85 (3.36, 7.01)	4.46 (3.02, 6.60)	9.73 (1.34, 70.75)	—	4.52 (3.30, 6.18)	4.17 (3.21, 5.41)
HCC	Healthy	Ref	Ref	Ref	Ref	Ref	Ref
	MASLD alone	2.56 (1.50, 4.37)	1.70 (1.28, 2.25)	2.57 (1.51, 4.39)	1.84 (1.39, 2.45)	2.47 (1.44, 4.26)	1.65 (1.24, 2.18)
	MASLD + SRA	2.64 (1.28, 5.45)	4.87 (2.96, 8.01)	—	8.65 (2.15, 34.85)	2.85 (1.57, 5.16)	3.55 (2.38, 5.29)
	Non‐obesity	1.85 (0.53, 6.42)	5.49 (2.82, 10.73)	—	10.38 (2.58, 41.81)	2.92 (1.30, 6.54)	4.88 (2.84, 8.41)
	Obesity	2.96 (1.37, 6.40)	4.32 (2.13, 8.77)	—	—	2.83 (1.53, 5.23)	2.80 (1.63, 4.82)
CVD	Healthy	Ref	Ref	Ref	Ref	Ref	Ref
	MASLD alone	1.06 (1.00, 1.12)	1.07 (1.04, 1.09)	1.08 (1.02, 1.14)	1.08 (1.05, 1.11)	0.99 (0.93, 1.05)	1.02 (1.00, 1.05)
	MASLD + SRA	1.31 (1.20, 1.43)	1.30 (1.21, 1.39)	1.54 (1.00, 2.38)	0.90 (0.68, 1.19)	1.39 (1.30, 1.49)	1.47 (1.41, 1.54)
	Non‐obesity	1.19 (1.02, 1.38)	1.32 (1.19, 1.45)	1.62 (1.00, 2.61)	0.93 (0.69, 1.26)	1.34 (1.20, 1.50)	1.56 (1.45, 1.68)
	Obesity	1.36 (1.23, 1.49)	1.29 (1.18, 1.40)	1.30 (0.49, 3.46)	0.77 (0.39, 1.55)	1.41 (1.31, 1.51)	1.43 (1.36, 1.51)
CKD	Healthy	Ref	Ref	Ref	Ref	Ref	Ref
	MASLD alone	1.28 (1.19, 1.37)	1.32 (1.27, 1.37)	1.32 (1.23, 1.41)	1.35 (1.30, 1.40)	1.17 (1.09, 1.26)	1.25 (1.20, 1.30)
	MASLD + SRA	1.66 (1.50, 1.84)	1.65 (1.51, 1.81)	1.03 (0.60, 1.78)	1.09 (0.78, 1.52)	1.74 (1.61, 1.89)	1.85 (1.74, 1.96)
	Non‐obesity	1.38 (1.15, 1.64)	1.58 (1.37, 1.82)	1.27 (0.72, 2.25)	1.21 (0.85, 1.72)	1.54 (1.35, 1.76)	1.63 (1.46, 1.81)
	Obesity	1.77 (1.58, 1.97)	1.70 (1.53, 1.90)	0.31 (0.04, 2.24)	0.61 (0.23, 1.63)	1.79 (1.64, 1.94)	1.94 (1.81, 2.07)
Non‐liver cancers	Healthy	Ref	Ref	Ref	Ref	Ref	Ref
	MASLD alone	0.99 (0.93, 1.06)	1.09 (1.06, 1.11)	0.99 (0.92, 1.05)	1.09 (1.06, 1.11)	1.00 (0.94, 1.07)	1.08 (1.05, 1.11)
	MASLD + SRA	0.92 (0.82, 1.04)	1.08 (1.00, 1.17)	1.12 (0.65, 1.93)	1.24 (0.95, 1.61)	0.94 (0.86, 1.02)	1.13 (1.08, 1.20)
	Non‐obesity	0.89 (0.72, 1.09)	0.98 (0.87, 1.10)	1.22 (0.67, 2.21)	1.22 (0.92, 1.63)	0.89 (0.76, 1.04)	1.16 (1.06, 1.27)
	Obesity	0.94 (0.82, 1.07)	1.17 (1.06, 1.29)	0.76 (0.19, 3.06)	1.33 (0.72, 2.48)	0.95 (0.87, 1.04)	1.12 (1.05, 1.20)
Obesity‐related cancer	Healthy	Ref	Ref	Ref	Ref	Ref	Ref
	MASLD alone	1.25 (1.12, 1.40)	1.27 (1.21, 1.32)	1.24 (1.11, 1.39)	1.26 (1.21, 1.32)	1.26 (1.12, 1.41)	1.25 (1.19, 1.31)
	MASLD + SRA	1.16 (0.96, 1.40)	1.16 (1.01, 1.33)	2.22 (1.18, 4.16)	1.14 (0.73, 1.76)	1.20 (1.04, 1.38)	1.34 (1.23, 1.47)
	Non‐obesity	0.83 (0.57, 1.21)	1.06 (0.85, 1.32)	2.26 (1.12, 4.55)	1.11 (0.68, 1.81)	1.04 (0.80, 1.34)	1.32 (1.13, 1.55)
	Obesity	1.28 (1.05, 1.57)	1.24 (1.04, 1.47)	2.08 (0.52, 8.35)	1.28 (0.48, 3.40)	1.24 (1.07, 1.43)	1.35 (1.22, 1.50)

*Note*: All estimates were derived from Model 2, which adjusted for age, sex, ethnicity, education level, and the Townsend deprivation index, with additional outcome‐specific adjustments for the Charlson Comorbidity Index (CCI) for all‐cause mortality; the Fibrosis‐4 Index (FIB‐4) for cirrhosis, HCC, non‐liver cancer, and obesity‐related cancer; and FIB‐4, diabetes, hypertension, and hyperlipidemia for CKD and CVD. The stratification variable was omitted from the corresponding adjusted model.

Abbreviations: MS, muscle strength; MM, muscle mass; PP, physical performance; HCC, hepatocellular carcinoma; CVD, cardiovascular disease; CKD, chronic kidney disease; SRA, sarcopenia‐related abnormalities; HR, hazard ratio; CI, confidence interval.

**TABLE 6:** Associations of combined MASLD and muscle strength (MS), muscle mass (MM), and physical performance (PP) status with clinical outcomes, stratified by FIB‐4.

		MS, HR (95% CI)			MM, HR (95% CI)			PP, HR (95% CI)		
Outcomes	Groups	FIB‐4 < 1.3	1.3 ≤ FIB‐4 ≤ 2.67	FIB‐4 > 2.67	FIB‐4 < 1.3	1.3 ≤ FIB‐4 ≤ 2.67	FIB‐4 > 2.67	FIB‐4 < 1.3	1.3 ≤ FIB‐4 ≤ 2.67	FIB‐4 > 2.67
Death	Healthy	Ref	Ref	Ref	Ref	Ref	Ref	Ref	Ref	Ref
	MASLD alone	1.03 (1.00, 1.07)	1.00 (0.97, 1.03)	1.18 (1.06, 1.31)	1.39 (1.34, 1.44)	1.02 (0.99, 1.05)	1.17 (1.06, 1.30)	1.01 (0.97, 1.05)	0.97 (0.94, 1.00)	1.11 (0.99, 1.24)
	MASLD + SRA	1.05 (0.96, 1.14)	1.25 (1.16, 1.34)	1.18 (0.96, 1.45)	1.21 (0.92, 1.59)	1.28 (0.95, 1.71)	2.02 (1.00, 4.09)	1.13 (1.07, 1.20)	1.24 (1.18, 1.31)	1.47 (1.27, 1.70)
	Non‐obesity	1.10 (0.96, 1.25)	1.36 (1.22, 1.52)	1.06 (0.79, 1.44)	1.76 (1.29, 2.40)	1.32 (0.97, 1.79)	1.10 (0.41, 2.95)	1.19 (1.08, 1.30)	1.41 (1.30, 1.53)	1.50 (1.21, 1.86)
	Obesity	1.02 (0.93, 1.13)	1.19 (1.09, 1.30)	1.27 (0.99, 1.64)	2.09 (1.21, 3.61)	0.97 (0.36, 2.58)	12.37 (4.59, 33.31)	1.11 (1.05, 1.19)	1.18 (1.12, 1.25)	1.45 (1.23, 1.72)
Cirrhosis	Healthy	Ref	Ref	Ref	Ref	Ref	Ref	Ref	Ref	Ref
	MASLD alone	3.01 (2.36, 3.84)	5.38 (4.49, 6.45)	6.30 (4.83, 8.21)	3.27 (2.57, 4.15)	5.59 (4.67, 6.69)	6.55 (5.03, 8.52)	2.69 (2.09, 3.46)	4.78 (3.97, 5.76)	5.97 (4.56, 7.81)
	MASLD + SRA	7.57 (5.09, 11.25)	8.47 (6.23, 11.51)	9.04 (6.12, 13.34)	—	5.69 (1.41, 23.01)	4.44 (1.08, 18.24)	6.75 (4.95, 9.20)	10.05 (8.02, 12.60)	9.03 (6.55, 12.45)
	Non‐obesity	4.96 (2.48, 9.93)	6.17 (3.62, 10.51)	9.36 (5.41, 16.21)	—	6.83 (1.69, 27.59)	2.52 (0.35, 18.22)	4.70 (2.76, 8.00)	7.92 (5.49, 11.42)	6.83 (4.27, 10.92)
	Obesity	9.15 (5.90, 14.18)	9.76 (6.92, 13.77)	8.85 (5.62, 13.95)	—	—	18.80 (2.57, 137.32)	7.51 (5.43, 10.39)	10.85 (8.55, 13.78)	10.20 (7.23, 14.38)
HCC	Healthy	Ref	Ref	Ref	Ref	Ref	Ref	Ref	Ref	Ref
	MASLD alone	1.10 (0.65, 1.85)	3.19 (2.25, 4.51)	7.84 (4.51, 13.62)	1.22 (0.74, 2.01)	3.23 (2.29, 4.57)	8.37 (4.83, 14.48)	1.20 (0.71, 2.02)	2.60 (1.80, 3.74)	8.08 (4.63, 14.09)
	MASLD + SRA	3.49 (1.49, 8.21)	4.32 (2.22, 8.41)	14.05 (6.68, 29.55)	7.02 (0.94, 52.40)	8.99 (1.23, 65.61)	—	1.58 (0.70, 3.57)	7.00 (4.54, 10.79)	9.43 (4.81, 18.49)
	Non‐obesity	3.81 (1.13, 12.86)	4.89 (1.93, 12.39)	9.41 (3.09, 28.62)	8.98 (1.20, 66.95)	10.48 (1.44, 76.43)	—	2.74 (0.93, 8.10)	5.91 (3.01, 11.60)	10.41 (4.47, 24.25)
	Obesity	3.29 (1.13, 9.56)	3.95 (1.67, 9.29)	17.58 (7.75, 39.84)	—	—	—	1.12 (0.39, 3.26)	7.45 (4.68, 11.87)	8.87 (4.16, 18.90)
CVD	Healthy	Ref	Ref	Ref	Ref	Ref	Ref	Ref	Ref	Ref
	MASLD alone	1.09 (1.05, 1.13)	1.07 (1.04, 1.11)	1.05 (0.93, 1.18)	1.11 (1.07, 1.14)	1.09 (1.06, 1.12)	1.08 (0.96, 1.21)	1.05 (1.01, 1.09)	1.02 (0.99, 1.06)	1.00 (0.89, 1.13)
	MASLD + SRA	1.36 (1.26, 1.47)	1.29 (1.19, 1.38)	1.30 (1.02, 1.66)	1.14 (0.84, 1.56)	1.04 (0.73, 1.50)	0.31 (0.04, 2.21)	1.43 (1.36, 1.51)	1.49 (1.41, 1.56)	1.47 (1.22, 1.76)
	Non‐obesity	1.36 (1.21, 1.54)	1.22 (1.09, 1.38)	1.39 (0.95, 2.02)	1.21 (0.86, 1.70)	1.04 (0.70, 1.54)	0.40 (0.06, 2.87)	1.50 (1.37, 1.63)	1.53 (1.41, 1.67)	1.43 (1.07, 1.91)
	Obesity	1.36 (1.24, 1.49)	1.32 (1.21, 1.45)	1.26 (0.93, 1.69)	0.92 (0.44, 1.93)	1.07 (0.45, 2.58)	—	1.41 (1.33, 1.50)	1.47 (1.39, 1.56)	1.49 (1.21, 1.83)
CKD	Healthy	Ref	Ref	Ref	Ref	Ref	Ref	Ref	Ref	Ref
	MASLD alone	1.35 (1.28, 1.43)	1.35 (1.29, 1.41)	1.39 (1.19, 1.63)	1.39 (1.32, 1.47)	1.37 (1.31, 1.43)	1.45 (1.24, 1.69)	1.28 (1.21, 1.35)	1.28 (1.22, 1.34)	1.30 (1.10, 1.53)
	MASLD + SRA	1.79 (1.63, 1.98)	1.64 (1.50, 1.79)	1.96 (1.49, 2.57)	1.14 (0.76, 1.70)	1.19 (0.79, 1.80)	0.49 (0.07, 3.53)	1.89 (1.76, 2.03)	1.84 (1.73, 1.96)	2.13 (1.73, 2.64)
	Non‐obesity	1.75 (1.49, 2.05)	1.33 (1.14, 1.57)	2.33 (1.56, 3.47)	1.34 (0.87, 2.06)	1.31 (0.85, 2.01)	0.65 (0.09, 4.62)	1.74 (1.54, 1.97)	1.60 (1.43, 1.80)	1.89 (1.35, 2.65)
	Obesity	1.81 (1.62, 2.03)	1.80 (1.62, 2.00)	1.77 (1.26, 2.47)	0.55 (0.18, 1.71)	0.61 (0.15, 2.45)	—	1.94 (1.79, 2.09)	1.92 (1.79, 2.06)	2.24 (1.77, 2.83)
Non‐liver cancers	Healthy	Ref	Ref	Ref	Ref	Ref	Ref	Ref	Ref	Ref
	MASLD alone	1.15 (1.11, 1.19)	1.12 (1.09, 1.16)	1.21 (1.07, 1.36)	1.15 (1.11, 1.19)	1.12 (1.09, 1.16)	1.22 (1.08, 1.37)	1.14 (1.10, 1.17)	1.12 (1.09, 1.16)	1.21 (1.07, 1.38)
	MASLD + SRA	1.14 (1.04, 1.25)	1.12 (1.02, 1.23)	1.43 (1.08, 1.88)	1.27 (0.92, 1.74)	1.11 (0.74, 1.65)	2.48 (1.10, 5.58)	1.26 (1.18, 1.34)	1.14 (1.07, 1.21)	1.28 (1.04, 1.58)
	Non‐obesity	0.96 (0.82, 1.13)	1.03 (0.90, 1.19)	1.34 (0.87, 2.05)	1.28 (0.90, 1.83)	1.16 (0.77, 1.77)	1.94 (0.72, 5.22)	1.16 (1.04, 1.30)	1.13 (1.01, 1.26)	1.53 (1.11, 2.09)
	Obesity	1.25 (1.12, 1.40)	1.18 (1.05, 1.32)	1.49 (1.05, 2.10)	1.20 (0.60, 2.40)	0.72 (0.18, 2.87)	5.57 (1.38, 22.44)	1.29 (1.21, 1.39)	1.14 (1.06, 1.23)	1.16 (0.90, 1.50)
Obesity‐related cancers	Healthy	Ref	Ref	Ref	Ref	Ref	Ref	Ref	Ref	Ref
	MASLD alone	1.43 (1.35, 1.51)	1.37 (1.29, 1.45)	1.71 (1.40, 2.09)	1.42 (1.34, 1.50)	1.37 (1.29, 1.45)	1.73 (1.42, 2.10)	1.39 (1.31, 1.47)	1.35 (1.27, 1.43)	1.74 (1.42, 2.13)
	MASLD + SRA	1.31 (1.12, 1.53)	1.34 (1.15, 1.57)	2.25 (1.51, 3.36)	1.39 (0.85, 2.27)	1.06 (0.55, 2.04)	5.42 (2.20, 13.33)	1.62 (1.47, 1.79)	1.45 (1.31, 1.62)	1.84 (1.33, 2.52)
	Non‐obesity	1.07 (0.80, 1.42)	1.03 (0.78, 1.36)	1.95 (1.03, 3.70)	1.44 (0.84, 2.49)	0.96 (0.46, 2.01)	5.32 (1.95, 14.49)	1.33 (1.09, 1.63)	1.32 (1.09, 1.61)	2.17 (1.36, 3.47)
	Obesity	1.44 (1.20, 1.73)	1.55 (1.29, 1.86)	2.46 (1.52, 3.97)	1.18 (0.38, 3.67)	1.72 (0.43, 6.89)	5.86 (0.82, 42.08)	1.71 (1.53, 1.91)	1.50 (1.33, 1.69)	1.67 (1.14, 2.46)

*Note*: All estimates were derived from Model 2, which adjusted for age, sex, ethnicity, education level, and the Townsend deprivation index, with additional outcome‐specific adjustments for the Charlson Comorbidity Index (CCI) for all‐cause mortality; the Fibrosis‐4 Index (FIB‐4) for cirrhosis, HCC, non‐liver cancer, and obesity‐related cancer; and FIB‐4, diabetes, hypertension, and hyperlipidemia for CKD and CVD. The stratification variable was omitted from the corresponding adjusted model.

Abbreviations: MS, muscle strength; MM, muscle mass; PP, physical performance; HCC, hepatocellular carcinoma; CVD, cardiovascular disease; CKD, chronic kidney disease; SRA, sarcopenia‐related abnormalities; HR, hazard ratio; CI, confidence interval.

## 3 Discussion

In this large population‐based prospective cohort, we found that the coexistence of MASLD and sarcopenia‐related abnormalities was associated with markedly higher risks of a wide spectrum of adverse outcomes, including cirrhosis, HCC, CVD, CKD, non‐liver cancers, obesity‐related cancers, and all‐cause mortality. Importantly, a stepwise trend of association between sarcopenia‐related abnormalities, obesity, and MASLD phenotypes with adverse outcomes was observed, with the lowest risk in healthy participants, intermediate risk in MASLD without sarcopenia‐related abnormalities, and the highest risk in MASLD with sarcopenia‐related abnormalities. These findings highlight the joint association of metabolic liver disease and impaired muscle health with adverse clinical outcomes. Among the different phenotypes, sarcopenic obesity emerged as the most detrimental, with most hazards falling within the two‐ to fourfold range compared with MASLD alone across a wide range of adverse clinical outcomes.

Accumulating evidence has demonstrated strong links between both MASLD and muscle‐related abnormalities and a wide range of adverse health outcomes [20, 21, 22, 23, 24, 25, 26, 27, 28, 29]. For instance, data from the UK Biobank showed that faster walking speed, whether self‐reported or objectively measured, was associated with a reduced risk of cancer development in the general population [24, 25]. In participants with type 2 diabetes, sarcopenic obesity—defined as low muscle strength combined with obesity—was associated with a higher risk of heart failure, with metabolomics and inflammation partially mediating this association; additionally, grip strength showed a linear inverse relationship with mortality, whereas muscle mass displayed a U‐shaped association [26, 27]. In participants with MASLD, a decrease in handgrip strength was significantly associated with an increased risk of CVD [28]. Furthermore, a recent meta‐analysis including 29 studies and 63,330 participants reported that sarcopenia was strongly associated with MASLD progression and served as an independent risk factor not only for disease pathogenesis but also for MASLD‐related mortality [29]. However, a comprehensive evaluation of the clinical impact of coexisting MASLD and different sarcopenia‐related abnormalities remains lacking, particularly with diverse obesity states.

Our study demonstrates that MASLD with obesity and sarcopenia‐related abnormalities constitutes a particularly high‐risk phenotype, with consistently elevated incidence rates and HRs across nearly all studied outcomes. This observation is consistent with prior evidence indicating that obesity does not attenuate the adverse consequences of sarcopenia but rather exacerbates them [30, 31]. Mechanistically, sarcopenic obesity has been linked to fat infiltration into skeletal muscle, reduced muscle quality, impaired function, and worsening metabolic derangements. These abnormalities are integral to the “multiple‐hit” hypothesis of MASLD pathogenesis, involving insulin resistance, vitamin D deficiency, and chronic low‐grade inflammation [9, 10]. Notably, our findings also underscore that sarcopenia‐related abnormalities are clinically relevant even in non‐obese participants with MASLD, although the magnitude of risk is lower than in sarcopenic obesity. This is also in line with prior studies showing that lean MASLD with sarcopenia is associated with increased risks of CVD and advanced fibrosis [32, 33]. A plausible explanation is that participants with central adiposity despite low BMI frequently exhibit muscle loss and reduced bone mass, which further heightens vulnerability to cardiometabolic disorders and fractures [11, 34, 35]. Collectively, these results raise the possibility that impaired muscle health and metabolic liver disease may interact synergistically to accelerate adverse outcomes in individuals with MASLD, obesity, and sarcopenia‐related abnormalities.

Our study builds upon these findings by comprehensively evaluating all three dimensions of muscle health, including strength, mass, and performance, within the population with MASLD, and demonstrating that each dimension was independently associated with a broad range of adverse long‐term outcomes, including both liver and non‐liver outcomes as well as all‐cause mortality. To our knowledge, this is the first large‐scale prospective study to systematically investigate how the coexistence of MASLD and muscle‐related abnormalities influences relevant long‐term outcomes. These findings have important clinical implications. First, muscle health assessment, including grip strength, skeletal muscle mass, and physical performance, may provide additional prognostic value beyond traditional metabolic risk factors. Second, early identification of sarcopenia‐related abnormalities in MASLD patients could refine risk stratification and guide preventive strategies. Third, interventions such as lifestyle modification, nutritional supplementation, resistance training, and emerging pharmacological therapies targeting sarcopenic obesity warrant further investigation in this high‐risk population. Notably, prior studies have also shown that lifestyle interventions are associated with improvements in muscle strength [36], supporting their integration into management strategies.

Though our study was performed on a well‐characterized, prospective cohort with long‐term follow‐up and adjudicated outcomes, several limitations should be acknowledged. First, MASLD and sarcopenia‐related abnormalities were defined using surrogate markers rather than gold‐standard assessments such as MRI‐PDFF for liver fat or DXA for muscle mass. Although these surrogate markers are validated and widely used in epidemiologic research, misclassification remains possible. Second, probable sarcopenia was primarily defined by grip strength, which may underestimate muscle impairment in obese participants who typically have greater grip strength despite reduced muscle quality. Third, generalizability may be limited by the predominantly White population of the UK Biobank, so additional studies involving participants of other racial and ethnic backgrounds are needed.

In conclusion, our findings emphasize coexisting of MASLD and sarcopenia‐related abnormalities was associated with a substantially higher risk of both liver‐related and extrahepatic outcomes, particularly in obese participants. These results call for greater clinical attention to muscle health in metabolic liver disease and support integrating sarcopenia screening into risk assessment frameworks for chronic disease prevention and management.

## 4 Materials and Methods

### 4.1 Study Population

This study was conducted based on the UK Biobank, which is a large‐scale prospective cohort study recruited over 0.5 million participants, aged 40–70 years, in the United Kingdom during 2006–2010 [37]. Our initial sample comprised 502,389 participants at baseline. After excluding those without sufficient data to define MASLD or sarcopenia‐related abnormalities (*n* = 22,997) and those without MASLD but with sarcopenia‐related abnormalities (*n* = 40,644), a total of 438,748 participants were included in the final analysis. To minimize reverse causality, seven sub‐populations were generated by excluding participants with pre‐existing diagnoses of cirrhosis, HCC, CVD, CKD, non‐liver cancers, or obesity‐related cancers at baseline and within the first 6 months of follow‐up. This resulted in 438,483 participants for the mortality analysis, 438,482 for cirrhosis, 438,728 for HCC, 429,581 for non‐liver cancers, 435,704 for obesity‐related cancers, 429,495 for CVD, and 438,278 for CKD.

### 4.2 Assessment of MASLD and Obesity

For all participants, hepatic steatosis was identified using the Fatty Liver Index (FLI), a validated prediction widely used in epidemiological studies. The FLI was calculated according to the original equation proposed by Bedogni et al. using body mass index (BMI), waist circumference, triglycerides, and gamma‐glutamyl transferase (GGT). An FLI >60 was used to define SLD [38]. Participants with SLD who had at least one cardiometabolic risk factor, in the absence of excessive alcohol consumption (≤20 g/d in women and ≤30 g/d in men) and other causes of liver disease (e.g., viral hepatitis, autoimmune liver diseases), were diagnosed as having MASLD [1]. Obesity was defined using ethnicity‐specific BMI thresholds: BMI ≥30 kg/m^2^ for non‐Asian participants and BMI ≥25 kg/m^2^ for Asian participants, according to the WHO recommendations [39, 40].

### 4.3 Sarcopenia Definition

Sarcopenia is currently defined by a stepwise approach incorporating three key components: MS, MM, and PP. The presence of probable sarcopenia and sarcopenia were defined using the European Working Group of Sarcopenia in Older People 2 (EWGSOP2) definition [15] for participants of all ages. Participants were considered to have probable sarcopenia if they had low MS (max handgrip strength <27 kg in men and <16 kg in women) alone and confirmed sarcopenia if they also had low MM (<7.0 kg/m^2^ in men and <5.5 kg/m^2^ in women). Sarcopenia was considered to be severe when low MS, low MM and low PP were all present. MS was assessed using handgrip strength, measured with a Jamar J00105 hydraulic handheld dynamometer. MM was assessed using the skeletal muscle index (SMI), derived from whole‐body skeletal muscle mass (SMM) estimated by bioelectrical impedance analysis (BIA; Tanita BC418MA, Tokyo, Japan) and calculated via the Janssen equation [41]: SMM (kg) = [(height^2^/R × 0.401) + (gender × 3.825) + (age × –0.071)] + 5.102, where height was measured in centimeters, resistance (R) in ohms, and sex coded as 1 for male and 0 for female. The SMI was then obtained as SMM divided by height squared (kg/m^2^). This study applied height‐adjusted rather than BMI‐adjusted SMM, as obesity status was a key variable in the analysis. PP was assessed by self‐reported gait speed in response to the question, “How would you describe your usual walking speed?,” with response options of “slow,” “average,” “brisk,” and “none of the above.” Participants reporting a “slow” walking speed were classified as having low PP.

### 4.4 Liver and Non‐Liver Disease Endpoints

Clinical outcomes were assessed through October 31, 2022, including cirrhosis, HCC, CVD (e.g., coronary artery disease, myocardial infarction, heart failure, cardiomyopathy, atrial fibrillation, stroke, and peripheral artery disease), CKD (e.g., chronic kidney disease, hypertensive chronic kidney disease, hypertensive heart and chronic kidney disease, dialysis, kidney transplantation, and kidney transplantation‐related complications), non‐liver cancers, obesity‐related cancers (defined according to the World Cancer Research Fund/International Agency for Research on Cancer classification) [42], and all‐cause mortality. Cases for all clinical outcomes were identified from linked hospital inpatient records and national cancer registry data using predefined International Classification of Diseases, Ninth Revision (ICD‐9), and Tenth Revision (ICD‐10) diagnostic codes [43], as detailed in Table S1.

### 4.5 Statistical Analyses

Participants were initially categorized into healthy controls (without MASLD or any sarcopenia‐related abnormalities) and individuals with MASLD. Among participants with MASLD, sarcopenia‐related characteristics were described according to obesity status. For each sarcopenia‐related abnormality, participants were subsequently classified into three mutually exclusive groups: healthy controls, MASLD without the corresponding sarcopenia‐related abnormality (MASLD alone), and MASLD with corresponding abnormalities. The coexistence group was further stratified into obesity and non‐obesity subgroups for subgroup analyses. Continuous variables are presented as mean (standard deviation, SD) and categorical variables as count (*n*) and percentage (%). Incidence rates were estimated as the number of new events divided by the accumulated person‐years (PYs) at risk and expressed per 10,000 PYs. Follow‐up time was accrued from baseline until the date of outcome occurrence, loss to follow‐up, death, or the end of follow‐up, whichever came first. Cox proportional hazards model was used to evaluate the association of sarcopenia/MASLD and the clinical outcomes. Overall and obesity‐stratified analyses were performed using two separate Cox regression models. Adjusted hazard ratios (aHRs) with 95% confidence intervals (CIs) were assessed through multivariable Cox regression analysis. We constructed three sequential models to evaluate the associations of interest. Model 1 was adjusted for age, sex, ethnicity, education level, and the Townsend deprivation index. Model 2 included all covariates in Model 1, with additional adjustments tailored to specific outcomes: the Charlson Comorbidity Index (CCI) for mortality, Fibrosis‐4 score (FIB‐4) for cirrhosis, HCC, non‐liver cancers, obesity‐related cancers, and FIB‐4 together with diabetes, hypertension, and hyperlipidemia for CKD and CVD. The stratification variable was omitted from the corresponding adjusted model. Model 3 further expanded upon Model 2 by incorporating laboratory variables, including alanine aminotransferase (ALT), albumin (ALB), alkaline phosphatase (ALP), C‐reactive protein (CRP), cholesterol, GGT, glycated hemoglobin (HbA1c), platelet count (PLT), high‐density lipoprotein cholesterol (HDL‐C), low‐density lipoprotein cholesterol (LDL‐C), and triglycerides. Covariates were selected based on clinical relevance and prior knowledge. All statistical analyses were two‐tailed and performed using R statistical software version 4.2.2 (R Foundation for Statistical Computing, Vienna, Austria).

### 4.6 Secondary Analyses

We performed subgroup analyses stratified by sex (male and female), age (younger vs. older, based on the median age [57 years]), FIB‐4 categories (<1.3, 1.3–2.67, and >2.67), and diabetes status (yes vs. no). Given that most UK Biobank participants were White, we performed a sensitivity analysis restricted to White participants to minimize bias due to population stratification. To further evaluate the external generalizability of our findings, we conducted an independent external validation using the United States National Health and Nutrition Examination Survey (NHANES) 2011–2018. We evaluated the associations of coexisting MASLD with low MM, low MS, and all‐cause mortality. Detailed methods, statistical analyses, and results are provided in the Supplementary Materials.

## Author Contributions

Mindie H. Nguyen and Hong Fan conceived and designed the study and contributed to data acquisition, analysis, and interpretation. Hong Fan performed the statistical analysis and drafted the manuscript. Ramsey Cheung and Mindie H. Nguyen provided administrative and material support for access to the UK Biobank data. Xianhua Mao, Taotao Yan, Scoot Barnett, Jing Zhang, Ramsey Cheung, Xuehong Zhang, and Mindie H. Nguyen critically reviewed and revised the manuscript for important intellectual content. Mindie H. Nguyen supervised the study, had full access to all study data, and takes responsibility for the integrity of the data and the accuracy of the data analysis. All authors have read and approved the final manuscript.

## Funding

There was no external funding. The study was supported by internal research funding at Stanford University Medical Center. The funder had no role in the study design, data collection, data analysis, interpretation of the results, preparation of the manuscript, or decision to submit the manuscript for publication.

## Ethics Statement

The UK Biobank received full ethical approval from the NHS National Research Ethics Service (16/NW/0274), and all participants provided written informed consent before data collection.

## Conflicts of Interest

Mindie H. Nguyen received research grants via Stanford University from Pfizer, Astra Zeneca, Glycotest, GSK, Delfi, Innogen, Exact Science, CurveBio, Gilead, Helio Health, National Institute of Health, Roche and personal fees from consulting/advisory board from Exelixis. All other authors have no competing interest to declare.

## Supporting information




**Supporting file 1**: mco271111‐sup‐0001‐SuppMat.pdf

## Data Availability

The data that support the findings of this study are derived from the UK Biobank (Application No. 31636) and the United States National Health and Nutrition Examination Survey (NHANES). UK Biobank data are not publicly available because they are subject to the UK Biobank's data access policies but are available to qualified researchers upon application and approval through the UK Biobank Access Management System. NHANES data are publicly available from the Centers for Disease Control and Prevention (CDC) website (https://wwwn.cdc.gov/nchs/nhanes/default.aspx).
