# Supplementary material for: Associations of Coexisting Metabolic Dysfunction‐Associated Steatotic Liver Disease and Sarcopenia‐Related Abnormalities With Adverse Clinical Outcomes Across Obesity States
Source: MedComm (2020). 2026 Oct 29;7(11):e71111. doi: 10.1002/mco2.71111 (PMC13718951; doi:10.1002/mco2.71111)
Supplement: Supplementary file 1 [file MCO2-7-e71111-s001.pdf]

Associations of Coexisting Metabolic Dysfunction-Associated Steatotic Liver Disease and Sarcopenia-Related Abnormalities With Adverse Clinical Outcomes Across Obesity States

Hong Fan[1], Xianhua Mao[1], Taotao Yan[1], Scott Barnett[1], Jing Zhang[2], Ramsey Cheung[1,3], Xuehong Zhang [4,5], Mindie H. Nguyen[1,6*].

1. Division of Gastroenterology and Hepatology, Stanford University Medical Center, Palo Alto, California, United States
2. The Third Unit, Department of Hepatology, Beijing Youan Hospital, Capital Medical University, Beijing, China
3. Division of Gastroenterology and Hepatology, Veterans Affairs Palo Alto Healthcare System, Palo Alto, California, United States
4. Department of Nutrition, Harvard T.H. Chan School of Public Health, Boston, MA, USA.
5. Yale University School of Nursing, Orange, CT, United States
6. Department of Epidemiology and Population Health, Stanford University Medical Center, Palo Alto, California, United States

Correspondence
Mindie H. Nguyen, MD, MAS, AGAF, FAASLD
Division of Gastroenterology and Hepatology,
Stanford University Medical Center,
Palo Alto, California, United States.
Email: mindiehn@stanford.edu

Table of Contents

Supplementary methods 5

Table S1. ICD-9/10-CM diagnosis codes and procedure codes 6

Table S2. Baseline characteristics of the study population according to categories of muscle strength (MS). 7

Table S3. Baseline characteristics of the study population according to categories of muscle mass (MM). 8

Table S4. Baseline characteristics of the study population according to categories of physical performance (PP). 9

Table S5. Association between combined MASLD and muscle strength (MS) status with clinical outcomes. 10

Table S6. Association between combined MASLD and muscle mass (MM) status with clinical outcomes. 12

Table S7. Association between combined MASLD and physical performance (PP) status with clinical outcomes. 14

Table S8. Incidence of clinical outcomes according to categories of MASLD and muscle strength (MS) stratified by age. 16

Table S9. Incidence of clinical outcomes according to categories of MASLD and muscle mass (MM) stratified by age. 18

Table S10. Incidence of clinical outcomes according to categories of MASLD and physical performance (PP) stratified by age. 20

Table S11. Incidence of clinical outcomes according to categories of MASLD and muscle strength (MS) stratified by sex. 22

Table S12. Incidence of clinical outcomes according to categories of MASLD and muscle mass (MM) stratified by sex. 24

Table S13. Incidence of clinical outcomes according to categories of MASLD and physical performance (PP) stratified by sex. 26

Table S14. Incidence of clinical outcomes according to categories of MASLD and muscle strength (MS) stratified by diabetes. 28

Table S15. Incidence of clinical outcomes according to categories of MASLD and muscle mass (MM) stratified by diabetes. 30

Table S16. Incidence of clinical outcomes according to categories of MASLD and physical performance (PP) stratified by diabetes. 32

Table S17. Incidence of clinical outcomes according to categories of MASLD and muscle strength (MS) stratified by FIB-4. 34

Table S18. Incidence of clinical outcomes according to categories of MASLD and muscle mass (MM) stratified by FIB-4. 37

Table S19. Incidence of clinical outcomes according to categories of MASLD and muscle strength (MS) stratified by FIB-4. 40

Table S20. Incidence of clinical outcomes according to MASLD and muscle strength (MS) categories in non-White participants (sensitivity analysis) 43

Table S21. Incidence of clinical outcomes according to MASLD and muscle mass (MM) categories in non-White participants (sensitivity analysis). 44

Table S22. Incidence of clinical outcomes according to MASLD and physical performance (PP) categories in non-White participants (sensitivity analysis). 45

Table S23. Association between combined MASLD and muscle strength (MS) status and clinical outcomes, stratified by age. 46

Table S24. Association between combined MASLD and muscle mass (MM) status and clinical outcomes, stratified by age 49

Table S25. Association between combined MASLD and physical performance (PP) status and clinical outcomes, stratified by age. 52

Table S26. Association between combined MASLD and muscle strength (MS) status and clinical outcomes, stratified by sex. 55

Table S27. Association between combined MASLD and muscle mass (MM) status and clinical outcomes, stratified by sex 58

Table S28. Association between combined MASLD and physical performance (PP) status and clinical outcomes, stratified by sex. 61

Table S29. Association between combined MASLD and muscle strength (MS) status and clinical outcomes, stratified by diabetes. 64

Table S30. Association between combined MASLD and muscle mass (MM) status and clinical outcomes, stratified by diabetes. 67

Table S31. Association between combined MASLD and physical performance (PP) status and clinical outcomes, stratified by diabetes. 70

Table S32. Association between combined MASLD and muscle strength (MS) status and clinical outcomes, stratified by FIB-4. 73

Table S33. Association between combined MASLD and muscle mass (MM) status and clinical outcomes, stratified by FIB-4 77

Table S34. Association between combined MASLD and physical performance (PP) status and clinical outcomes, stratified by FIB-4. 81

Table S35. Sensitivity analysis of the association between combined MASLD and muscle strength (MS) status and clinical outcomes, restricted to White participants. 85

Table S36. Sensitivity analysis of the association between combined MASLD and muscle mass (MM) status and clinical outcomes, restricted to White participants 87

Table S37. Sensitivity analysis of the association between combined MASLD and physical performance (PP) status and clinical outcomes, restricted to White participants 89

Table S38. Long-term mortality rates according to categories of MASLD and sarcopenia-related abnormalities. 91

Table S39. Association between combined MASLD and sarcopenia-related abnormalities status with death. 92

Supplementary methods

External validation using NHANES

To further evaluate the external generalizability of our findings, we conducted an external validation using data from the National Health and Nutrition Examination Survey (NHANES) 2011–2018, a nationally representative survey of the noninstitutionalized U.S. population conducted by the National Center for Health Statistics (NCHS). Four consecutive survey cycles (2011–2012, 2013–2014, 2015–2016, and 2017–2018) were included. Participants with missing data required for exposure definition or mortality follow-up were excluded.

Exposure definition

The same analytical framework and hierarchical exposure classification used in the UK Biobank analysis were applied whenever possible to facilitate comparison between the two cohorts. Hepatic steatosis was identified using the Fatty Liver Index (FLI), and participants with FLI >60 were considered to have hepatic steatosis. MASLD was defined according to the international consensus criteria. Low muscle mass was assessed using appendicular skeletal muscle mass measured by dual-energy X-ray absorptiometry (DXA), and low muscle strength was defined using handgrip strength according to the same sex-specific cutoffs used in the primary UK Biobank analysis. Participants were classified into three mutually exclusive groups: Healthy (without MASLD or sarcopenia-related abnormalities), (2) MASLD alone, and (3) MASLD with low muscle mass or low muscle strength. The coexistence group was further stratified by obesity status into obesity and non-obesity subgroups.

Outcome

The outcome for external validation was all-cause mortality, identified through linkage with the National Death Index. Follow-up time was calculated from the Mobile Examination Center (MEC) examination date to death or the end of follow-up.

Statistical analysis

Cox proportional hazards models were used to estimate hazard ratios (HRs) and 95% confidence intervals (CIs) for all-cause mortality according to the exposure groups. Three adjustment models were applied. Model 1 was adjusted for age, sex, race/ethnicity, education level, and poverty-income ratio. Model 2 further adjusted for the fibrosis-4 (FIB-4) score. Model 3 additionally adjusted for alanine aminotransferase (ALT), albumin, alkaline phosphatase (ALP), total cholesterol, gamma-glutamyl transferase (GGT), glycated hemoglobin (HbA1c), platelet count, high-density lipoprotein cholesterol (HDL-C), low-density lipoprotein cholesterol (LDL-C), and triglycerides.

Table S1. ICD-9/10-CM diagnosis codes and procedure codes

Disease	ICD-9/10-CM diagnosis codes/procedure codes
Hepatitis B virus	070.22, 070.23, 070.32, 070.33; B18.0, B18.1
Hepatitis C virus	070.41, 070.44, 070.51, 070.54, 070.70, 070.71, V02.62; B17.10, B18.2, B19.2
Liver cirrhosis	571.2, 571.5, 572.3, 456.1, 456.21; K70.3, K74.6, K76.6, I85.0, I85.9
Hepatocellular carcinoma	155.0; C22.0, C22.8
Human immunodeficiency virus	042, 079.53, V08; B20–B24, Z21
Cardiovascular diseases	398.91; 402.01, 402.11, 402.91; 404.01, 404.03, 404.11, 404.13, 404.91, 404.93; 410.00–414.9; 425.4–425.9; 427.31, 427.32; 428.0, 428.1, 428.20–428.23, 428.30–428.33, 428.40–428.43, 428.9; 430–438; 440.20–440.29, 443.9, 444.0–444.9; I20.9, I21.0–I21.9, I21.A1, I21.A9; I25, I25.1, I25.10, I25.11X, I25.2, I25.5, I25.84, I25.9; I42.0–I42.9; I43; I48.0–I48.9; I50.1–I50.4, I50.8, I50.9; I60.0–I69.9; I70.2–I70.9, I73.9, I74.0–I74.9
Chronic kidney disease	585.1–585.6, 585.9; 403.00, 403.01, 403.10, 403.11, 403.90, 403.91; 404.00–404.03, 404.10–404.13, 404.90–404.93; V42.0, 996.81, 55.69*; N18.1–N18.6, N18.9, I12.0, I12.9, I13.0, I13.1, I13.2, Z94.0, Z49, Z99.2, T86.1
Extrahepatic cancers	14X.X–208.X (excluding 173.X and 155.0); C00–C97 (excluding C43.X, C44.X and C22.0, C22.8)
Obesity–related cancers	150.0–150.9, 151.0, 153.0–153.9, 154.0, 154.1, 154.8, 155.0–155.2, 156.0, 157.0–157.9, 174.0–174.9, 182.0, 183.0, 189.0, 193, 203.0; C15.0–C15.9, C16.0, C18.0–C18.9, C19, C20, C22.0–C22.9, C23, C25.0–C25.9, C50.0–C50.9, C54.0–C54.9, C56, C64, C73, C90.0
Diabetes Mellitus	250.0X–250.9X; E10.X–E11.X, E13.X
Hypertension	401, 401.0, 401.1, 401.9; I10
Hyperlipidemia	272.0–272.4; E78.00, E78.1–E78.4, E78.49, E78.5
Obesity	278.00, 278.01, 278.03; V85.30–V85.39, V85.41–V85.45; E66.0, E66.01, E66.09, E66.1, E66.2, E66.8, E66.9; Z68.30–Z68.39; Z68.41–Z68.45

Table S2. Baseline characteristics of the study population according to categories of muscle strength (MS).

	Healthy (n=274430)	MASLD+ normal MS (n=154819)	MASLD+ low MS (n=9499)	MASLD+ low MS-NO (n=3494)	MASLD+ low MS-O (n=6005)
Age (year)	55.66 (8.21)	57.01 (7.85)	59.85 (7.18)	60.03 (7.34)	59.74 (7.09)
Male	100326 (36.6)	97498 (63.0)	4636 (48.8)	2230 (63.8)	2406 (40.1)
White ethnicity	260335 (94.9)	146143 (94.4)	8402 (88.5)	3020 (86.4)	5382 (89.6)
Townsend deprivation					
Q1	71998 (26.3)	35852 (23.2)	1494 (15.8)	604 (17.3)	890 (14.9)
Q2-Q3	139259 (50.8)	75708 (49.0)	4112 (43.4)	1563 (44.9)	2549 (42.5)
Q4	62694 (22.9)	42990 (27.8)	3869 (40.8)	1317 (37.8)	2552 (42.6)
Education levels					
High	114047 (42.0)	50658 (32.7)	2255 (23.8)	959 (27.5)	1296 (21.6)
Low	119402 (44.0)	70869 (45.8)	3695 (38.9)	1326 (38.0)	2369 (39.5)
Others	38000 (14.0)	33172 (21.4)	3544 (37.3)	1204 (34.5)	2340 (39.0)
BMI (kg/m^2)	25.17 (3.08)	31.36 (4.47)	32.17 (4.87)	27.71 (1.70)	34.76 (4.19)
Diabetes	9919 (3.6)	25116 (16.2)	2751 (29.0)	748 (21.4)	2003 (33.4)
Hyperlipidemia	4911 (1.8)	6626 (4.3)	665 (7.0)	217 (6.2)	448 (7.5)
Hypertension	61933 (22.6)	68348 (44.1)	5762 (60.7)	1823 (52.2)	3939 (65.6)
FIB4	1.34 (1.38)	1.31 (2.66)	1.41 (3.10)	1.40 (0.83)	1.41 (3.84)
FIB4>2.67	4741 (2.0)	3339 (2.2)	322 (3.5)	122 (3.6)	200 (3.5)
CCI score	1.02 (2.09)	1.68 (2.62)	2.71 (3.20)	2.40 (3.10)	2.90 (3.24)
Age adjusted CCI	2.14 (2.39)	2.93 (2.91)	4.22 (3.41)	3.92 (3.32)	4.39 (3.44)
ALT (U/L)	19.45 (9.70)	29.96 (17.14)	28.30 (17.18)	29.55 (18.94)	27.58 (16.03)
AST (U/L)	24.57 (8.20)	28.59 (12.61)	28.37 (13.56)	29.21 (16.04)	27.88 (11.85)
Albumin (g/L)	45.32 (2.58)	45.15 (2.63)	44.42 (2.79)	44.95 (2.79)	44.12 (2.74)
ALP (U/L)	79.54 (23.48)	88.45 (28.26)	94.09 (31.46)	95.06 (37.21)	93.53 (27.57)
CRP (mg/L)	1.83 (3.59)	3.52 (4.71)	4.86 (6.30)	3.78 (5.35)	5.49 (6.72)
Cholesterol (mmol/L)	5.68 (1.08)	5.72 (1.22)	5.52 (1.30)	5.69 (1.34)	5.41 (1.26)
GGT (U/L)	25.72 (18.79)	53.31 (55.24)	56.95 (66.99)	66.16 (82.02)	51.61 (55.78)
Glucose (mmol/L)	4.97 (0.89)	5.32 (1.56)	5.65 (2.03)	5.48 (1.84)	5.74 (2.13)
HbA1c (mmol/mol)	34.89 (5.05)	37.81 (8.25)	40.21 (10.26)	38.83 (9.42)	41.02 (10.64)
HDL-C (mmol/L)	1.57 (0.38)	1.25 (0.29)	1.25 (0.31)	1.24 (0.31)	1.25 (0.31)
LDL-C (mmol/L)	3.51 (0.82)	3.65 (0.91)	3.47 (0.97)	3.59 (0.99)	3.40 (0.95)
Triglycerides (mmol/L)	1.33 (0.61)	2.39 (1.19)	2.38 (1.21)	2.61 (1.29)	2.25 (1.15)
PLT (10^9 cells/Litre)	252.75 (58.56)	253.14 (60.80)	259.21 (66.30)	256.18 (66.39)	260.98 (66.19)

Table S3. Baseline characteristics of the study population according to categories of muscle mass (MM).

	Healthy (n=274430)	MASLD+ normal SMI (n=163675)	MASLD+ low SMI (n=643)	MASLD+ low SMI-NO (n=525)	MASLD+ low SMI-O (n=118)
Age (year)	55.66 (8.21)	57.16 (7.84)	62.24 (5.56)	62.13 (5.55)	62.73 (5.62)
Male	100326 (36.6)	102073 (62.4)	61 (9.5)	58 (11.0)	3 (2.5)
White ethnicity	260335 (94.9)	153957 (94.1)	588 (91.4)	480 (91.4)	108 (91.5)
Townsend deprivation					
Q1	71998 (26.3)	37205 (22.8)	141 (22.0)	123 (23.5)	18 (15.3)
Q2-Q3	139259 (50.8)	79507 (48.7)	313 (48.8)	252 (48.1)	61 (51.7)
Q4	62694 (22.9)	46671 (28.6)	188 (29.3)	149 (28.4)	39 (33.1)
Education levels					
High	114047 (42.0)	52744 (32.2)	169 (26.4)	141 (27.0)	28 (23.7)
Low	119402 (44.0)	74316 (45.4)	248 (38.8)	211 (40.4)	37 (31.4)
Others	38000 (14.0)	36493 (22.3)	223 (34.8)	170 (32.6)	53 (44.9)
BMI (kg/m^2)	25.17 (3.08)	31.42 (4.49)	27.34 (3.49)	26.16 (2.32)	32.59 (2.91)
Diabetes	9919 (3.6)	27783 (17.0)	84 (13.1)	65 (12.4)	19 (16.1)
Hyperlipidemia	4911 (1.8)	7263 (4.4)	28 (4.4)	24 (4.6)	4 (3.4)
Hypertension	61933 (22.6)	73789 (45.1)	321 (49.9)	253 (48.2)	68 (57.6)
FIB4	1.34 (1.38)	1.32 (2.69)	1.38 (0.82)	1.39 (0.85)	1.32 (0.68)
FIB4>2.67	4741 (2.0)	3636 (2.3)	25 (4.1)	20 (4.0)	5 (4.5)
CCI score	1.02 (2.09)	1.74 (2.67)	2.24 (2.96)	2.21 (2.95)	2.40 (2.98)
Age adjusted CCI	2.14 (2.39)	3.00 (2.96)	3.95 (3.08)	3.90 (3.08)	4.15 (3.09)
ALT (U/L)	19.45 (9.70)	29.86 (17.11)	30.10 (23.91)	31.76 (25.75)	22.79 (10.38)
AST (U/L)	24.57 (8.20)	28.56 (12.61)	31.16 (22.85)	32.58 (24.88)	24.82 (6.57)
Albumin (g/L)	45.32 (2.58)	45.11 (2.64)	45.08 (2.81)	45.33 (2.81)	43.97 (2.57)
ALP (U/L)	79.54 (23.48)	88.70 (28.19)	108.89 (68.59)	112.14 (74.84)	94.65 (23.09)
CRP (mg/L)	1.83 (3.59)	3.60 (4.82)	4.87 (5.96)	4.87 (6.08)	4.86 (5.48)
Cholesterol (mmol/L)	5.68 (1.08)	5.71 (1.22)	6.33 (1.34)	6.40 (1.35)	6.02 (1.27)
GGT (U/L)	25.72 (18.79)	53.39 (55.60)	85.39 (116.11)	94.38 (125.17)	45.94 (44.88)
Glucose (mmol/L)	4.97 (0.89)	5.34 (1.60)	5.26 (1.29)	5.25 (1.27)	5.31 (1.36)
HbA1c (mmol/mol)	34.89 (5.05)	37.95 (8.40)	37.61 (7.47)	37.46 (7.03)	38.27 (9.14)
HDL-C (mmol/L)	1.57 (0.38)	1.25 (0.29)	1.44 (0.35)	1.44 (0.36)	1.46 (0.28)
LDL-C (mmol/L)	3.51 (0.82)	3.63 (0.92)	3.99 (1.01)	4.04 (1.01)	3.79 (0.98)
Triglycerides (mmol/L)	1.33 (0.61)	2.39 (1.20)	2.54 (1.18)	2.66 (1.21)	2.04 (0.93)
PLT (10^9 cells/Litre)	252.75 (58.56)	253.39 (61.09)	280.91 (69.95)	280.47 (69.52)	282.86 (72.13)

Table S4. Baseline characteristics of the study population according to categories of physical performance (PP).

	Healthy (n=274430)	MASLD+ normal PP (n=142149)	MASLD+ low PP (n=22169)	MASLD+ low PP-NO (n=5613)	MASLD+ low PP-O (n=16556)
Age (year)	55.66 (8.21)	56.87 (7.87)	59.15 (7.30)	59.64 (7.25)	58.98 (7.31)
Male	100326 (36.6)	91335 (64.3)	10799 (48.7)	3872 (69.0)	6927 (41.8)
White ethnicity	260335 (94.9)	134474 (94.6)	20071 (90.5)	4976 (88.7)	15095 (91.2)
Townsend deprivation					
Q1	71998 (26.3)	34026 (24.0)	3320 (15.0)	884 (15.8)	2436 (14.7)
Q2-Q3	139259 (50.8)	70493 (49.7)	9327 (42.2)	2366 (42.2)	6961 (42.1)
Q4	62694 (22.9)	37379 (26.3)	9480 (42.8)	2354 (42.0)	7126 (43.1)
Education levels					
High	114047 (42.0)	47869 (33.7)	5044 (22.8)	1290 (23.0)	3754 (22.7)
Low	119402 (44.0)	65917 (46.4)	8647 (39.0)	2061 (36.7)	6586 (39.8)
Others	38000 (14.0)	28242 (19.9)	8474 (38.2)	2262 (40.3)	6212 (37.5)
BMI (kg/m^2)	25.17 (3.08)	30.98 (4.09)	34.14 (5.83)	27.89 (1.62)	36.26 (5.18)
Diabetes	9919 (3.6)	20685 (14.6)	7182 (32.4)	1335 (23.8)	5847 (35.3)
Hyperlipidemia	4911 (1.8)	5591 (3.9)	1700 (7.7)	427 (7.6)	1273 (7.7)
Hypertension	61933 (22.6)	59791 (42.1)	14319 (64.6)	3310 (59.0)	11009 (66.5)
FIB4	1.34 (1.38)	1.31 (2.67)	1.37 (2.79)	1.40 (0.81)	1.36 (3.20)
FIB4>2.67	4741 (2.0)	2988 (2.2)	673 (3.1)	213 (3.9)	460 (2.9)
CCI score	1.02 (2.09)	1.55 (2.51)	2.98 (3.25)	2.98 (3.30)	2.98 (3.24)
Age adjusted CCI	2.14 (2.39)	2.79 (2.80)	4.42 (3.49)	4.47 (3.54)	4.40 (3.47)
ALT (U/L)	19.45 (9.70)	30.13 (17.17)	28.16 (16.90)	29.28 (18.66)	27.78 (16.24)
AST (U/L)	24.57 (8.20)	28.64 (12.60)	28.14 (13.06)	29.38 (15.37)	27.72 (12.16)
Albumin (g/L)	45.32 (2.58)	45.23 (2.60)	44.35 (2.77)	45.11 (2.82)	44.10 (2.70)
ALP (U/L)	79.54 (23.48)	87.87 (27.67)	94.58 (32.65)	96.53 (41.54)	93.92 (29.00)
CRP (mg/L)	1.83 (3.59)	3.32 (4.44)	5.42 (6.52)	4.19 (5.95)	5.83 (6.64)
Cholesterol (mmol/L)	5.68 (1.08)	5.76 (1.21)	5.41 (1.29)	5.62 (1.34)	5.34 (1.26)
GGT (U/L)	25.72 (18.79)	52.74 (53.41)	58.47 (70.08)	71.55 (93.24)	54.05 (59.62)
Glucose (mmol/L)	4.97 (0.89)	5.29 (1.49)	5.69 (2.13)	5.50 (2.00)	5.75 (2.17)
HbA1c (mmol/mol)	34.89 (5.05)	37.50 (7.85)	40.84 (10.89)	39.16 (10.15)	41.40 (11.07)
HDL-C (mmol/L)	1.57 (0.38)	1.25 (0.29)	1.23 (0.31)	1.23 (0.32)	1.23 (0.31)
LDL-C (mmol/L)	3.51 (0.82)	3.67 (0.91)	3.40 (0.96)	3.54 (1.00)	3.36 (0.95)
Triglycerides (mmol/L)	1.33 (0.61)	2.39 (1.19)	2.35 (1.22)	2.58 (1.28)	2.27 (1.19)
PLT (10^9 cells/Litre)	252.75 (58.56)	252.37 (60.06)	260.68 (67.32)	258.16 (67.21)	261.54 (67.34)

Table S5. Association between combined MASLD and muscle strength (MS) status with clinical outcomes.

		Model1		Model2		Model3	
**Outcomes**	**Groups**	**HR (95% CI)**	**P value**	**HR (95% CI)**	**P value**	**HR (95% CI)**	**P value**
Death	Healthy						
	MASLD+normal MS	1.36 (1.33, 1.40)	<0.001	1.01 (0.98, 1.03)	0.510	1.03 (1.00, 1.06)	0.062
	MASLD+low MS-NO	1.88 (1.73, 2.04)	<0.001	1.16 (1.07, 1.25)	<0.001	1.16 (1.06, 1.27)	0.001
	MASLD+low MS-O	2.12 (1.99, 2.25)	<0.001	1.09 (1.02, 1.16)	0.007	1.07 (1.00, 1.15)	0.047
	MASLD+low MS	2.02 (1.93, 2.13)	<0.001	1.11 (1.06, 1.17)	<0.001	1.10 (1.04, 1.17)	0.001
Cirrhosis	Healthy						
	MASLD+normal MS	3.40 (3.03, 3.81)	<0.001	3.93 (3.47, 4.46)	<0.001	3.45 (2.95, 4.02)	<0.001
	MASLD+low MS-NO	4.91 (3.57, 6.77)	<0.001	5.57 (4.00, 7.76)	<0.001	3.03 (2.03, 4.52)	<0.001
	MASLD+low MS-O	7.38 (5.93, 9.19)	<0.001	8.48 (6.73, 10.68)	<0.001	6.16 (4.72, 8.03)	<0.001
	MASLD+low MS	6.44 (5.31, 7.81)	<0.001	7.36 (6.00, 9.02)	<0.001	4.90 (3.84, 6.25)	<0.001
HCC	Healthy						
	MASLD+normal MS	2.72 (2.17, 3.42)	<0.001	2.77 (2.17, 3.54)	<0.001	1.78 (1.33, 2.38)	<0.001
	MASLD+low MS-NO	4.79 (2.68, 8.56)	<0.001	4.73 (2.58, 8.68)	<0.001	2.12 (1.06, 4.25)	0.034
	MASLD+low MS-O	5.56 (3.46, 8.92)	<0.001	5.69 (3.49, 9.28)	<0.001	3.13 (1.84, 5.33)	<0.001
	MASLD+low MS	5.24 (3.54, 7.75)	<0.001	5.29 (3.51, 7.96)	<0.001	2.71 (1.70, 4.33)	<0.001
Non-liver cancers	Healthy						
	MASLD+normal MS	1.15 (1.12, 1.17)	<0.001	1.15 (1.12, 1.18)	<0.001	1.11 (1.08, 1.14)	<0.001
	MASLD+low MS-NO	1.01 (0.92, 1.12)	0.785	1.03 (0.93, 1.14)	0.621	0.94 (0.84, 1.05)	0.289
	MASLD+low MS-O	1.24 (1.15, 1.34)	<0.001	1.24 (1.15, 1.34)	<0.001	1.14 (1.05, 1.24)	0.002
	MASLD+low MS	1.15 (1.08, 1.22)	<0.001	1.16 (1.08, 1.23)	<0.001	1.06 (0.99, 1.14)	0.094
Obesity-related cancers	Healthy						
	MASLD+normal MS	1.39 (1.34, 1.45)	<0.001	1.41 (1.36, 1.47)	< 0.001	1.27 (1.21, 1.34)	<0.001
	MASLD+low MS-NO	1.08 (0.90, 1.31)	0.391	1.10 (0.91, 1.33)	0.323	0.94 (0.76, 1.15)	0.532

	MASLD+low MS-O	1.51 (1.34, 1.71)	<0.001	1.54 (1.36, 1.75)	<0.001	1.30 (1.13, 1.49)	<0.001
	MASLD+low MS	1.35 (1.22, 1.50)	<0.001	1.38 (1.24, 1.53)	<0.001	1.16 (1.03, 1.31)	0.012
CVD	Healthy						
	MASLD+normal MS	1.51 (1.48, 1.54)	<0.001	1.07 (1.05, 1.10)	<0.001	1.00 (0.97, 1.03)	0.932
	MASLD+low MS-NO	1.80 (1.66, 1.95)	<0.001	1.28 (1.18, 1.39)	<0.001	1.17 (1.07, 1.28)	<0.001
	MASLD+low MS-O	2.30 (2.17, 2.44)	<0.001	1.33 (1.25, 1.41)	<0.001	1.20 (1.12, 1.29)	<0.001
	MASLD+low MS	2.10 (2.00, 2.21)	<0.001	1.31 (1.25, 1.38)	<0.001	1.19 (1.13, 1.26)	<0.001
CKD	Healthy						
	MASLD+normal MS	2.00 (1.94, 2.07)	<0.001	1.32 (1.27, 1.36)	<0.001	1.20 (1.15, 1.25)	<0.001
	MASLD+low MS-NO	2.35 (2.11, 2.61)	<0.001	1.51 (1.35, 1.68)	<0.001	1.35 (1.20, 1.52)	<0.001
	MASLD+low MS-O	3.44 (3.21, 3.69)	<0.001	1.76 (1.64, 1.90)	<0.001	1.44 (1.32, 1.56)	<0.001
	MASLD+low MS	3.02 (2.84, 3.21)	<0.001	1.68 (1.57, 1.79)	<0.001	1.41 (1.31, 1.51)	<0.001

Table S6. Association between combined MASLD and muscle mass (MM) status with clinical outcomes.

		Model1		Model2a		Model3a	
**Outcomes**	**Groups**	**HR (95% CI)**	**P value**	**HR (95% CI)**	**P value**	**HR (95% CI)**	**P value**
Death	Healthy						
	MASLD+normal MM	1.41 (1.38, 1.44)	<0.001	1.01 (0.99, 1.04)	0.204	1.03 (1.00, 1.06)	0.033
	MASLD+low MM-NO	1.88 (1.53, 2.33)	<0.001	1.20 (0.97, 1.48)	0.089	1.24 (0.98, 1.56)	0.070
	MASLD+low MM-O	1.93 (1.27, 2.93)	0.002	1.61 (1.06, 2.45)	0.025	1.43 (0.89, 2.30)	0.140
	MASLD+low MM	1.89 (1.57, 2.29)	<0.001	1.27 (1.05, 1.53)	0.014	1.27 (1.03, 1.56)	0.024
Cirrhosis	Healthy						
	MASLD+normal MM	3.59 (3.21, 4.02)	<0.001	4.15 (3.67, 4.70)	<0.001	3.55 (3.04, 4.14)	<0.001
	MASLD+low MM-NO	4.01 (1.50, 10.75)	0.006	3.39 (1.09, 10.58)	0.035	1.16 (0.29, 4.69)	0.838
	MASLD+low MM-O	4.14 (0.58, 29.51)	0.156	5.09 (0.71, 36.28)	0.104	—	—
	MASLD+low MM	4.04 (1.67, 9.76)	0.002	3.70 (1.38, 9.93)	0.009	1.06 (0.26, 4.29)	0.935
HCC	Healthy						
	MASLD+normal MM	2.87 (2.29, 3.60)	<0.001	2.92 (2.29, 3.72)	<0.001	1.82 (1.36, 2.43)	<0.001
	MASLD+low MM-NO	8.19 (2.01, 33.33)	0.003	8.00 (1.96, 32.61)	0.004	3.24 (0.77, 13.59)	0.108
	MASLD+low MM-O	—	—	—	—	—	—
	MASLD+low MM	6.67 (1.64, 27.16)	0.008	6.59 (1.62, 26.90)	0.009	2.98 (0.71, 12.47)	0.135
Non-liver cancers	Healthy						
	MASLD+normal MM	1.15 (1.12, 1.17)	<0.001	1.15 (1.12, 1.17)	<0.001	1.11 (1.08, 1.14)	<0.001
	MASLD+low MM-NO	1.24 (0.96, 1.61)	0.104	1.28 (0.99, 1.66)	0.061	1.04 (0.77, 1.41)	0.783
	MASLD+low MM-O	1.17 (0.67, 2.06)	0.582	1.27 (0.72, 2.23)	0.413	1.21 (0.65, 2.26)	0.542
	MASLD+low MM	1.23 (0.97, 1.56)	0.088	1.28 (1.01, 1.62)	0.041	1.07 (0.82, 1.41)	0.617
Obesity-related cancers	Healthy						
	MASLD+normal MM	1.39 (1.34, 1.44)	<0.001	1.41 (1.36, 1.46)	<0.001	1.27 (1.21, 1.33)	<0.001
	MASLD+low MM-NO	1.36 (0.91, 2.03)	0.135	1.41 (0.94, 2.11)	0.092	1.16 (0.75, 1.81)	0.499
	MASLD+low MM-O	1.44 (0.65, 3.22)	0.369	1.56 (0.70, 3.47)	0.277	1.44 (0.60, 3.47)	0.413

	MASLD+low MM	1.37 (0.96, 1.97)	0.083	1.44 (1.00, 2.06)	0.047	1.21 (0.82, 1.80)	0.342
CVD	Healthy						
	MASLD+normal MM	1.55 (1.52, 1.58)	<0.001	1.09 (1.06, 1.11)	<0.001	1.01 (0.99, 1.04)	0.338
	MASLD+low MM-NO	1.40 (1.09, 1.81)	0.008	1.09 (0.84, 1.40)	0.522	1.00 (0.76, 1.32)	0.976
	MASLD+low MM-O	1.52 (0.93, 2.48)	0.094	0.92 (0.52, 1.62)	0.764	0.89 (0.49, 1.61)	0.699
	MASLD+low MM	1.43 (1.14, 1.79)	0.002	1.05 (0.84, 1.33)	0.658	0.98 (0.76, 1.26)	0.886
CKD	Healthy						
	MASLD+normal MM	2.08 (2.02, 2.15)	<0.001	1.35 (1.30, 1.39)	<0.001	1.22 (1.17, 1.27)	<0.001
	MASLD+low MM-NO	1.69 (1.27, 2.26)	<0.001	1.25 (0.93, 1.69)	0.140	1.01 (0.71, 1.43)	0.976
	MASLD+low MM-O	0.69 (0.29, 1.66)	0.410	0.52 (0.22, 1.25)	0.146	0.36 (0.11, 1.10)	0.073
	MASLD+low MM	1.48 (1.13, 1.95)	0.005	1.09 (0.82, 1.45)	0.536	0.87 (0.62, 1.21)	0.408

Table S7. Association between combined MASLD and physical performance (PP) status with clinical outcomes.

		Model1		Model2a		Model3a	
**Outcomes**	**Groups**	**HR (95% CI)**	**P value**	**HR (95% CI)**	**P value**	**HR (95% CI)**	**P value**
Death	Healthy						
	MASLD+normal PP	1.23 (1.20, 1.25)	<0.001	0.97 (0.95, 1.00)	<0.001	1.00 (0.97, 1.03)	0.856
	MASLD+low PP-NO	2.56 (2.42, 2.70)	<0.001	1.30 (1.22, 1.37)	<0.001	1.27 (1.19, 1.35)	<0.001
	MASLD+low PP-O	2.45 (2.36, 2.54)	<0.001	1.15 (1.11, 1.20)	<0.001	1.18 (1.12, 1.23)	<0.001
	MASLD+low PP	2.48 (2.40, 2.56)	<0.001	1.19 (1.15, 1.23)	<0.001	1.20 (1.15, 1.25)	<0.001
Cirrhosis	Healthy						
	MASLD+normal PP	3.05 (2.72, 3.43)	<0.001	3.54 (3.12, 4.03)	<0.001	3.19 (2.72, 3.74)	<0.001
	MASLD+low PP-NO	5.46 (4.28, 6.96)	<0.001	5.82 (4.53, 7.49)	<0.001	3.11 (2.31, 4.19)	<0.001
	MASLD+low PP-O	7.22 (6.17, 8.44)	<0.001	8.24 (6.98, 9.73)	<0.001	6.11 (5.01, 7.45)	<0.001
	MASLD+low PP	6.73 (5.82, 7.79)	<0.001	7.55 (6.45, 8.83)	<0.001	5.16 (4.26, 6.25)	<0.001
HCC	Healthy						
	MASLD+normal PP	2.50 (1.98, 3.17)	<0.001	2.57 (2.00, 3.31)	<0.001	1.73 (1.29, 2.33)	<0.001
	MASLD+low PP-NO	5.42 (3.47, 8.44)	<0.001	5.21 (3.29, 8.27)	<0.001	2.16 (1.26, 3.70)	0.005
	MASLD+low PP-O	5.06 (3.60, 7.10)	<0.001	4.94 (3.46, 7.06)	<0.001	2.38 (1.58, 3.60)	<0.001
	MASLD+low PP	5.17 (3.82, 6.99)	<0.001	5.03 (3.66, 6.92)	<0.001	2.31 (1.58, 3.38)	<0.001
Non-liver cancers	Healthy						
	MASLD+normal PP	1.13 (1.11, 1.16)	<0.001	1.14 (1.11, 1.17)	<0.001	1.11 (1.07, 1.14)	<0.001
	MASLD+low PP-NO	1.22 (1.13, 1.31)	<0.001	1.17 (1.09, 1.27)	<0.001	1.11 (1.02, 1.21)	0.012
	MASLD+low PP-O	1.24 (1.18, 1.30)	<0.001	1.23 (1.18, 1.30)	<0.001	1.14 (1.08, 1.21)	<0.001
	MASLD+low PP	1.24 (1.18, 1.29)	<0.001	1.22 (1.17, 1.27)	<0.001	1.13 (1.08, 1.19)	<0.001
Obesity-related cancers	Healthy						
	MASLD+normal PP	1.36 (1.31, 1.41)	<0.001	1.38 (1.33, 1.44)	<0.001	1.26 (1.20, 1.33)	<0.001

	MASLD+low PP-NO	1.42 (1.24, 1.61)	<0.001	1.38 (1.21, 1.58)	<0.001	1.20 (1.03, 1.39)	0.018
	MASLD+low PP-O	1.61 (1.50, 1.74)	<0.001	1.62 (1.50, 1.75)	<0.001	1.35 (1.24, 1.48)	<0.001
	MASLD+low PP	1.56 (1.46, 1.67)	<0.001	1.56 (1.45, 1.67)	<0.001	1.31 (1.21, 1.43)	<0.001
CVD	Healthy						
	MASLD+normal PP	1.39 (1.36, 1.42)	<0.001	1.03 (1.00, 1.05)	0.028	0.97 (0.94, 1.00)	0.022
	MASLD+low PP-NO	2.42 (2.28, 2.56)	<0.001	1.50 (1.41, 1.59)	<0.001	1.38 (1.30, 1.48)	<0.001
	MASLD+low PP-O	2.64 (2.54, 2.74)	<0.001	1.43 (1.38, 1.49)	<0.001	1.27 (1.22, 1.33)	<0.001
	MASLD+low PP	2.58 (2.49, 2.66)	<0.001	1.45 (1.40, 1.50)	<0.001	1.30 (1.25, 1.36)	<0.001
CKD	Healthy						
	MASLD+normal PP	1.79 (1.74, 1.85)	<0.001	1.24 (1.20, 1.29)	<0.001	1.15 (1.10, 1.20)	<0.001
	MASLD+low PP-NO	2.76 (2.55, 2.99)	<0.001	1.63 (1.50, 1.76)	<0.001	1.38 (1.26, 1.52)	<0.001
	MASLD+low PP-O	3.90 (3.72, 4.08)	<0.001	1.89 (1.80, 1.99)	<0.001	1.57 (1.48, 1.66)	<0.001
	MASLD+low PP	3.59 (3.44, 3.75)	<0.001	1.83 (1.75, 1.92)	<0.001	1.53 (1.45, 1.61)	<0.001

Table S8. Incidence of clinical outcomes according to categories of MASLD and muscle strength (MS) stratified by age.

Outcomes		Groups	Incidence		
			Incident cases	PYs	Incidence (10,000 PYs)
Death	Younger	Healthy	3971	2002063	19.83 (19.22, 20.46)
		MASLD+normal MS	3533	987371	35.78 (34.61, 36.98)
		MASLD+low MS-NO	88	14007	62.83 (50.39, 77.40)
		MASLD+low MS-O	189	24819	76.15 (65.68, 87.82)
		MASLD+low MS	277	38826	71.34 (63.19, 80.26)
	Older	Healthy	12794	1692651	75.59 (74.28, 76.91)
		MASLD+normal MS	12759	1076490	118.52 (116.48, 120.60)
		MASLD+low MS-NO	544	30446	178.68 (163.98, 194.34)
		MASLD+low MS-O	923	51701	178.53 (167.19, 190.43)
		MASLD+low MS	1467	82146	178.58 (169.56, 187.96)
Cirrhosis	Younger	Healthy	216	2022697	1.07 (0.93, 1.22)
		MASLD+normal MS	423	1003365	4.22 (3.82, 4.64)
		MASLD+low MS-NO	14	14335	9.77 (5.34, 16.39)
		MASLD+low MS-O	26	25702	10.12 (6.61, 14.82)
		MASLD+low MS	40	40038	9.99 (7.14, 13.60)
	Older	Healthy	244	1754663	1.39 (1.22, 1.58)
		MASLD+normal MS	686	1135648	6.04 (5.60, 6.51)
		MASLD+low MS-NO	28	33086	8.46 (5.62, 12.23)
		MASLD+low MS-O	77	55674	13.83 (10.91, 17.29)
		MASLD+low MS	105	88760	11.83 (9.68, 14.32)
HCC	Younger	Healthy	39	2023939	0.19 (0.14, 0.26)
		MASLD+normal MS	52	1006156	0.52 (0.39, 0.68)
		MASLD+low MS-NO	2	14488	1.38 (0.17, 4.99)
		MASLD+low MS-O	4	25886	1.55 (0.42, 3.96)
		MASLD+low MS	6	40374	1.49 (0.55, 3.23)
	Older	Healthy	76	1756009	0.43 (0.34, 0.54)
		MASLD+normal MS	200	1139431	1.76 (1.52, 2.02)
		MASLD+low MS-NO	11	33316	3.30 (1.65, 5.91)
		MASLD+low MS-O	17	56092	3.03 (1.77, 4.85)
		MASLD+low MS	28	89407	3.13 (2.08, 4.53)
CVD	Younger	Healthy	4922	1985610	24.79 (24.10, 25.49)
		MASLD+normal MS	5365	959953	55.89 (54.40, 57.40)
		MASLD+low MS-NO	113	13218	85.49 (70.46, 102.78)
		MASLD+low MS-O	238	23476	101.38 (88.91, 115.11)
		MASLD+low MS	351	36694	95.66 (85.91, 106.21)

	Older	Healthy	13515	1644891	82.16 (80.78, 83.56)
		MASLD+normal MS	13941	1007007	138.44 (136.15, 140.76)
		MASLD+low MS-NO	508	28086	180.87 (165.48, 197.31)
		MASLD+low MS-O	932	46654	199.77 (187.15, 213.02)
		MASLD+low MS	1440	74740	192.67 (182.84, 202.88)
CKD	Younger	Healthy	1568	2016647	7.78 (7.40, 8.17)
		MASLD+normal MS	1953	996722	19.59 (18.73, 20.48)
		MASLD+low MS-NO	47	14155	33.20 (24.40, 44.15)
		MASLD+low MS-O	134	25109	53.37 (44.71, 63.21)
		MASLD+low MS	181	39264	46.10 (39.63, 53.33)
	Older	Healthy	6399	1728706	37.02 (36.11, 37.93)
		MASLD+normal MS	8469	1099521	77.02 (75.39, 78.68)
		MASLD+low MS-NO	325	31533	103.07 (92.16, 114.90)
		MASLD+low MS-O	742	52066	142.51 (132.44, 153.15)
		MASLD+low MS	1067	83599	127.63 (120.09, 135.53)
Non-liver cancers	Younger	Healthy	6775	1965126	34.48 (33.66, 35.31)
		MASLD+normal MS	4592	969100	47.38 (46.02, 48.77)
		MASLD+low MS-NO	58	14002	41.42 (31.45, 53.55)
		MASLD+low MS-O	136	24670	55.13 (46.25, 65.21)
		MASLD+low MS	194	38672	50.17 (43.35, 57.74)
	Older	Healthy	13946	1622115	85.97 (84.55, 87.41)
		MASLD+normal MS	11843	1024477	115.60 (113.53, 117.70)
		MASLD+low MS-NO	326	29744	109.60 (98.03, 122.17)
		MASLD+low MS-O	564	50618	111.42 (102.42, 121.01)
		MASLD+low MS	890	80362	110.75 (103.59, 118.27)
Obesity-related cancers	Younger	Healthy	2162	2005937	10.78 (10.33, 11.24)
		MASLD+normal MS	1590	99353	160.04 (152.26, 168.10)
		MASLD+low MS-NO	22	14334	15.35 (9.62, 23.24)
		MASLD+low MS-O	57	25436	22.41 (16.97, 29.03)
		MASLD+low MS	79	39770	19.86 (15.73, 24.76)
	Older	Healthy	4416	1712555	25.79 (25.03, 26.56)
		MASLD+normal MS	3981	1101976	36.13 (35.01, 37.27)
		MASLD+low MS-NO	95	32384	29.34 (23.73, 35.86)
		MASLD+low MS-O	213	54227	39.28 (34.18, 44.92)
		MASLD+low MS	308	86612	35.56 (31.70, 39.76)

Table S9. Incidence of clinical outcomes according to categories of MASLD and muscle mass (MM) stratified by age.

Outcomes		Groups	Incidence		
			Incident cases	PYs	Incidence (10,000 PYs)
Death	Younger	Healthy	3971	2002063	19.83 (19.22, 20.46)
		MASLD+normal MM	3797	1024678	37.06 (35.89, 38.25)
		MASLD+low MM-NO	11	1227	89.65 (44.75, 160.41)
		MASLD+low MM-O	2	292	68.49 (8.29, 247.42)
		MASLD+low MM	13	1519	85.58 (45.57, 146.35)
	Older	Healthy	12794	1692651	75.59 (74.28, 76.91)
		MASLD+normal MM	14130	1,151,767	122.68 (120.67, 124.72)
		MASLD+low MM-NO	76	5622	135.18 (106.51, 169.20)
		MASLD+low MM-O	20	1247	160.38 (97.97, 247.70)
		MASLD+low MM	96	6870	139.74 (113.19, 170.64)
Cirrhosis	Younger	Healthy	216	2022697	1.07 (0.93, 1.22)
		MASLD+normal MM	463	1041827	4.44 (4.05, 4.87)
		MASLD+low MM-NO	0	1278	—
		MASLD+low MM-O	0	297	—
		MASLD+low MM	0	1576	—
	Older	Healthy	244	1754663	1.39 (1.22, 1.58)
		MASLD+normal MM	786	1217059	6.46 (6.01, 6.93)
		MASLD+low MM-NO	4	6013	6.65 (1.81, 17.03)
		MASLD+low MM-O	1	1336	7.49 (0.19, 41.70)
		MASLD+low MM	5	7349	6.80 (2.21, 15.88)
HCC	Younger	Healthy	39	2023939	0.19 (0.14, 0.26)
		MASLD+normal MM	58	1044954	0.56 (0.42, 0.72)
		MASLD+low MM-NO	0	1278	—
		MASLD+low MM-O	0	297	—
		MASLD+low MM	0	1576	—
	Older	Healthy	76	1756009	0.43 (0.34, 0.54)
		MASLD+normal MM	226	1221450	1.85 (1.62, 2.11)
		MASLD+low MM-NO	2	6025	3.32 (0.40, 11.99)
		MASLD+low MM-O	0	1362	—
		MASLD+low MM	2	7388	2.71 (0.33, 9.78)
CVD	Younger	Healthy	4922	1985610	24.79 (24.10, 25.49)
		MASLD+normal MM	5712	995161	57.40 (55.92, 58.91)
		MASLD+low MM-NO	3	1192	25.17 (5.19, 73.55)
		MASLD+low MM-O	1	294	34.01 (0.86, 189.51)
		MASLD+low MM	4	1485	26.94 (7.34, 68.97)

	Older	Healthy	13515	1644891	82.16 (80.78, 83.56)
		MASLD+normal MM	15308	1075217	142.37 (140.12, 144.64)
		MASLD+low MM-NO	58	5338	108.65 (82.51, 140.46)
		MASLD+low MM-O	15	1192	125.84 (70.43, 207.55)
		MASLD+low MM	73	6530	111.79 (87.63, 140.56)
CKD	Younger	Healthy	1568	2016647	7.78 (7.40, 8.17)
		MASLD+normal MM	2130	1034429	20.59 (19.73, 21.48)
		MASLD+low MM-NO	3	1262	23.77 (4.90, 69.47)
		MASLD+low MM-O	1	294	34.01 (0.86, 189.51)
		MASLD+low MM	4	1556	25.71 (7.00, 65.82)
	Older	Healthy	6399	1728706	37.02 (36.11, 37.93)
		MASLD+normal MM	9489	1175963	80.69 (79.08, 82.33)
		MASLD+low MM-NO	43	5818	73.91 (53.49, 99.55)
		MASLD+low MM-O	4	1339	29.87 (8.14, 76.49)
		MASLD+low MM	47	7157	65.67 (48.25, 87.33)
Non-liver cancers	Younger	Healthy	6775	1965126	34.48 (33.66, 35.31)
		MASLD+normal MM	4778	1006278	47.48 (46.15, 48.85)
		MASLD+low MM-NO	5	1217	41.08 (13.34, 95.88)
		MASLD+low MM-O	3	277	108.30 (22.33, 316.51)
		MASLD+low MM	8	1493	53.58 (23.13, 105.58)
	Older	Healthy	13946	1622115	85.97 (84.55, 87.41)
		MASLD+normal MM	12672	1098253	115.38 (113.38, 117.41)
		MASLD+low MM-NO	52	5389	96.49 (72.07, 126.54)
		MASLD+low MM-O	9	1196	75.25 (34.41, 142.85)
		MASLD+low MM	61	6585	92.63 (70.86, 118.99)
Obesity-related cancers	Younger	Healthy	2162	2005937	10.78 (10.33, 11.24)
		MASLD+normal MM	1668	1031764	16.17 (15.40, 16.96)
		MASLD+low MM-NO	0	1251	—
		MASLD+low MM-O	1	287	34.84 (0.88, 194.13)
		MASLD+low MM	1	1538	6.50 (0.16, 36.23)
	Older	Healthy	4416	1712555	25.79 (25.03, 26.56)
		MASLD+normal MM	4260	1181489	36.06 (34.98, 37.16)
		MASLD+low MM-NO	24	5806	41.34 (26.49, 61.51)
		MASLD+low MM-O	5	1292	38.70 (12.57, 90.31)
		MASLD+low MM	29	7098	40.86 (27.36, 58.68)

Table S10. Incidence of clinical outcomes according to categories of MASLD and physical performance (PP) stratified by age.

Outcomes		Groups	Incidence		
			Incident cases	PYs	Incidence (10,000 PYs)
Death	Younger	Healthy	3971	2002063	19.83 (19.22, 20.46)
		MASLD+normal PP	2976	922491	32.26 (31.11, 33.44)
		MASLD+low PP-NO	218	24332	89.59 (78.09, 102.31)
		MASLD+low PP-O	616	79374	77.61 (71.60, 83.99)
		MASLD+low PP	834	103706	80.42 (75.05, 86.07)
	Older	Healthy	12794	1692651	75.59 (74.28, 76.91)
		MASLD+normal PP	10336	981024	105.36 (103.34, 107.41)
		MASLD+low PP-NO	1137	45731	248.63 (234.38, 263.51)
		MASLD+low PP-O	2753	131881	208.75 (201.02, 216.70)
		MASLD+low PP	3890	177612	219.02 (212.19, 226.01)
Cirrhosis	Younger	Healthy	216	2022697	1.07 (0.93, 1.22)
		MASLD+normal PP	361	935912	3.86 (3.47, 4.28)
		MASLD+low PP-NO	19	25371	7.49 (4.51, 11.69)
		MASLD+low PP-O	83	82120	10.11 (8.05, 12.53)
		MASLD+low PP	102	107490	9.49 (7.74, 11.52)
	Older	Healthy	244	1754663	1.39 (1.22, 1.58)
		MASLD+normal PP	539	1028260	5.24 (4.81, 5.70)
		MASLD+low PP-NO	62	51678	12.00 (9.20, 15.38)
		MASLD+low PP-O	190	144469	13.15 (11.35, 15.16)
		MASLD+low PP	252	196147	12.85 (11.31, 14.54)
HCC	Younger	Healthy	39	2023939	0.19 (0.14, 0.26)
		MASLD+normal PP	44	938093	0.47 (0.34, 0.63)
		MASLD+low PP-NO	5	25620	1.95 (0.63, 4.55)
		MASLD+low PP-O	9	82817	1.09 (0.50, 2.06)
		MASLD+low PP	14	108437	1.29 (0.71, 2.17)
	Older	Healthy	76	1756009	0.43 (0.34, 0.54)
		MASLD+normal PP	165	1031021	1.60 (1.37, 1.86)
		MASLD+low PP-NO	20	52102	3.84 (2.34, 5.93)
		MASLD+low PP-O	43	145715	2.95 (2.14, 3.97)
		MASLD+low PP	63	197817	3.18 (2.45, 4.07)
CVD	Younger	Healthy	4922	1985610	24.79 (24.10, 25.49)
		MASLD+normal PP	4674	899991	51.93 (50.46, 53.44)
		MASLD+low PP-NO	268	22414	119.57 (105.68, 134.77)
		MASLD+low PP-O	774	74241	104.26 (97.04, 111.87)
		MASLD+low PP	1042	96655	107.81 (101.36, 114.56)

	Older	Healthy	13515	1644891	82.16 (80.78, 83.56)
		MASLD+normal PP	11767	927320	126.89 (124.61, 129.21)
		MASLD+low PP-NO	973	39291	247.64 (232.32, 263.70)
		MASLD+low PP-O	2641	115136	229.38 (220.72, 238.30)
		MASLD+low PP	3614	154427	234.03 (226.46, 241.78)
CKD	Younger	Healthy	1568	2016647	7.78 (7.40, 8.17)
		MASLD+normal PP	1582	930788	17.00 (16.17, 17.86)
		MASLD+low PP-NO	108	24961	43.27 (35.49, 52.24)
		MASLD+low PP-O	444	80237	55.34 (50.31, 60.73)
		MASLD+low PP	552	105198	52.47 (48.19, 57.04)
	Older	Healthy	6399	1728706	37.02 (36.11, 37.93)
		MASLD+normal PP	6862	1000342	68.60 (66.98, 70.24)
		MASLD+low PP-NO	579	48895	118.42 (108.97, 128.47)
		MASLD+low PP-O	2095	133884	156.48 (149.85, 163.33)
		MASLD+low PP	2674	182778	146.30 (140.80, 151.95)
Non-liver cancers	Younger	Healthy	6775	1965126	34.48 (33.66, 35.31)
		MASLD+normal PP	4208	904920	46.50 (45.11, 47.93)
		MASLD+low PP-NO	140	24144	57.99 (48.78, 68.43)
		MASLD+low PP-O	438	78708	55.65 (50.56, 61.11)
		MASLD+low PP	578	102851	56.20 (51.71, 60.97)
	Older	Healthy	13946	1622115	85.97 (84.55, 87.41)
		MASLD+normal PP	10668	929419	114.78 (112.61, 116.98)
		MASLD+low PP-NO	600	45297	132.46 (122.07, 143.49)
		MASLD+low PP-O	1465	130122	112.59 (106.89, 118.50)
		MASLD+low PP	2065	175419	117.72 (112.70, 122.91)
Obesity-related cancers	Younger	Healthy	2162	2005937	10.78 (10.33, 11.24)
		MASLD+normal PP	1428	926836	15.41 (14.62, 16.23)
		MASLD+low PP-NO	44	25225	17.44 (12.67, 23.42)
		MASLD+low PP-O	197	81241	24.25 (20.98, 27.88)
		MASLD+low PP	241	106466	22.64 (19.87, 25.68)
	Older	Healthy	4416	1712555	25.79 (25.03, 26.56)
		MASLD+normal PP	3526	998531	35.31 (34.16, 36.50)
		MASLD+low PP-NO	194	50189	38.65 (33.41, 44.49)
		MASLD+low PP-O	569	139868	40.68 (37.41, 44.17)
		MASLD+low PP	763	190057	40.15 (37.35, 43.10)

Table S11. Incidence of clinical outcomes according to categories of MASLD and muscle strength (MS) stratified by sex.

Outcomes		Groups	Incidence		
			Incident cases	PYs	Incidence (10,000 PYs)
Death	Male	Healthy	8408	1338500	62.82 (61.48, 64.17)
		MASLD+normal MS	11368	1294791	87.80 (86.19, 89.43)
		MASLD+low MS-NO	470	27946	168.18 (153.32, 184.10)
		MASLD+low MS-O	602	29806	201.97 (186.16, 218.77)
		MASLD+low MS	1072	57752	185.62 (174.67, 197.07)
	Female	Healthy	8357	2356215	35.47 (34.71, 36.24)
		MASLD+normal MS	4924	769070	64.03 (62.25, 65.84)
		MASLD+low MS-NO	162	16508	98.13 (83.60, 114.46)
		MASLD+low MS-O	510	46713	109.18 (99.91, 119.08)
		MASLD+low MS	672	63221	106.29 (98.41, 114.64)
Cirrhosis	Male	Healthy	260	1380233	1.88 (1.66, 2.13)
		MASLD+normal MS	789	1348172	5.85 (5.45, 6.28)
		MASLD+low MS-NO	32	30182	10.60 (7.25, 14.97)
		MASLD+low MS-O	47	32479	14.47 (10.63, 19.24)
		MASLD+low MS	79	62661	12.61 (9.98, 15.71)
	Female	Healthy	200	2397127	0.83 (0.72, 0.96)
		MASLD+normal MS	320	790840	4.05 (3.62, 4.51)
		MASLD+low MS-NO	10	17239	5.80 (2.78, 10.67)
		MASLD+low MS-O	56	48898	11.45 (8.65, 14.87)
		MASLD+low MS	66	66137	9.98 (7.72, 12.70)
HCC	Male	Healthy	68	1381739	0.49 (0.38, 0.62)
		MASLD+normal MS	210	1352876	1.55 (1.35, 1.78)
		MASLD+low MS-NO	13	30452	4.27 (2.27, 7.30)
		MASLD+low MS-O	15	32795	4.57 (2.56, 7.54)
		MASLD+low MS	28	63248	4.43 (2.94, 6.40)
	Female	Healthy	47	2398208	0.20 (0.14, 0.26)
		MASLD+normal MS	42	792710	0.53 (0.38, 0.72)
		MASLD+low MS-NO	0	17351	0.00 (0.00, 2.13)
		MASLD+low MS-O	6	49182	1.22 (0.45, 2.66)
		MASLD+low MS	6	66534	0.90 (0.33, 1.96)
CVD	Male	Healthy	9850	1295255	76.05 (74.55, 77.56)
		MASLD+normal MS	13879	1217603	113.99 (112.10, 115.90)
		MASLD+low MS-NO	424	25688	165.06 (149.72, 181.54)
		MASLD+low MS-O	575	26208	219.40 (201.83, 238.09)
		MASLD+low MS	999	51896	192.50 (180.75, 204.82)

	Female	Healthy	8587	2335246	36.77 (36.00, 37.56)
		MASLD+normal MS	5427	749356	72.42 (70.51, 74.38)
		MASLD+low MS-NO	197	15616	126.15 (109.15, 145.05)
		MASLD+low MS-O	595	43922	135.47 (124.80, 146.80)
		MASLD+low MS	792	59538	133.02 (123.92, 142.62)
CKD	Male	Healthy	3395	1366734	24.84 (24.01, 25.69)
		MASLD+normal MS	6291	1322640	47.56 (46.40, 48.75)
		MASLD+low MS-NO	242	29081	83.22 (73.06, 94.39)
		MASLD+low MS-O	368	30750	119.67 (107.76, 132.55)
		MASLD+low MS	610	59832	101.95 (94.02, 110.37)
	Female	Healthy	4572	2378620	19.22 (18.67, 19.79)
		MASLD+normal MS	4131	773603	53.40 (51.78, 55.05)
		MASLD+low MS-NO	130	16607	78.28 (65.40, 92.95)
		MASLD+low MS-O	508	46424	109.43 (100.12, 119.37)
		MASLD+low MS	638	63031	101.22 (93.52, 109.39)
Non-liver cancers	Male	Healthy	10871	1284740	84.62 (83.03, 86.22)
		MASLD+normal MS	11912	1246186	95.59 (93.88, 97.32)
		MASLD+low MS-NO	300	27389	109.53 (97.49, 122.66)
		MASLD+low MS-O	368	29237	125.87 (113.33, 139.41)
		MASLD+low MS	668	56625	117.97 (109.19, 127.26)
	Female	Healthy	9850	2302500	42.78 (41.94, 43.63)
		MASLD+normal MS	4523	747391	60.52 (58.77, 62.31)
		MASLD+low MS-NO	84	16357	51.35 (40.96, 63.58)
		MASLD+low MS-O	332	46051	72.09 (64.55, 80.28)
		MASLD+low MS	416	62408	66.66 (60.41, 73.38)
		Healthy			
Obesity-related cancers	Male	MASLD+normal MS	2520	1358473	18.55 (17.83, 19.29)
		MASLD+low MS-NO	3421	1323683	25.84 (24.99, 26.73)
		MASLD+low MS-O	85	29718	28.60 (22.85, 35.37)
		MASLD+low MS	122	31831	38.33 (31.83, 45.76)
		MASLD+low MS	207	61549	33.63 (29.21, 38.54)
	Female	Healthy	4058	2360019	17.19 (16.67, 17.73)
		MASLD+normal MS	2150	771824	27.86 (26.69, 29.06)
		MASLD+low MS-NO	32	17001	18.82 (12.87, 26.57)
		MASLD+low MS-O	148	47833	30.94 (26.16, 36.35)
		MASLD+low MS	180	64833	27.76 (23.86, 32.13)

Table S12. Incidence of clinical outcomes according to categories of MASLD and muscle mass (MM) stratified by sex.

Outcomes		Groups	Incidence		
			Incident cases	PYs	incidence (10,000 PYs)
Death	Male	Healthy	8408	1338500	62.82 (61.48, 64.17)
		MASLD+normal MM	12426	1351773	91.92 (90.31, 93.55)
		MASLD+low MM-NO	14	725	193.10 (105.57, 323.99)
		MASLD+low MM-O	0	43	—
		MASLD+low MM	14	769	182.05 (99.53, 305.46)
	Female	Healthy	8357	2356215	35.47 (34.71, 36.24)
		MASLD+normal MM	5501	824672	66.71 (64.95, 68.49)
		MASLD+low MM-NO	73	6124	119.20 (93.44, 149.88)
		MASLD+low MM-O	22	1496	147.06 (92.16, 222.65)
		MASLD+low MM	95	7620	124.67 (100.87, 152.40)
Cirrhosis	Male	Healthy	260	1380233	1.88 (1.66, 2.13)
		MASLD+normal MM	868	1409973	6.16 (5.75, 6.58)
		MASLD+low MM-NO	0	818	—
		MASLD+low MM-O	0	43	—
		MASLD+low MM	0	861	—
	Female	Healthy	200	2397127	0.83 (0.72, 0.96)
		MASLD+normal MM	381	848913	4.49 (4.05, 4.96)
		MASLD+low MM-NO	4	6474	6.18 (1.68, 15.82)
		MASLD+low MM-O	1	1590	6.29 (0.16, 35.04)
		MASLD+low MM	5	8064	6.20 (2.01, 14.47)
HCC	Male	Healthy	68	1381739	0.49 (0.38, 0.62)
		MASLD+normal MM	237	1415276	1.67 (1.47, 1.90)
		MASLD+low MM-NO	1	804	12.44 (0.31, 69.30)
		MASLD+low MM-O	0	43	—
		MASLD+low MM	1	848	11.79 (0.30, 65.70)
	Female	Healthy	47	2398208	0.20 (0.14, 0.26)
		MASLD+normal MM	47	851128	0.55 (0.41, 0.73)
		MASLD+low MM-NO	1	6499	1.54 (0.04, 8.57)
		MASLD+low MM-O	0	1617	—
		MASLD+low MM	1	8115	1.23 (0.03, 6.87)
CVD	Male	Healthy	9850	1295255	76.05 (74.55, 77.56)
		MASLD+normal MM	14871	1268814	117.20 (115.33, 119.10)
		MASLD+low MM-NO	7	642	109.03 (43.84, 224.65)
		MASLD+low MM-O	0	43	—
		MASLD+low MM	7	685	102.19 (41.09, 210.55)

	Female	Healthy	8587	2335246	36.77 (36.00, 37.56)
		MASLD+normal MM	6149	801564	76.71 (74.81, 78.65)
		MASLD+low MM-NO	54	5887	91.73 (68.91, 119.68)
		MASLD+low MM-O	16	1442	110.96 (63.42, 180.19)
		MASLD+low MM	70	7330	95.50 (74.45, 120.66)
CKD	Male	Healthy	3395	1366734	24.84 (24.01, 25.69)
		MASLD+normal MM	6897	1381637	49.92 (48.75, 51.11)
		MASLD+low MM-NO	4	791	50.57 (13.78, 129.48)
		MASLD+low MM-O	0	43	—
		MASLD+low MM	4	835	47.90 (13.05, 122.65)
	Female	Healthy	4572	2378620	19.22 (18.67, 19.79)
		MASLD+normal MM	4722	828756	56.98 (55.36, 58.63)
		MASLD+low MM-NO	42	6289	66.78 (48.13, 90.27)
		MASLD+low MM-O	5	1590	31.45 (10.21, 73.39)
		MASLD+low MM	47	7879	59.65 (43.83, 79.32)
Non-liver cancers	Male	Healthy	10871	1284740	84.62 (83.03, 86.22)
		MASLD+normal MM	12573	1302005	96.57 (94.89, 98.27)
		MASLD+low MM-NO	7	763	91.74 (36.89, 189.03)
		MASLD+low MM-O	0	43	—
		MASLD+low MM	7	806	86.85 (34.92, 178.94)
	Female	Healthy	9850	2302500	42.78 (41.94, 43.63)
		MASLD+normal MM	4877	802527	60.77 (59.08, 62.50)
		MASLD+low MM-NO	50	5843	85.57 (63.51, 112.82)
		MASLD+low MM-O	12	1429	83.97 (43.39, 146.69)
		MASLD+low MM	62	7272	85.26 (65.37, 109.30)
Obesity-related cancers	Male	Healthy	2520	1358473	18.55 (17.83, 19.29)
		MASLD+normal MM	3627	1384384	26.20 (25.35, 27.07)
		MASLD+low MM-NO	1	804	12.44 (0.31, 69.30)
		MASLD+low MM-O	0	43	—
		MASLD+low MM	1	848	11.79 (0.30, 65.70)
	Female	Healthy	4058	2360019	17.19 (16.67, 17.73)
		MASLD+normal MM	2301	828869	27.76 (26.64, 28.92)
		MASLD+low MM-NO	23	6253	36.78 (23.32, 55.19)
		MASLD+low MM-O	6	1536	39.06 (14.34, 85.02)
		MASLD+low MM	29	7789	37.23 (24.93, 53.47)

Table S13. Incidence of clinical outcomes according to categories of MASLD and physical performance (PP) stratified by sex.

Outcomes		Groups	Incidence		
			Incident cases	PYs	Incidence (10,000 PYs)
Death	Male	Healthy	8408	1338500	62.82 (61.48, 64.17)
		MASLD+normal PP	9492	1219138	77.86 (76.30, 79.44)
		MASLD+low PP-NO	1053	47728	220.63 (207.50, 234.36)
		MASLD+low PP-O	1895	85677	221.18 (211.33, 231.37)
		MASLD+low PP	2948	133404	220.98 (213.08, 229.11)
	Female	Healthy	8357	2356215	35.47 (34.71, 36.24)
		MASLD+normal PP	3820	684377	55.82 (54.06, 57.62)
		MASLD+low PP-NO	302	22336	135.21 (120.39, 151.35)
		MASLD+low PP-O	1474	125578	117.38 (111.46, 123.53)
		MASLD+low PP	1776	147914	120.07 (114.55, 125.79)
Cirrhosis	Male	Healthy	260	1380233	1.88 (1.66, 2.13)
		MASLD+normal PP	641	1263180	5.07 (4.69, 5.48)
		MASLD+low PP-NO	63	53117	11.86 (9.11, 15.17)
		MASLD+low PP-O	164	94537	17.35 (14.79, 20.22)
		MASLD+low PP	227	147653	15.37 (13.44, 17.51)
	Female	Healthy	200	2397127	0.83 (0.72, 0.96)
		MASLD+normal PP	259	700993	3.69 (3.26, 4.17)
		MASLD+low PP-NO	18	23932	7.52 (4.46, 11.89)
		MASLD+low PP-O	109	132052	8.25 (6.78, 9.96)
		MASLD+low PP	127	155984	8.14 (6.79, 9.69)
HCC	Male	Healthy	68	1381739	0.49 (0.38, 0.62)
		MASLD+normal PP	176	1266720	1.39 (1.19, 1.61)
		MASLD+low PP-NO	23	53623	4.29 (2.72, 6.44)
		MASLD+low PP-O	39	95782	4.07 (2.90, 5.57)
		MASLD+low PP	62	149404	4.15 (3.18, 5.32)
	Female	Healthy	47	2398208	0.20 (0.14, 0.26)
		MASLD+normal PP	33	702394	0.47 (0.32, 0.66)
		MASLD+low PP-NO	2	24098	0.83 (0.10, 3.00)
		MASLD+low PP-O	13	132751	0.98 (0.52, 1.67)
		MASLD+low PP	15	156849	0.96 (0.54, 1.58)
CVD	Male	Healthy	9850	1295255	76.05 (74.55, 77.56)
		MASLD+normal PP	12138	1157188	104.89 (103.03, 106.78)
		MASLD+low PP-NO	894	40947	218.33 (204.25, 233.12)
		MASLD+low PP-O	1846	71364	258.67 (247.01, 270.75)
		MASLD+low PP	2740	112311	243.97 (234.92, 253.28)

	Female	Healthy	8587	2335246	36.77 (36.00, 37.56)
		MASLD+normal PP	4303	670122	64.21 (62.31, 66.16)
		MASLD+low PP-NO	347	20758	167.16 (150.04, 185.71)
		MASLD+low PP-O	1569	118013	132.95 (126.45, 139.70)
		MASLD+low PP	1916	138771	138.07 (131.96, 144.39)
CKD	Male	Healthy	3395	1366734	24.84 (24.01, 25.69)
		MASLD+normal PP	5270	1242723	42.41 (41.27, 43.57)
		MASLD+low PP-NO	467	50907	91.74 (83.60, 100.45)
		MASLD+low PP-O	1164	88841	131.02 (123.60, 138.77)
		MASLD+low PP	1631	139748	116.71 (111.11, 122.51)
	Female	Healthy	4572	2378620	19.22 (18.67, 19.79)
		MASLD+normal PP	3174	688406	46.11 (44.52, 47.74)
		MASLD+low PP-NO	220	22949	95.86 (83.61, 109.40)
		MASLD+low PP-O	1375	125280	109.75 (104.03, 115.71)
		MASLD+low PP	1595	148229	107.60 (102.39, 113.02)
Non-liver cancers	Male	Healthy	10871	1284740	84.62 (83.03, 86.22)
		MASLD+normal PP	10978	1170095	93.82 (92.07, 95.59)
		MASLD+low PP-NO	572	47446	120.56 (110.88, 130.86)
		MASLD+low PP-O	1030	85269	120.79 (113.53, 128.40)
		MASLD+low PP	1602	132716	120.71 (114.87, 126.77)
	Female	Healthy	9850	2302500	42.78 (41.94, 43.63)
		MASLD+normal PP	3898	664244	58.68 (56.86, 60.55)
		MASLD+low PP-NO	168	21994	76.38 (65.27, 88.85)
		MASLD+low PP-O	873	123560	70.65 (66.04, 75.50)
		MASLD+low PP	1041	145554	71.52 (67.24, 76.00)
Obesity-related cancers	Male	Healthy	2520	1358473	18.55 (17.83, 19.29)
		MASLD+normal PP	3136	1240531	25.28 (24.40, 26.18)
		MASLD+low PP-NO	165	52026	31.71 (27.06, 36.94)
		MASLD+low PP-O	327	92676	35.28 (31.56, 39.32)
		MASLD+low PP	492	144701	34.00 (31.06, 37.14)
	Female	Healthy	4058	2360019	17.19 (16.67, 17.73)
		MASLD+normal PP	1818	684836	26.55 (25.34, 27.80)
		MASLD+low PP-NO	73	23388	31.21 (24.47, 39.25)
		MASLD+low PP-O	439	128433	34.18 (31.06, 37.53)
		MASLD+low PP	512	151821	33.72 (30.87, 36.78)

Table S14. Incidence of clinical outcomes according to categories of MASLD and muscle strength (MS) stratified by diabetes.

			Incidence		
**Outcomes**		**Groups**	**incident cases**	**PYs**	**incidence (10,000 person-years)**
Death	DM	Healthy	1591	129587	122.77 (116.82, 128.96)
		MASLD+normal MS	4876	326236	149.46 (145.30, 153.72)
		MASLD+low MS-NO	193	9332	206.82 (178.67, 238.14)
		MASLD+low MS-O	519	24947	208.04 (190.53, 226.73)
		MASLD+low MS	712	34279	207.71 (192.73, 223.54)
	Non-DM	Healthy	15174	3565127	42.56 (41.89, 43.24)
		MASLD+normal MS	11416	1737625	65.70 (64.50, 66.92)
		MASLD+low MS-NO	439	35121	125.00 (113.58, 137.25)
		MASLD+low MS-O	593	51573	114.98 (105.91, 124.62)
		MASLD+low MS	1032	86694	119.04 (111.89, 126.53)
Cirrhosis	DM	Healthy	98	135755	7.22 (5.86, 8.80)
		MASLD+normal MS	541	343791	15.74 (14.44, 17.12)
		MASLD+low MS-NO	20	10001	20.00 (12.22, 30.89)
		MASLD+low MS-O	73	26878	27.16 (21.29, 34.15)
		MASLD+low MS	93	36879	25.22 (20.35, 30.89)
	Non-DM	Healthy	362	3641605	0.99 (0.89, 1.10)
		MASLD+normal MS	568	1795221	3.16 (2.91, 3.44)
		MASLD+low MS-NO	22	37420	5.88 (3.68, 8.90)
		MASLD+low MS-O	30	54499	5.50 (3.71, 7.86)
		MASLD+low MS	52	91919	5.66 (4.23, 7.42)
HCC	DM	Healthy	26	136379	1.91 (1.25, 2.79)
		MASLD+normal MS	148	346879	4.27 (3.61, 5.01)
		MASLD+low MS-NO	4	10213	3.92 (1.07, 10.03)
		MASLD+low MS-O	12	27276	4.40 (2.27, 7.68)
		MASLD+low MS	16	37488	4.27 (2.44, 6.93)
	Non-DM	Healthy	89	3643569	0.24 (0.20, 0.30)
		MASLD+normal MS	104	1798708	0.58 (0.47, 0.70)
		MASLD+low MS-NO	9	37591	2.39 (1.09, 4.54)
		MASLD+low MS-O	9	54702	1.65 (0.75, 3.12)
		MASLD+low MS	18	92293	1.95 (1.16, 3.08)
CVD	DM	Healthy	2066	118413	174.47 (167.03, 182.16)
		MASLD+normal MS	6154	286972	214.45 (209.12, 219.87)
		MASLD+low MS-NO	210	7909	265.52 (230.82, 303.96)
		MASLD+low MS-O	593	20976	282.70 (260.41, 306.40)
		MASLD+low MS	803	28886	277.99 (259.09, 297.90)

	Non-DM	Healthy	16371	3512088	46.61 (45.90, 47.33)
		MASLD+normal MS	13152	1679987	78.29 (76.95, 79.64)
		MASLD+low MS-NO	411	33394	123.08 (111.46, 135.57)
		MASLD+low MS-O	577	49154	117.39 (108.00, 127.37)
		MASLD+low MS	988	82548	119.69 (112.34, 127.39)
CKD	DM	Healthy	1300	130495	99.62 (94.28, 105.19)
		MASLD+normal MS	4374	325314	134.45 (130.50, 138.50)
		MASLD+low MS-NO	152	9292	163.58 (138.61, 191.75)
		MASLD+low MS-O	507	24371	208.03 (190.32, 226.96)
		MASLD+low MS	659	33664	195.76 (181.10, 211.29)
	Non-DM	Healthy	6667	3614859	18.44 (18.00, 18.89)
		MASLD+normal MS	6048	1770929	34.15 (33.30, 35.02)
		MASLD+low MS-NO	220	36396	60.45 (52.72, 68.98)
		MASLD+low MS-O	369	52803	69.88 (62.93, 77.39)
		MASLD+low MS	589	89199	66.03 (60.81, 71.59)
Non-liver cancers	DM	Healthy	1512	123100	122.83 (116.71, 129.18)
		MASLD+normal MS	3946	312590	126.24 (122.33, 130.24)
		MASLD+low MS-NO	107	9107	117.49 (96.29, 141.98)
		MASLD+low MS-O	292	24744	118.01 (104.86, 132.35)
		MASLD+low MS	399	33852	117.87 (106.58, 130.02)
	Non-DM	Healthy	19209	3464140	55.45 (54.67, 56.24)
		MASLD+normal MS	12489	1680987	74.30 (73.00, 75.61)
		MASLD+low MS-NO	277	34639	79.97 (70.83, 89.96)
		MASLD+low MS-O	408	50543	80.72 (73.08, 88.95)
		MASLD+low MS	685	85182	80.42 (74.51, 86.67)
Obesity-related cancers	DM	Healthy	527	131821	39.98 (36.64, 43.54)
		MASLD+normal MS	1610	334148	48.18 (45.86, 50.59)
		MASLD+low MS-NO	32	9919	32.26 (22.07, 45.54)
		MASLD+low MS-O	130	26329	49.38 (41.25, 58.63)
		MASLD+low MS	162	36248	44.69 (38.07, 52.13)
	Non-DM	Healthy	6051	3586670	16.87 (16.45, 17.30)
		MASLD+normal MS	3961	1761359	22.49 (21.79, 23.20)
		MASLD+low MS-NO	85	36800	23.10 (18.45, 28.56)
		MASLD+low MS-O	140	53334	26.25 (22.08, 30.98)
		MASLD+low MS	225	90134	24.96 (21.81, 28.45)

Table S15. Incidence of clinical outcomes according to categories of MASLD and muscle mass (MM) stratified by diabetes.

Outcomes		Groups	Incidence		
			Incident cases	PYs	incidence (10,000 PYs)
Death	DM	Healthy	1591	129587	122.77 (116.82, 128.96)
		MASLD+normal MM	5567	359436	154.88 (150.84, 159.00)
		MASLD+low MM-NO	17	832	204.33 (119.03, 327.15)
		MASLD+low MM-O	4	246	162.60 (44.30, 416.32)
		MASLD+low MM	21	1078	194.81 (120.59, 297.78)
	Non-DM	Healthy	15174	3565127	42.56 (41.89, 43.24)
		MASLD+normal MM	12360	1817009	68.02 (66.83, 69.23)
		MASLD+low MM-NO	70	6017	116.34 (90.69, 146.98)
		MASLD+low MM-O	18	1293	139.21 (82.51, 220.01)
		MASLD+low MM	88	7310	120.38 (96.55, 148.32)
Cirrhosis	DM	Healthy	98	135755	7.22 (5.86, 8.80)
		MASLD+normal MM	630	379531	16.60 (15.33, 17.95)
		MASLD+low MM-NO	3	883	33.98 (7.01, 99.29)
		MASLD+low MM-O	1	257	38.91 (0.99, 216.80)
		MASLD+low MM	4	1140	35.09 (9.56, 89.84)
	Non-DM	Healthy	362	3641605	0.99 (0.89, 1.10)
		MASLD+normal MM	619	1879355	3.29 (3.04, 3.56)
		MASLD+low MM-NO	1	6409	1.56 (0.04, 8.69)
		MASLD+low MM-O	0	1377	—
		MASLD+low MM	1	7785	1.28 (0.03, 7.16)
HCC	DM	Healthy	26	136379	1.91 (1.25, 2.79)
		MASLD+normal MM	164	383188	4.28 (3.65, 4.99)
		MASLD+low MM-NO	0	910	—
		MASLD+low MM-O	0	269	—
		MASLD+low MM	0	1179	—
	Non-DM	Healthy	89	3643569	0.24 (0.20, 0.30)
		MASLD+normal MM	120	1883216	0.64 (0.53, 0.76)
		MASLD+low MM-NO	2	6394	3.13 (0.38, 11.30)
		MASLD+low MM-O	0	1391	—
		MASLD+low MM	2	7784	2.57 (0.31, 9.28)
CVD	DM	Healthy	2066	118413	174.47 (167.03, 182.16)
		MASLD+normal MM	6934	314911	220.19 (215.04, 225.43)
		MASLD+low MM-NO	18	728	247.25 (146.54, 390.77)
		MASLD+low MM-O	5	219	228.31 (74.13, 532.80)
		MASLD+low MM	23	947	242.87 (153.96, 364.43)

	Non-DM	Healthy	16371	3512088	46.61 (45.90, 47.33)
		MASLD+normal MM	14086	1755467	80.24 (78.92, 81.58)
		MASLD+low MM-NO	43	5801	74.13 (53.64, 99.85)
		MASLD+low MM-O	11	1267	86.82 (43.34, 155.34)
		MASLD+low MM	54	7068	76.40 (57.39, 99.69)
CKD	DM	Healthy	1300	130495	99.62 (94.28, 105.19)
		MASLD+normal MM	5020	357859	140.28 (136.42, 144.21)
		MASLD+low MM-NO	12	851	141.01 (72.86, 246.32)
		MASLD+low MM-O	1	268	37.31 (0.94, 207.90)
		MASLD+low MM	13	1119	116.18 (61.86, 198.66)
	Non-DM	Healthy	6667	3614859	18.44 (18.00, 18.89)
		MASLD+normal MM	6599	1852534	35.62 (34.77, 36.49)
		MASLD+low MM-NO	34	6229	54.58 (37.80, 76.27)
		MASLD+low MM-O	4	1365	29.30 (7.98, 75.03)
		MASLD+low MM	38	7595	50.03 (35.41, 68.67)
Non-liver cancers	DM	Healthy	1512	123100	122.83 (116.71, 129.18)
		MASLD+normal MM	4332	345381	125.43 (121.72, 129.22)
		MASLD+low MM-NO	11	809	135.97 (67.88, 243.29)
		MASLD+low MM-O	2	251	79.68 (9.65, 287.84)
		MASLD+low MM	13	1060	122.64 (65.30, 209.72)
	Non-DM	Healthy	19209	3464140	55.45 (54.67, 56.24)
		MASLD+normal MM	13118	1759151	74.57 (73.30, 75.86)
		MASLD+low MM-NO	46	5796	79.37 (58.11, 105.86)
		MASLD+low MM-O	10	1221	81.90 (39.27, 150.62)
		MASLD+low MM	56	7018	79.79 (60.28, 103.62)
Obesity-related cancers	DM	Healthy	527	131821	39.98 (36.64, 43.54)
		MASLD+normal MM	1762	369302	47.71 (45.51, 49.99)
		MASLD+low MM-NO	8	843	94.90 (40.97, 186.99)
		MASLD+low MM-O	2	251	79.68 (9.65, 287.84)
		MASLD+low MM	10	1094	91.41 (43.83, 168.10)
	Non-DM	Healthy	6051	3586670	16.87 (16.45, 17.30)
		MASLD+normal MM	4166	1843951	22.59 (21.91, 23.29)
		MASLD+low MM-NO	16	6214	25.75 (14.72, 41.81)
		MASLD+low MM-O	4	1329	30.10 (8.20, 77.06)
		MASLD+low MM	20	7542	26.52 (16.20, 40.96)

Table S16. Incidence of clinical outcomes according to categories of MASLD and physical performance (PP) stratified by diabetes.

Outcomes		Groups	Incident cases	PYs	incidence (10,000 PYs)
Death	DM	Healthy	1591	129587	122.77 (116.82, 128.96)
		MASLD+normal PP	3512	271119	129.54 (125.29, 133.89)
		MASLD+low PP-NO	413	16356	252.51 (228.74, 278.07)
		MASLD+low PP-O	1663	73039	227.69 (216.87, 238.90)
		MASLD+low PP	2076	89396	232.23 (222.34, 242.43)
	Non-DM	Healthy	15174	3565127	42.56 (41.89, 43.24)
		MASLD+normal PP	9800	1632396	60.03 (58.85, 61.24)
		MASLD+low PP-NO	942	53707	175.40 (164.37, 186.96)
		MASLD+low PP-O	1706	138216	123.43 (117.64, 129.43)
		MASLD+low PP	2648	191923	137.97 (132.77, 143.33)
Cirrhosis	DM	Healthy	98	135755	7.22 (5.86, 8.80)
		MASLD+normal PP	401	283323	14.15 (12.80, 15.61)
		MASLD+low PP-NO	36	18049	19.95 (13.97, 27.61)
		MASLD+low PP-O	197	79299	24.84 (21.49, 28.56)
		MASLD+low PP	233	97347	23.93 (20.96, 27.21)
	Non-DM	Healthy	362	3641605	0.99 (0.89, 1.10)
		MASLD+normal PP	499	1680850	2.97 (2.71, 3.24)
		MASLD+low PP-NO	45	59000	7.63 (5.56, 10.21)
		MASLD+low PP-O	76	147290	5.16 (4.07, 6.46)
		MASLD+low PP	121	206290	5.87 (4.87, 7.01)
HCC	DM	Healthy	26	136379	1.91 (1.25, 2.79)
		MASLD+normal PP	115	285414	4.03 (3.33, 4.84)
		MASLD+low PP-NO	11	18364	5.99 (2.99, 10.72)
		MASLD+low PP-O	38	80589	4.72 (3.34, 6.47)
		MASLD+low PP	49	98953	4.95 (3.66, 6.55)
	Non-DM	Healthy	89	3643569	0.24 (0.20, 0.30)
		MASLD+normal PP	94	1683700	0.56 (0.45, 0.68)
		MASLD+low PP-NO	14	59358	2.36 (1.29, 3.96)
		MASLD+low PP-O	14	147943	0.95 (0.52, 1.59)
		MASLD+low PP	28	207301	1.35 (0.90, 1.95)
CVD	DM	Healthy	2066	118413	174.47 (167.03, 182.16)
		MASLD+normal PP	4793	243177	197.10 (191.56, 202.76)
		MASLD+low PP-NO	417	12989	321.04 (290.96, 353.38)
		MASLD+low PP-O	1747	59692	292.67 (279.10, 306.72)
		MASLD+low PP	2164	72681	297.74 (285.33, 310.55)
	Non-DM	Healthy	16371	3512088	46.61 (45.90, 47.33)

		MASLD+normal PP	11648	1584134	73.53 (72.20, 74.88)
		MASLD+low PP-NO	824	48717	169.14 (157.79, 181.09)
		MASLD+low PP-O	1668	129684	128.62 (122.52, 134.94)
		MASLD+low PP	2492	178401	139.69 (134.25, 145.28)
CKD	DM	Healthy	1300	130495	99.62 (94.28, 105.19)
		MASLD+normal PP	3244	270525	119.91 (115.82, 124.11)
		MASLD+low PP-NO	306	16569	184.68 (164.57, 206.58)
		MASLD+low PP-O	1483	71884	206.30 (195.94, 217.08)
		MASLD+low PP	1789	88453	202.25 (192.99, 211.85)
	Non-DM	Healthy	6667	3614859	18.44 (18.00, 18.89)
		MASLD+normal PP	5200	1660605	31.31 (30.47, 32.18)
		MASLD+low PP-NO	381	57286	66.51 (60.00, 73.53)
		MASLD+low PP-O	1056	142237	74.24 (69.83, 78.86)
		MASLD+low PP	1437	199524	72.02 (68.35, 75.84)
Non-liver cancers	DM	Healthy	1512	123100	122.83 (116.71, 129.18)
		MASLD+normal PP	3272	257921	126.86 (122.55, 131.28)
		MASLD+low PP-NO	208	16193	128.45 (111.59, 147.14)
		MASLD+low PP-O	865	72328	119.59 (111.76, 127.84)
		MASLD+low PP	1073	88521	121.21 (114.07, 128.69)
	Non-DM	Healthy	19209	3464140	55.45 (54.67, 56.24)
		MASLD+normal PP	11604	1576419	73.61 (72.28, 74.96)
		MASLD+low PP-NO	532	53248	99.91 (91.60, 108.77)
		MASLD+low PP-O	1038	136501	76.04 (71.49, 80.81)
		MASLD+low PP	1570	189750	82.74 (78.70, 86.94)
Obesity-related cancers	DM	Healthy	527	131821	39.98 (36.64, 43.54)
		MASLD+normal PP	1321	275312	47.98 (45.43, 50.64)
		MASLD+low PP-NO	75	17737	42.28 (33.26, 53.00)
		MASLD+low PP-O	376	77346	48.61 (43.82, 53.78)
		MASLD+low PP	451	95083	47.43 (43.16, 52.02)
	Non-DM	Healthy	6051	3586670	16.87 (16.45, 17.30)
		MASLD+normal PP	3633	1650054	22.02 (21.31, 22.75)
		MASLD+low PP-NO	163	57677	28.26 (24.09, 32.95)
		MASLD+low PP-O	390	143762	27.13 (24.50, 29.96)
		MASLD+low PP	553	201439	27.45 (25.21, 29.84)

Table S17. Incidence of clinical outcomes according to categories of MASLD and muscle strength (MS) stratified by FIB-4.

**Outcomes**		**Groups**	**Incident cases**	**PYs**	**incidence (10,000 PYs)**
Death	FIB-4 <1.3	Healthy	5422	1812615	29.91 (29.12, 30.72)
		MASLD+normal MS	6884	1206560	57.05 (55.71, 58.42)
		MASLD+lowl MS-NO	238	23059	103.21 (90.52, 117.19)
		MASLD+low MS-O	449	41858	107.27 (97.57, 117.66)
		MASLD+low MS	687	64917	105.83 (98.06, 114.05)
	1.3≤ FIB-4≤2.67	Healthy	8216	1386253	59.27 (57.99, 60.56)
		MASLD+normal MS	7974	749623	106.37 (104.05, 108.73)
		MASLD+lowl MS-NO	326	18475	176.45 (157.82, 196.69)
		MASLD+low MS-O	556	29578	187.98 (172.68, 204.27)
		MASLD+low MS	882	48054	183.54 (171.63, 196.07)
	FIB-4 >2.67	Healthy	708	60259	117.49 (109.00, 126.48)
		MASLD+normal MS	919	40577	226.48 (212.07, 241.61)
		MASLD+lowl MS-NO	48	1345	356.88 (263.13, 473.17)
		MASLD+low MS-O	71	2273	312.36 (243.96, 394.00)
		MASLD+low MS	119	3618	328.91 (272.48, 393.59)
Cirrhosis	FIB-4 <1.3	Healthy	103	1840696	0.56 (0.46, 0.68)
		MASLD+normal MS	246	1240765	1.98 (1.74, 2.25)
		MASLD+lowl MS-NO	9	24246	3.71 (1.70, 7.05)
		MASLD+low MS-O	27	44007	6.14 (4.04, 8.93)
		MASLD+low MS	36	68253	5.27 (3.69, 7.30)
	1.3≤ FIB-4≤2.67	Healthy	171	1425844	1.20 (1.03, 1.39)
		MASLD+normal MS	527	785829	6.71 (6.15, 7.30)
		MASLD+lowl MS-NO	15	20061	7.48 (4.18, 12.33)
		MASLD+low MS-O	42	31966	13.14 (9.47, 17.76)
		MASLD+low MS	57	52027	10.96 (8.30, 14.19)
	FIB-4 >2.67	Healthy	70	63508	11.02 (8.59, 13.93)
		MASLD+normal MS	310	42824	72.39 (64.55, 80.91)
		MASLD+lowl MS-NO	16	1468	108.99 (62.30, 177.00)
		MASLD+low MS-O	28	2455	114.05 (75.79, 164.84)
		MASLD+low MS	44	3923	112.16 (81.49, 150.57)
HCC	FIB-4 <1.3	Healthy	31	1841057	0.17 (0.11, 0.24)
		MASLD+normal MS	31	1242053	0.25 (0.17, 0.35)
		MASLD+lowl MS-NO	3	24296	1.23 (0.25, 3.61)
		MASLD+low MS-O	4	44112	0.91 (0.25, 2.32)
		MASLD+low MS	7	68408	1.02 (0.41, 2.11)
	1.3≤ FIB-4≤2.67	Healthy	49	1426686	0.34 (0.25, 0.45)
		MASLD+normal MS	117	788387	1.48 (1.23, 1.78)

		MASLD+lowl MS-NO	5	20225	2.47 (0.80, 5.77)
		MASLD+low MS-O	6	32246	1.86 (0.68, 4.05)
		MASLD+low MS	11	52472	2.10 (1.05, 3.75)
	FIB-4 >2.67	Healthy	15	64182	2.34 (1.31, 3.85)
		MASLD+normal MS	93	45326	20.52 (16.56, 25.14)
		MASLD+lowl MS-NO	4	1630	24.54 (6.69, 62.83)
		MASLD+low MS-O	10	2644	37.82 (18.14, 69.56)
		MASLD+low MS	14	4273	32.76 (17.91, 54.97)
CVD	FIB-4 <1.3	Healthy	6290	1792640	35.09 (34.23, 35.97)
		MASLD+normal MS	8987	1163914	77.21 (75.63, 78.83)
		MASLD+lowl MS-NO	284	21524	131.95 (117.04, 148.22)
		MASLD+low MS-O	544	38755	140.37 (128.82, 152.68)
		MASLD+low MS	828	60278	137.36 (128.17, 147.05)
	1.3≤ FIB-4≤2.67	Healthy	8901	1353418	65.77 (64.41, 67.15)
		MASLD+normal MS	9054	700251	129.30 (126.65, 131.99)
		MASLD+lowl MS-NO	289	16972	170.28 (151.21, 191.09)
		MASLD+low MS-O	529	26699	198.13 (181.61, 215.76)
		MASLD+low MS	818	43672	187.31 (174.69, 200.59)
	FIB-4 >2.67	Healthy	613	58437	104.90 (96.76, 113.54)
		MASLD+normal MS	667	38543	173.05 (160.17, 186.70)
		MASLD+lowl MS-NO	29	1309	221.54 (148.37, 318.17)
		MASLD+low MS-O	50	2150	232.56 (172.61, 306.60)
		MASLD+low MS	79	3459	228.39 (180.82, 284.64)
CKD	FIB-4 <1.3	Healthy	2329	1831064	12.72 (12.21, 13.25)
		MASLD+normal MS	4396	1221353	35.99 (34.94, 37.07)
		MASLD+lowl MS-NO	166	23422	70.87 (60.50, 82.51)
		MASLD+low MS-O	387	42038	92.06 (83.11, 101.70)
		MASLD+low MS	553	65460	84.48 (77.58, 91.82)
	1.3≤ FIB-4≤2.67	Healthy	4116	1408636	29.22 (28.33, 30.13)
		MASLD+normal MS	5263	763242	68.96 (67.11, 70.84)
		MASLD+lowl MS-NO	160	19229	83.21 (70.81, 97.15)
		MASLD+low MS-O	412	29876	137.90 (124.91, 151.88)
		MASLD+low MS	572	49105	116.49 (107.13, 126.43)
	FIB-4 >2.67	Healthy	305	62791	48.57 (43.27, 54.34)
		MASLD+normal MS	458	43322	105.72 (96.26, 115.86)
		MASLD+lowl MS-NO	27	1469	183.80 (121.12, 267.42)
		MASLD+low MS-O	43	2438	176.37 (127.64, 237.57)
		MASLD+low MS	70	3907	179.17 (139.67, 226.36)
Non-liver cancers	FIB-4 <1.3	Healthy	7860	1770941	44.38 (43.41, 45.38)

		MASLD+normal MS	7845	1171804	66.95 (65.47, 68.45)
		MASLD+lowl MS-NO	160	22540	70.98 (60.41, 82.88)
		MASLD+low MS-O	326	41012	79.49 (71.09, 88.60)
		MASLD+low MS	486	63552	76.47 (69.82, 83.58)
	1.3≤ FIB-4≤2.67	Healthy	9545	1337125	71.38 (69.96, 72.83)
		MASLD+normal MS	7559	716220	105.54 (103.17, 107.95)
		MASLD+lowl MS-NO	193	18228	105.88 (91.47, 121.92)
		MASLD+low MS-O	309	29231	105.71 (94.25, 118.18)
		MASLD+low MS	502	47460	105.77 (96.72, 115.44)
	FIB-4 >2.67	Healthy	574	58254	98.53 (90.64, 106.93)
		MASLD+normal MS	538	40076	134.24 (123.14, 146.08)
		MASLD+lowl MS-NO	22	1423	154.60 (96.89, 234.07)
		MASLD+low MS-O	35	2322	150.73 (104.99, 209.63)
		MASLD+low MS	57	3746	152.16 (115.25, 197.14)
Obesity-related cancers	FIB-4 <1.3	Healthy	2552	1817901	14.04 (13.50, 14.59)
		MASLD+normal MS	2731	1217901	22.42 (21.59, 23.28)
		MASLD+lowl MS-NO	51	23815	21.42 (15.94, 28.16)
		MASLD+low MS-O	120	43005	27.90 (23.13, 33.37)
		MASLD+low MS	171	66819	25.59 (21.90, 29.73)
	1.3≤ FIB-4≤2.67	Healthy	2929	1398665	20.94 (20.19, 21.71)
		MASLD+normal MS	2423	765847	31.64 (30.39, 32.92)
		MASLD+lowl MS-NO	52	19714	26.38 (19.70, 34.59)
		MASLD+low MS-O	121	31191	38.79 (32.19, 46.35)
		MASLD+low MS	173	50905	33.98 (29.11, 39.44)
	FIB-4 >2.67	Healthy	186	62268	29.87 (25.73, 34.49)
		MASLD+normal MS	235	43548	53.96 (47.28, 61.32)
		MASLD+lowl MS-NO	10	1572	63.61 (30.51, 116.99)
		MASLD+low MS-O	19	2561	74.19 (44.67, 115.86)
		MASLD+low MS	29	4133	70.17 (46.99, 100.77)

Table S18. Incidence of clinical outcomes according to categories of MASLD and muscle mass (MM) stratified by FIB-4.

Outcomes		Groups	Incident cases	PYs	Incidence (10,000 PYs)
Death	FIB-4 <1.3	Healthy	5422	1812615	29.91 (29.12, 30.72)
		MASLD+normal MM	7518	1266787	59.35 (58.01, 60.70)
		MASLD+low MM-NO	40	3718	107.58 (76.86, 146.50)
		MASLD+low MM-O	13	971	133.88 (71.29, 228.94)
		MASLD+low MM	53	4689	113.03 (84.67, 147.85)
	1.3≤ FIB-4≤2.67	Healthy	8216	1386253	59.27 (57.99, 60.56)
		MASLD+normal MM	8811	794650	110.88 (108.58, 113.22)
		MASLD+low MM-NO	41	2575	159.22 (114.26, 216.00)
		MASLD+low MM-O	4	451	88.69 (24.17, 227.09)
		MASLD+low MM	45	3026	148.71 (108.47, 198.99)
	FIB-4 >2.67	Healthy	708	60259	117.49 (109.00, 126.48)
		MASLD+normal MM	1030	43929	234.47 (220.37, 249.24)
		MASLD+low MM-NO	4	240	166.67 (45.41, 426.73)
		MASLD+low MM-O	4	27	1481.48 (403.65, 3793.18)
		MASLD+low MM	8	267	299.63 (129.36, 590.38)
Cirrhosis	FIB-4 <1.3	Healthy	103	1840696	0.56 (0.46, 0.68)
		MASLD+normal MM	282	1304061	2.16 (1.92, 2.43)
		MASLD+low MM-NO	0	3923	—
		MASLD+low MM-O	0	1034	—
		MASLD+low MM	0	4957	—
	1.3≤ FIB-4≤2.67	Healthy	171	1425844	1.20 (1.03, 1.39)
		MASLD+normal MM	582	834611	6.97 (6.42, 7.56)
		MASLD+low MM-NO	2	2784	7.18 (0.87, 25.95)
		MASLD+low MM-O	0	461	—
		MASLD+low MM	2	3245	6.16 (0.75, 22.26)
	FIB-4 >2.67	Healthy	70	63508	11.02 (8.59, 13.93)
		MASLD+normal MM	352	46429	75.81 (68.10, 84.16)
		MASLD+low MM-NO	1	276	36.23 (0.92, 201.87)
		MASLD+low MM-O	1	43	232.56 (5.89, 1295.73)
		MASLD+low MM	2	318	62.89 (7.62, 227.19)
HCC	FIB-4 <1.3	Healthy	31	1841057	0.17 (0.11, 0.24)
		MASLD+normal MM	37	1305517	0.28 (0.20, 0.39)
		MASLD+low MM-NO	1	3910	2.56 (0.06, 14.25)
		MASLD+low MM-O	0	1034	—
		MASLD+low MM	1	4944	2.02 (0.05, 11.27)
	1.3≤ FIB-4≤2.67	Healthy	49	1426686	0.34 (0.25, 0.45)
		MASLD+normal MM	127	837602	1.52 (1.26, 1.80)

		MASLD+low MM-NO	1	2796	3.58 (0.09, 19.93)
		MASLD+low MM-O	0	461	—
		MASLD+low MM	1	3257	3.07 (0.08, 17.11)
	FIB-4 >2.67	Healthy	15	64182	2.34 (1.31, 3.85)
		MASLD+normal MM	107	49252	21.73 (17.80, 26.25)
		MASLD+low MM-NO	0	277	—
		MASLD+low MM-O	0	69	—
		MASLD+low MM	0	347	—
CVD	FIB-4 <1.3	Healthy	6290	1792640	35.09 (34.23, 35.97)
		MASLD+normal MM	9775	1219751	80.14 (78.56, 81.74)
		MASLD+low MM-NO	33	3515	93.88 (64.62, 131.85)
		MASLD+low MM-O	7	926	75.59 (30.39, 155.75)
		MASLD+low MM	40	4442	90.05 (64.33, 122.62)
	1.3≤ FIB-4≤2.67	Healthy	8901	1353418	65.77 (64.41, 67.15)
		MASLD+normal MM	9842	741026	132.82 (130.20, 135.47)
		MASLD+low MM-NO	25	2456	101.79 (65.87, 150.26)
		MASLD+low MM-O	5	440	113.64 (36.90, 265.19)
		MASLD+low MM	30	2896	103.59 (69.89, 147.88)
	FIB-4 >2.67	Healthy	613	58437	104.90 (96.76, 113.54)
		MASLD+normal MM	745	41687	178.71 (166.11, 192.02)
		MASLD+low MM-NO	1	260	38.46 (0.97, 214.29)
		MASLD+low MM-O	0	55	—
		MASLD+low MM	1	315	31.75 (0.80, 176.88)
CKD	FIB-4 <1.3	Healthy	2329	1831064	12.72 (12.21, 13.25)
		MASLD+normal MM	4925	1281950	38.42 (37.35, 39.51)
		MASLD+low MM-NO	21	3838	54.72 (33.87, 83.64)
		MASLD+low MM-O	3	1026	29.24 (6.03, 85.45)
		MASLD+low MM	24	4864	49.34 (31.61, 73.42)
	1.3≤ FIB-4≤2.67	Healthy	4116	1408636	29.22 (28.33, 30.13)
		MASLD+normal MM	5812	809242	71.82 (69.99, 73.69)
		MASLD+low MM-NO	21	2663	78.86 (48.81, 120.54)
		MASLD+low MM-O	2	442	45.25 (5.48, 163.45)
		MASLD+low MM	23	3105	74.07 (46.96, 111.15)
	FIB-4 >2.67	Healthy	305	62791	48.57 (43.27, 54.34)
		MASLD+normal MM	527	46896	112.38 (102.99, 122.39)
		MASLD+low MM-NO	1	263	38.02 (0.96, 211.85)
		MASLD+low MM-O	0	69	—
		MASLD+low MM	1	333	30.03 (0.76, 167.32)
Non-liver cancers	FIB-4 <1.3	Healthy	7860	1770941	44.38 (43.41, 45.38)

		MASLD+normal MM	8292	1230920	67.36 (65.92, 68.83)
		MASLD+low MM-NO	31	3516	88.17 (59.91, 125.15)
		MASLD+low MM-O	8	920	86.96 (37.54, 171.34)
		MASLD+low MM	39	4436	87.92 (62.52, 120.19)
	1.3≤ FIB-4≤2.67	Healthy	9545	1337125	71.38 (69.96, 72.83)
		MASLD+normal MM	8037	760750	105.65 (103.35, 107.98)
		MASLD+low MM-NO	22	2521	87.27 (54.69, 132.12)
		MASLD+low MM-O	2	409	48.90 (5.92, 176.64)
		MASLD+low MM	24	2930	81.91 (52.48, 121.88)
	FIB-4 >2.67	Healthy	574	58254	98.53 (90.64, 106.93)
		MASLD+normal MM	589	43525	135.32 (124.62, 146.71)
		MASLD+low MM-NO	4	249	160.64 (43.77, 411.31)
		MASLD+low MM-O	2	47	425.53 (51.53, 1537.17)
		MASLD+low MM	6	297	202.02 (74.14, 439.71)
Obesity-related cancers	FIB-4 <1.3	Healthy	2552	1817901	14.04 (13.50, 14.59)
		MASLD+normal MM	2886	1279961	22.55 (21.73, 23.39)
		MASLD+low MM-NO	13	3756	34.61 (18.43, 59.19)
		MASLD+low MM-O	3	1003	29.91 (6.17, 87.41)
		MASLD+low MM	16	4759	33.62 (19.22, 54.60)
	1.3≤ FIB-4≤2.67	Healthy	2929	1398665	20.94 (20.19, 21.71)
		MASLD+normal MM	2587	813597	31.80 (30.58, 33.05)
		MASLD+low MM-NO	7	2732	25.62 (10.30, 52.79)
		MASLD+low MM-O	2	423	47.28 (5.73, 170.80)
		MASLD+low MM	9	3155	28.53 (13.04, 54.15)
	FIB-4 >2.67	Healthy	186	62268	29.87 (25.73, 34.49)
		MASLD+normal MM	259	47374	54.67 (48.22, 61.75)
		MASLD+low MM-NO	4	249	160.64 (43.77, 411.31)
		MASLD+low MM-O	1	58	172.41 (4.37, 960.63)
		MASLD+low MM	5	307	162.87 (52.88, 380.08)

Table S19. Incidence of clinical outcomes according to categories of MASLD and physical performance (PP) stratified by FIB-4.

Outcomes		Groups	Incident cases	PYs	Incidence (10,000 PYs)
Death	FIB-4 <1.3	Healthy	5422	1812615	29.91 (29.12, 30.72)
		MASLD+normal PP	5560	1113140	49.95 (48.64, 51.28)
		MASLD+low PP-NO	534	37665	141.78 (130.00, 154.33)
		MASLD+low PP-O	1477	120671	122.40 (116.24, 128.80)
		MASLD+low PP	2011	158336	127.01 (121.52, 132.68)
	1.3≤ FIB-4≤2.67	Healthy	8216	1386253	59.27 (57.99, 60.56)
		MASLD+normal PP	6602	691344	95.50 (93.21, 97.83)
		MASLD+low PP-NO	662	28216	234.62 (217.08, 253.19)
		MASLD+low PP-O	1592	78116	203.80 (193.91, 214.06)
		MASLD+low PP	2254	106332	211.98 (203.32, 220.91)
	FIB-4 >2.67	Healthy	708	60259	117.49 (109.00, 126.48)
		MASLD+normal PP	745	36817	202.35 (188.08, 217.42)
		MASLD+low PP-NO	107	2229	480.04 (393.40, 580.07)
		MASLD+low PP-O	186	5150	361.17 (311.12, 416.96)
		MASLD+low PP	293	7379	397.07 (352.90, 445.24)
Cirrhosis	FIB-4 <1.3	Healthy	103	1840696	0.56 (0.46, 0.68)
		MASLD+normal PP	197	1140489	1.73 (1.49, 1.99)
		MASLD+low PP-NO	17	40565	4.19 (2.44, 6.71)
		MASLD+low PP-O	68	127964	5.31 (4.13, 6.74)
		MASLD+low PP	85	168529	5.04 (4.03, 6.24)
	1.3≤ FIB-4≤2.67	Healthy	171	1425844	1.20 (1.03, 1.39)
		MASLD+normal PP	418	721003	5.80 (5.25, 6.38)
		MASLD+low PP-NO	37	31622	11.70 (8.24, 16.13)
		MASLD+low PP-O	129	85232	15.14 (12.64, 17.98)
		MASLD+low PP	166	116853	14.21 (12.13, 16.54)
	FIB-4 >2.67	Healthy	70	63508	11.02 (8.59, 13.93)
		MASLD+normal PP	260	38582	67.39 (59.45, 76.10)
		MASLD+low PP-NO	25	2578	96.97 (62.76, 143.15)
		MASLD+low PP-O	69	5587	123.50 (96.09, 156.30)
		MASLD+low PP	94	8165	115.13 (93.03, 140.88)
HCC	FIB-4 <1.3	Healthy	31	1841057	0.17 (0.11, 0.24)
		MASLD+normal PP	30	1141339	0.26 (0.18, 0.38)
		MASLD+low PP-NO	4	40684	0.98 (0.27, 2.52)
		MASLD+low PP-O	4	128437	0.31 (0.08, 0.80)
		MASLD+low PP	8	169122	0.47 (0.20, 0.93)
	1.3≤ FIB-4≤2.67	Healthy	49	1426686	0.34 (0.25, 0.45)
		MASLD+normal PP	85	722988	1.18 (0.94, 1.45)

		MASLD+low PP-NO	11	31868	3.45 (1.72, 6.18)
		MASLD+low PP-O	32	86003	3.72 (2.55, 5.25)
		MASLD+low PP	43	117870	3.65 (2.64, 4.91)
	FIB-4 >2.67	Healthy	15	64182	2.34 (1.31, 3.85)
		MASLD+normal PP	85	40496	20.99 (16.77, 25.95)
		MASLD+low PP-NO	9	2878	31.27 (14.30, 59.36)
		MASLD+low PP-O	13	6225	20.88 (11.12, 35.71)
		MASLD+low PP	22	9103	24.17 (15.15, 36.59)
CVD	FIB-4 <1.3	Healthy	6290	1792640	35.09 (34.23, 35.97)
		MASLD+normal PP	7646	1079847	70.81 (69.23, 72.41)
		MASLD+low PP-NO	562	33898	165.79 (152.37, 180.08)
		MASLD+low PP-O	1607	110447	145.50 (138.47, 152.79)
		MASLD+low PP	2169	144345	150.26 (144.01, 156.72)
	1.3≤ FIB-4≤2.67	Healthy	8901	1353418	65.77 (64.41, 67.15)
		MASLD+normal PP	7738	652267	118.63 (116.00, 121.31)
		MASLD+low PP-NO	582	23854	243.98 (224.56, 264.64)
		MASLD+low PP-O	1552	67802	228.90 (217.65, 240.58)
		MASLD+low PP	2134	91656	232.83 (223.05, 242.92)
	FIB-4 >2.67	Healthy	613	58437	104.90 (96.76, 113.54)
		MASLD+normal PP	571	35211	162.17 (149.14, 176.03)
		MASLD+low PP-NO	53	2139	247.78 (185.60, 324.10)
		MASLD+low PP-O	122	4652	262.25 (217.79, 313.13)
		MASLD+low PP	175	6791	257.69 (220.93, 298.83)
CKD	FIB-4 <1.3	Healthy	2329	1831064	12.72 (12.21, 13.25)
		MASLD+normal PP	3523	1125593	31.30 (30.27, 32.35)
		MASLD+low PP-NO	291	39147	74.34 (66.04, 83.39)
		MASLD+low PP-O	1135	122073	92.98 (87.65, 98.55)
		MASLD+low PP	1426	161220	88.45 (83.92, 93.16)
	1.3≤ FIB-4≤2.67	Healthy	4116	1408636	29.22 (28.33, 30.13)
		MASLD+normal PP	4312	703192	61.32 (59.50, 63.18)
		MASLD+low PP-NO	329	29957	109.82 (98.28, 122.36)
		MASLD+low PP-O	1194	79199	150.76 (142.33, 159.56)
		MASLD+low PP	1523	109156	139.53 (132.60, 146.71)
	FIB-4 >2.67	Healthy	305	62791	48.57 (43.27, 54.34)
		MASLD+normal PP	370	39058	94.73 (85.32, 104.89)
		MASLD+low PP-NO	40	2629	152.15 (108.70, 207.18)
		MASLD+low PP-O	118	5541	212.96 (176.27, 255.03)
		MASLD+low PP	158	8170	193.39 (164.41, 226.01)
Non-liver cancers	FIB-4 <1.3	Healthy	7860	1770941	44.38 (43.41, 45.38)

		MASLD+normal PP	7044	1079635	65.24 (63.73, 66.79)
		MASLD+low PP-NO	330	36961	89.28 (79.91, 99.46)
		MASLD+low PP-O	957	118760	80.58 (75.56, 85.85)
		MASLD+low PP	1287	155721	82.65 (78.19, 87.29)
	1.3≤ FIB-4≤2.67	Healthy	9545	1337125	71.38 (69.96, 72.83)
		MASLD+normal PP	6902	658263	104.85 (102.39, 107.36)
		MASLD+low PP-NO	343	28129	121.94 (109.37, 135.55)
		MASLD+low PP-O	816	77288	105.58 (98.46, 113.08)
		MASLD+low PP	1159	105417	109.94 (103.70, 116.46)
	FIB-4 >2.67	Healthy	574	58254	98.53 (90.64, 106.93)
		MASLD+normal PP	484	35990	134.48 (122.77, 147.01)
		MASLD+low PP-NO	43	2338	183.92 (133.10, 247.74)
		MASLD+low PP-O	68	5494	123.77 (96.11, 156.91)
		MASLD+low PP	111	7832	141.73 (116.59, 170.67)
Obesity-related cancers	FIB-4 <1.3	Healthy	2552	1817901	14.04 (13.50, 14.59)
		MASLD+normal PP	2405	1120273	21.47 (20.62, 22.34)
		MASLD+low PP-NO	102	39697	25.69 (20.95, 31.19)
		MASLD+low PP-O	395	124750	31.66 (28.62, 34.95)
		MASLD+low PP	497	164447	30.22 (27.62, 33.00)
	1.3≤ FIB-4≤2.67	Healthy	2929	1398665	20.94 (20.19, 21.71)
		MASLD+normal PP	2177	703075	30.96 (29.68, 32.29)
		MASLD+low PP-NO	106	30844	34.37 (28.14, 41.57)
		MASLD+low PP-O	313	82832	37.79 (33.72, 42.21)
		MASLD+low PP	419	113677	36.86 (33.41, 40.56)
	FIB-4 >2.67	Healthy	186	62268	29.87 (25.73, 34.49)
		MASLD+normal PP	213	39044	54.55 (47.47, 62.39)
		MASLD+low PP-NO	20	2706	73.91 (45.15, 114.15)
		MASLD+low PP-O	31	5931	52.27 (35.51, 74.19)
		MASLD+low PP	51	8637	59.05 (43.97, 77.64)

Table S20. Incidence of clinical outcomes according to MASLD and muscle strength (MS) categories in White participants (sensitivity analysis).

Outcomes	Groups	Incident cases	PYs	incidence (10,000 PYs)
Death	Healthy	16200	3507724	46.18 (45.48, 46.90)
	MASLD+normal MS	15700	1949324	80.54 (79.29, 81.81)
	MASLD+lowl MS-NO	581	38386	151.36 (139.30, 164.18)
	MASLD+low MS-O	1032	68650	150.33 (141.29, 159.79)
	MASLD+low MS	1613	107036	150.70 (143.43, 158.23)
Cirrhosis	Healthy	429	3587797	1.20 (1.09, 1.31)
	MASLD+normal MS	1062	2022158	5.25 (4.94, 5.58)
	MASLD+lowl MS-NO	39	41151	9.48 (6.74, 12.96)
	MASLD+low MS-O	95	73172	12.98 (10.50, 15.87)
	MASLD+low MS	134	114323	11.72 (9.82, 13.88)
HCC	Healthy	109	3590206	0.30 (0.25, 0.37)
	MASLD+normal MS	239	2028401	1.18 (1.03, 1.34)
	MASLD+lowl MS-NO	13	41494	3.13 (1.67, 5.36)
	MASLD+low MS-O	20	73715	2.71 (1.66, 4.19)
	MASLD+low MS	33	115209	2.86 (1.97, 4.02)
CVD	Healthy	17631	3447390	51.14 (50.39, 51.90)
	MASLD+normal MS	18410	1857610	99.11 (97.68, 100.55)
	MASLD+lowl MS-NO	546	35790	152.56 (140.03, 165.91)
	MASLD+low MS-O	1062	62969	168.65 (158.66, 179.11)
	MASLD+low MS	1608	98759	162.82 (154.96, 170.98)
CKD	Healthy	7539	3557593	21.19 (20.72, 21.68)
	MASLD+normal MS	9844	1982177	49.66 (48.69, 50.65)
	MASLD+lowl MS-NO	324	39735	81.54 (72.90, 90.92)
	MASLD+low MS-O	796	69424	114.66 (106.83, 122.91)
	MASLD+low MS	1120	109159	102.60 (96.68, 108.79)
Non-liver cancers	Healthy	19958	3404684	58.62 (57.81, 59.44)
	MASLD+normal MS	15805	1881787	83.99 (82.68, 85.31)
	MASLD+lowl MS-NO	351	37777	92.91 (83.45, 103.16)
	MASLD+low MS-O	656	67510	97.17 (89.88, 104.90)
	MASLD+low MS	1007	105288	95.64 (89.83, 101.74)
Obesity-related cancers	Healthy	6341	3531025	17.96 (17.52, 18.41)
	MASLD+normal MS	5356	1980081	27.05 (26.33, 27.78)
	MASLD+lowl MS-NO	111	40469	27.43 (22.56, 33.03)
	MASLD+low MS-O	258	71530	36.07 (31.80, 40.75)
	MASLD+low MS	369	111999	32.95 (29.67, 36.49)

Table S21. Incidence of clinical outcomes according to MASLD and muscle mass (MM) categories in White participants (sensitivity analysis).

Outcomes	Groups	Incident cases	PYs	incidence (10,000 PYs)
Death	Healthy	16200	3507724	46.18 (45.48, 46.90)
	MASLD+normal MM	17211	2048687	84.01 (82.76, 85.27)
	MASLD+low MM-NO	81	6266	129.27 (102.66, 160.67)
	MASLD+low MM-O	21	1407	149.25 (92.39, 228.15)
	MASLD+low MM	102	7673	132.93 (108.39, 161.37)
Cirrhosis	Healthy	429	3587797	1.20 (1.09, 1.31)
	MASLD+normal MM	1191	2128303	5.60 (5.28, 5.92)
	MASLD+low MM-NO	4	6683	5.99 (1.63, 15.32)
	MASLD+low MM-O	1	1495	6.69 (0.17, 37.27)
	MASLD+low MM	5	8178	6.11 (1.99, 14.27)
HCC	Healthy	109	3590206	0.30 (0.25, 0.37)
	MASLD+normal MM	270	2135393	1.26 (1.12, 1.42)
	MASLD+low MM-NO	2	6695	2.99 (0.36, 10.79)
	MASLD+low MM-O	0	1522	—
	MASLD+low MM	2	8217	2.43 (0.29, 8.79)
CVD	Healthy	17631	3447390	51.14 (50.39, 51.90)
	MASLD+normal MM	19949	1949026	102.35 (100.94, 103.78)
	MASLD+low MM-NO	57	5972	95.45 (72.29, 123.66)
	MASLD+low MM-O	12	1371	87.53 (45.23, 152.89)
	MASLD+low MM	69	7343	93.97 (73.11, 118.92)
CKD	Healthy	7539	3557593	21.19 (20.72, 21.68)
	MASLD+normal MM	10917	2083337	52.40 (51.42, 53.39)
	MASLD+low MM-NO	43	6501	66.14 (47.87, 89.10)
	MASLD+low MM-O	4	1499	26.68 (7.27, 68.32)
	MASLD+low MM	47	7999	58.76 (43.17, 78.13)
Non-liver cancers	Healthy	19958	3404684	58.62 (57.81, 59.44)
	MASLD+normal MM	16744	1979729	84.58 (83.30, 85.87)
	MASLD+low MM-NO	56	6011	93.16 (70.37, 120.98)
	MASLD+low MM-O	12	1334	89.96 (46.48, 157.13)
	MASLD+low MM	68	7345	92.58 (71.89, 117.37)
Obesity-related cancers	Healthy	6341	3531025	17.96 (17.52, 18.41)
	MASLD+normal MM	5696	2084188	27.33 (26.62, 28.05)
	MASLD+low MM-NO	23	6450	35.66 (22.60, 53.51)
	MASLD+low MM-O	6	1441	41.64 (15.28, 90.63)
	MASLD+low MM	29	7891	36.75 (24.61, 52.78)

Table S22. Incidence of clinical outcomes according to MASLD and physical performance (PP) categories in White participants (sensitivity analysis).

Outcomes	Groups	Incident cases	PYs	Incidence (10,000 PYs)
Death	Healthy	16200	3507724	46.18 (45.48, 46.90)
	MASLD+normal PP	12842	1802135	71.26 (70.03, 72.50)
	MASLD+low PP-NO	1281	61746	207.46 (196.26, 219.14)
	MASLD+low PP-O	3190	192479	165.73 (160.03, 171.59)
	MASLD+low PP	4471	254224	175.87 (170.75, 181.10)
Cirrhosis	Healthy	429	3587797	1.20 (1.09, 1.31)
	MASLD+normal PP	862	1860962	4.63 (4.33, 4.95)
	MASLD+low PP-NO	74	68473	10.81 (8.49, 13.57)
	MASLD+low PP-O	260	207045	12.56 (11.08, 14.18)
	MASLD+low PP	334	275518	12.12 (10.86, 13.49)
HCC	Healthy	109	3590206	0.30 (0.25, 0.37)
	MASLD+normal PP	198	1865661	1.06 (0.92, 1.22)
	MASLD+low PP-NO	24	69069	3.47 (2.23, 5.17)
	MASLD+low PP-O	50	208880	2.39 (1.78, 3.16)
	MASLD+low PP	74	277948	2.66 (2.09, 3.34)
CVD	Healthy	17631	3447390	51.14 (50.39, 51.90)
	MASLD+normal PP	15700	1729923	90.76 (89.34, 92.19)
	MASLD+low PP-NO	1143	54244	210.71 (198.67, 223.29)
	MASLD+low PP-O	3175	172202	184.38 (178.02, 190.90)
	MASLD+low PP	4318	226446	190.69 (185.04, 196.46)
CKD	Healthy	7539	3557593	21.19 (20.72, 21.68)
	MASLD+normal PP	7997	1830016	43.70 (42.75, 44.67)
	MASLD+low PP-NO	622	65672	94.71 (87.42, 102.46)
	MASLD+low PP-O	2345	195649	119.86 (115.05, 124.81)
	MASLD+low PP	2967	261321	113.54 (109.49, 117.70)
Non-liver cancers	Healthy	19958	3404684	58.62 (57.81, 59.44)
	MASLD+normal PP	14339	1735472	82.62 (81.28, 83.99)
	MASLD+low PP-NO	677	61372	110.31 (102.16, 118.94)
	MASLD+low PP-O	1796	190231	94.41 (90.10, 98.88)
	MASLD+low PP	2473	251603	98.29 (94.45, 102.24)
Obesity-related cancers	Healthy	6341	3531025	17.96 (17.52, 18.41)
	MASLD+normal PP	4786	1823307	26.25 (25.51, 27.00)
	MASLD+low PP-NO	219	66896	32.74 (28.54, 37.37)
	MASLD+low PP-O	720	201877	35.67 (33.11, 38.37)
	MASLD+low PP	939	268772	34.94 (32.74, 37.24)

Table S23. Association between combined MASLD and muscle strength (MS) status and clinical outcomes, stratified by age.

			Model1		Model2		Model3	
**Outcomes**		**Groups**	**HR (95% CI)**	**P value**	**HR (95% CI)**	**P value**	**HR (95% CI)**	**P value**
Death	Younger	Healthy	Ref		Ref		Ref	
		MASLD+normal MS	1.44 (1.37, 1.51)	<0.001	1.03 (0.98, 1.08)	0.297	1.05 (0.98, 1.12)	0.162
		MASLD+low MS-NO	2.20 (1.78, 2.72)	<0.001	1.19 (0.96, 1.47)	0.121	1.08 (0.84, 1.38)	0.544
		MASLD+low MS-O	2.69 (2.32, 3.11)	<0.001	1.00 (0.86, 1.16)	0.988	1.05 (0.89, 1.24)	0.555
		MASLD+low MS	2.51 (2.22, 2.84)	<0.001	1.05 (0.93, 1.19)	0.435	1.06 (0.92, 1.22)	0.427
	Older	Healthy	Ref		Ref		Ref	
		MASLD+normal MS	1.35 (1.32, 1.39)	<0.001	1.00 (0.98, 1.03)	0.798	1.03 (1.00, 1.06)	0.095
		MASLD+low MS-NO	1.83 (1.68, 2.00)	<0.001	1.18 (1.08, 1.28)	<0.001	1.20 (1.09, 1.32)	<0.001
		MASLD+low MS-O	2.03 (1.90, 2.17)	<0.001	1.10 (1.03, 1.18)	0.005	1.09 (1.01, 1.17)	0.033
		MASLD+low MS	1.95 (1.85, 2.06)	<0.001	1.13 (1.07, 1.19)	<0.001	1.12 (1.06, 1.20)	<0.001
Cirrhosis	Younger	Healthy	Ref		Ref		Ref	
		MASLD+normal MS	2.94 (2.47, 3.50)	<0.001	3.44 (2.85, 4.16)	<0.001	2.97 (2.33, 3.78)	<0.001
		MASLD+low MS-NO	5.58 (3.23, 9.65)	<0.001	6.67 (3.83, 11.60)	<0.001	1.73 (0.80, 3.76)	0.166
		MASLD+low MS-O	5.92 (3.91, 8.95)	<0.001	7.02 (4.58, 10.77)	<0.001	5.40 (3.28, 8.91)	<0.001
		MASLD+low MS	5.80 (4.10, 8.19)	<0.001	6.89 (4.82, 9.86)	<0.001	3.63 (2.28, 5.79)	<0.001
	Older	Healthy	Ref		Ref		Ref	
		MASLD+normal MS	3.72 (3.20, 4.33)	<0.001	4.29 (3.63, 5.08)	<0.001	3.59 (2.92, 4.41)	<0.001
		MASLD+low MS-NO	4.87 (3.28, 7.22)	<0.001	5.34 (3.53, 8.08)	<0.001	3.72 (2.30, 6.02)	<0.001
		MASLD+low MS-O	8.36 (6.43, 10.87)	<0.001	8.98 (6.76, 11.92)	<0.001	6.28 (4.55, 8.67)	<0.001
		MASLD+low MS	7.01 (5.54, 8.87)	<0.001	7.55 (5.85, 9.73)	<0.001	5.35 (3.99, 7.18)	<0.001
HCC	Younger	Healthy	Ref		Ref		Ref	
		MASLD+normal MS	1.82 (1.17, 2.84)	0.008	1.92 (1.20, 3.10)	0.007	1.81 (0.97, 3.35)	0.060
		MASLD+low MS-NO	3.81 (0.90, 16.09)	0.068	4.14 (0.97, 17.72)	0.055	0.77 (0.08, 7.81)	0.828
		MASLD+low MS-O	4.90 (1.72, 14.02)	0.003	4.01 (1.20, 13.39)	0.024	4.67 (1.29, 16.86)	0.019
		MASLD+low MS	4.48 (1.85, 10.85)	0.001	4.06 (1.54, 10.72)	0.005	2.52 (0.73, 8.67)	0.142
	Older	Healthy	Ref		Ref		Ref	
		MASLD+normal MS	3.10 (2.37, 4.07)	<0.001	3.13 (2.44, 4.01)	<0.001	1.77 (1.36, 2.29)	<0.001
		MASLD+low MS-NO	5.31 (2.81, 10.07)	<0.001	5.11 (2.72, 9.60)	<0.001	2.91 (1.54, 5.47)	0.001
		MASLD+low MS-O	5.97 (3.50, 10.17)	<0.001	6.01 (3.68, 9.81)	<0.001	2.98 (1.82, 4.88)	<0.001
		MASLD+low MS	5.69 (3.66, 8.85)	<0.001	5.63 (3.79, 8.37)	<0.001	2.95 (1.98, 4.40)	<0.001

CVD	Younger	Healthy	Ref		Ref		Ref	
		MASLD+normal MS	1.69 (1.62, 1.76)	<0.001	1.07 (1.02, 1.12)	0.003	0.95 (0.90, 1.00)	0.061
		MASLD+low MS-NO	2.22 (1.84, 2.68)	<0.001	1.49 (1.24, 1.80)	<0.001	1.27 (1.03, 1.57)	0.023
		MASLD+low MS-O	2.94 (2.58, 3.35)	<0.001	1.33 (1.16, 1.53)	<0.001	1.16 (1.00, 1.35)	0.049
		MASLD+low MS	2.66 (2.39, 2.97)	<0.001	1.38 (1.23, 1.55)	<0.001	1.20 (1.05, 1.36)	0.006
	Older	Healthy	Ref		Ref		Ref	
		MASLD+normal MS	1.45 (1.41, 1.48)	<0.001	1.06 (1.04, 1.09)	<0.001	1.02 (0.99, 1.06)	0.180
		MASLD+low MS-NO	1.71 (1.56, 1.87)	<0.001	1.25 (1.14, 1.36)	<0.001	1.18 (1.07, 1.30)	0.001
		MASLD+low MS-O	2.16 (2.02, 2.31)	<0.001	1.32 (1.23, 1.42)	<0.001	1.22 (1.13, 1.32)	<0.001
		MASLD+low MS	1.98 (1.87, 2.09)	<0.001	1.29 (1.22, 1.37)	<0.001	1.21 (1.13, 1.29)	<0.001
CKD	Younger	Healthy	Ref		Ref		Ref	
		MASLD+normal MS	2.24 (2.09, 2.40)	<0.001	1.24 (1.15, 1.34)	<0.001	1.10 (1.00, 1.22)	0.047
		MASLD+low MS-NO	2.97 (2.21, 3.99)	<0.001	1.49 (1.09, 2.02)	0.012	1.21 (0.85, 1.71)	0.283
		MASLD+low MS-O	4.58 (3.82, 5.48)	<0.001	1.66 (1.37, 2.01)	<0.001	1.25 (1.01, 1.55)	0.042
		MASLD+low MS	4.02 (3.43, 4.71)	<0.001	1.61 (1.36, 1.91)	<0.001	1.24 (1.02, 1.50)	0.029
	Older	Healthy	Ref		Ref		Ref	
		MASLD+normal MS	1.96 (1.89, 2.02)	<0.001	1.33 (1.28, 1.38)	<0.001	1.22 (1.16, 1.27)	<0.001
		MASLD+low MS-NO	2.27 (2.03, 2.54)	<0.001	1.52 (1.35, 1.70)	<0.001	1.37 (1.21, 1.56)	<0.001
		MASLD+low MS-O	3.29 (3.04, 3.55)	<0.001	1.78 (1.64, 1.93)	<0.001	1.48 (1.35, 1.62)	<0.001
		MASLD+low MS	2.89 (2.71, 3.09)	<0.001	1.69 (1.58, 1.81)	<0.001	1.44 (1.34, 1.56)	<0.001
Non-liver cancers	Younger	Healthy	Ref		Ref		Ref	
		MASLD+normal MS	1.17 (1.13, 1.22)	<0.001	1.18 (1.14, 1.23)	<0.001	1.12 (1.06, 1.18)	<0.001
		MASLD+low MS-NO	0.93 (0.71, 1.21)	0.586	0.93 (0.71, 1.22)	0.593	0.84 (0.63, 1.13)	0.259
		MASLD+low MS-O	1.34 (1.13, 1.59)	0.001	1.36 (1.15, 1.62)	<0.001	1.25 (1.04, 1.51)	0.019
		MASLD+low MS	1.19 (1.03, 1.37)	0.020	1.20 (1.04, 1.39)	0.015	1.10 (0.94, 1.30)	0.236
	Older	Healthy	Ref		Ref		Ref	
		MASLD+normal MS	1.13 (1.11, 1.16)	<0.001	1.14 (1.11, 1.17)	<0.001	1.10 (1.06, 1.14)	<0.001
		MASLD+low MS-NO	1.03 (0.93, 1.15)	0.550	1.05 (0.94, 1.17)	0.414	0.96 (0.84, 1.08)	0.476
		MASLD+low MS-O	1.23 (1.13, 1.33)	<0.001	1.22 (1.12, 1.34)	<0.001	1.12 (1.02, 1.24)	0.019
		MASLD+low MS	1.15 (1.07, 1.23)	<0.001	1.15 (1.07, 1.24)	<0.001	1.05 (0.98, 1.14)	0.183
Obesity-related cancers	Younger	Healthy	Ref		Ref		Ref	

	MASLD+normal MS	1.45 (1.36, 1.56)	<0.001	1.48 (1.38, 1.59)	<0.001	1.32 (1.20, 1.45)	<0.001
	MASLD+low MS-NO	1.26 (0.82, 1.94)	0.291	1.32 (0.86, 2.04)	0.203	1.18 (0.75, 1.87)	0.480
	MASLD+low MS-O	1.78 (1.36, 2.31)	<0.001	1.80 (1.37, 2.36)	<0.001	1.48 (1.10, 1.99)	0.009
	MASLD+low MS	1.60 (1.27, 2.01)	<0.001	1.64 (1.30, 2.06)	<0.001	1.38 (1.07, 1.79)	0.013
Older	Healthy	Ref		Ref		Ref	
	MASLD+normal MS	1.36 (1.31, 1.43)	<0.001	1.38 (1.32, 1.45)	<0.001	1.25 (1.18, 1.32)	<0.001
	MASLD+low MS-NO	1.05 (0.86, 1.29)	0.638	1.06 (0.86, 1.30)	0.614	0.89 (0.70, 1.12)	0.308
	MASLD+low MS-O	1.46 (1.27, 1.67)	<0.001	1.48 (1.29, 1.71)	<0.001	1.26 (1.08, 1.47)	0.004
	MASLD+low MS	1.30 (1.16, 1.46)	<0.001	1.32 (1.17, 1.49)	<0.001	1.12 (0.98, 1.28)	0.105

Table S24. Association between combined MASLD and muscle mass (MM) status and clinical outcomes, stratified by age.

Outcomes		Groups	Model1		Model2		Model3	
			HR (95% CI)	P value	HR (95% CI)	P value	HR (95% CI)	P value
Death	Younger	Healthy	Ref		Ref		Ref	
		MASLD+normal MM	1.48 (1.41, 1.55)	<0.001	1.03 (0.98, 1.08)	0.276	1.05 (0.98, 1.12)	0.150
		MASLD+low MM-NO	3.34 (1.85, 6.03)	<0.001	2.30 (1.27, 4.16)	0.006	1.98 (1.03, 3.83)	0.041
		MASLD+low MM-O	2.20 (0.55, 8.79)	0.266	0.45 (0.11, 1.80)	0.258	0.48 (0.12, 1.93)	0.301
		MASLD+low MM	3.09 (1.79, 5.33)	<0.001	1.41 (0.82, 2.43)	0.218	1.27 (0.70, 2.29)	0.438
	Older	Healthy	Ref		Ref		Ref	
		MASLD+normal MM	1.39 (1.36, 1.43)	<0.001	1.01 (0.99, 1.04)	0.327	1.03 (1.00, 1.07)	0.042
		MASLD+low MM-NO	1.78 (1.42, 2.23)	<0.001	1.15 (0.91, 1.44)	0.239	1.18 (0.92, 1.51)	0.188
		MASLD+low MM-O	1.91 (1.23, 2.97)	0.004	1.73 (1.12, 2.69)	0.014	1.52 (0.92, 2.53)	0.105
		MASLD+low MM	1.80 (1.48, 2.21)	<0.001	1.23 (1.01, 1.51)	0.042	1.23 (0.99, 1.54)	0.066
Cirrhosis	Younger	Healthy	Ref		Ref		Ref	
		MASLD+normal MM	3.08 (2.59, 3.65)	<0.001	3.61 (2.99, 4.35)	<0.001	3.08 (2.42, 3.92)	<0.001
		MASLD+low MM-NO	—	—	—	—	—	—
		MASLD+low MM-O	—	—	—	—	—	—
		MASLD+low MM	—	—	—	—	—	—
	Older	Healthy	Ref		Ref		Ref	
		MASLD+normal MM	3.96 (3.41, 4.60)	<0.001	4.54 (3.84, 5.36)	<0.001	3.72 (3.03, 4.56)	<0.001
		MASLD+low MM-NO	5.18 (1.93, 13.95)	0.001	4.43 (1.41, 13.89)	0.011	1.33 (0.32, 5.47)	0.692
		MASLD+low MM-O	5.55 (0.78, 39.63)	0.088	6.83 (0.95, 48.81)	0.056	—	—
		MASLD+low MM	5.25 (2.16, 12.76)	<0.001	4.86 (1.80, 13.11)	0.002	1.23 (0.30, 5.03)	0.777
HCC	Younger	Healthy	Ref		Ref		Ref	
		MASLD+normal MM	1.94 (1.26, 3.00)	0.003	2.03 (1.27, 3.24)	0.003	1.93 (1.05, 3.53)	0.034
		MASLD+low MM-NO	—	—	—	—	—	—
		MASLD+low MM-O	—	—	—	—	—	—
		MASLD+low MM	—	—	—	—	—	—
	Older	Healthy	Ref		Ref		Ref	
		MASLD+normal MM	3.26 (2.50, 4.26)	<0.001	3.28 (2.50, 4.32)	<0.001	1.83 (1.37, 2.44)	<0.001
		MASLD+low MM-NO	10.05 (2.45, 41.15)	0.001	10.01 (2.49, 40.22)	0.001	3.34 (0.83, 13.44)	0.089
		MASLD+low MM-O	—	—	—	—	—	—
		MASLD+low MM	8.24 (2.01, 33.77)	0.003	8.30 (2.07, 33.36)	0.003	3.09 (0.77, 12.42)	0.112

CVD	Younger	Healthy	Ref		Ref		Ref	
		MASLD+normal MM	1.73 (1.66, 1.80)	<0.001	1.08 (1.04, 1.13)	<0.001	0.96 (0.90, 1.01)	0.125
		MASLD+low MM-NO	0.79 (0.25, 2.45)	0.682	0.81 (0.26, 2.50)	0.708	0.67 (0.21, 2.08)	0.487
		MASLD+low MM-O	1.03 (0.15, 7.34)	0.974	0.60 (0.08, 4.24)	0.607	0.51 (0.07, 3.64)	0.503
		MASLD+low MM	0.84 (0.31, 2.24)	0.725	0.74 (0.28, 1.98)	0.549	0.62 (0.23, 1.66)	0.342
	Older	Healthy	Ref		Ref		Ref	
		MASLD+normal MM	1.49 (1.45, 1.52)	<0.001	1.08 (1.05, 1.11)	<0.001	1.04 (1.00, 1.07)	0.030
		MASLD+low MM-NO	1.42 (1.10, 1.84)	0.007	1.10 (0.85, 1.43)	0.467	1.06 (0.80, 1.41)	0.673
		MASLD+low MM-O	1.53 (0.92, 2.55)	0.098	0.95 (0.52, 1.71)	0.853	0.95 (0.51, 1.77)	0.869
		MASLD+low MM	1.45 (1.15, 1.82)	0.002	1.07 (0.84, 1.37)	0.565	1.04 (0.80, 1.35)	0.758
CKD	Younger	Healthy	Ref		Ref		Ref	
		MASLD+normal MM	2.33 (2.17, 2.50)	<0.001	1.26 (1.17, 1.36)	<0.001	1.11 (1.01, 1.23)	0.035
		MASLD+low MM-NO	1.79 (0.58, 5.56)	0.314	1.02 (0.26, 4.10)	0.974	0.90 (0.22, 3.63)	0.882
		MASLD+low MM-O	2.34 (0.33, 16.62)	0.396	1.37 (0.19, 9.73)	0.754	1.29 (0.18, 9.15)	0.801
		MASLD+low MM	1.90 (0.71, 5.07)	0.200	1.12 (0.36, 3.47)	0.848	1.00 (0.32, 3.12)	1.000
	Older	Healthy	Ref		Ref		Ref	
		MASLD+normal MM	2.04 (1.97, 2.10)	<0.001	1.36 (1.32, 1.41)	<0.001	1.24 (1.19, 1.30)	<0.001
		MASLD+low MM-NO	1.68 (1.25, 2.27)	0.001	1.28 (0.94, 1.75)	0.112	1.02 (0.71, 1.47)	0.904
		MASLD+low MM-O	0.59 (0.22, 1.58)	0.295	0.46 (0.17, 1.23)	0.121	0.27 (0.07, 1.06)	0.061
		MASLD+low MM	1.46 (1.09, 1.94)	0.010	1.11 (0.83, 1.49)	0.496	0.87 (0.61, 1.23)	0.424
Non-liver cancers	Younger	Healthy	Ref		Ref		Ref	
		MASLD+normal MM	1.17 (1.13, 1.22)	<0.001	1.18 (1.14, 1.23)	<0.001	1.12 (1.06, 1.18)	<0.001
		MASLD+low MM-NO	0.99 (0.41, 2.39)	0.989	1.04 (0.43, 2.51)	0.925	0.81 (0.30, 2.17)	0.673
		MASLD+low MM-O	2.39 (0.77, 7.43)	0.131	2.59 (0.84, 8.04)	0.099	2.72 (0.88, 8.46)	0.083
		MASLD+low MM	1.27 (0.64, 2.55)	0.495	1.34 (0.67, 2.69)	0.404	1.16 (0.55, 2.44)	0.695
	Older	Healthy	Ref		Ref		Ref	
		MASLD+normal MM	1.13 (1.11, 1.16)	<0.001	1.14 (1.11, 1.17)	<0.001	1.09 (1.06, 1.13)	<0.001
		MASLD+low MM-NO	1.35 (1.03, 1.77)	0.032	1.39 (1.06, 1.83)	0.018	1.14 (0.83, 1.56)	0.419
		MASLD+low MM-O	1.08 (0.56, 2.08)	0.812	1.17 (0.61, 2.24)	0.643	1.06 (0.50, 2.22)	0.887
		MASLD+low MM	1.30 (1.01, 1.67)	0.041	1.35 (1.05, 1.74)	0.019	1.13 (0.84, 1.51)	0.426
Obesity-related cancers	Younger	Healthy	Ref		Ref		Ref	

	MASLD+normal MM	1.46 (1.37, 1.57)	<0.001	1.49 (1.39, 1.60)	<0.001	1.32 (1.21, 1.45)	<0.001
	MASLD+low MM-NO	—	—	—	—	—	—
	MASLD+low MM-O	2.10 (0.30, 14.91)	0.459	2.23 (0.31, 15.83)	0.423	2.26 (0.32, 16.09)	0.414
	MASLD+low MM	0.44 (0.06, 3.15)	0.417	0.47 (0.07, 3.33)	0.448	0.41 (0.06, 2.90)	0.369
Older	Healthy	Ref		Ref		Ref	
	MASLD+normal MM	1.36 (1.30, 1.42)	<0.001	1.38 (1.31, 1.44)	<0.001	1.24 (1.17, 1.31)	<0.001
	MASLD+low MM-NO	1.56 (1.04, 2.33)	0.031	1.61 (1.08, 2.41)	0.020	1.35 (0.87, 2.11)	0.183
	MASLD+low MM-O	1.41 (0.59, 3.40)	0.441	1.53 (0.63, 3.67)	0.345	1.37 (0.51, 3.65)	0.532
	MASLD+low MM	1.53 (1.06, 2.20)	0.023	1.60 (1.11, 2.30)	0.012	1.35 (0.90, 2.03)	0.142

Table S25. Association between combined MASLD and physical performance (PP) status and clinical outcomes, stratified by age.

			Model1		Model2		Model3	
**Outcomes**		**Groups**	**HR (95% CI)**	**P value**	**HR (95% CI)**	**P value**	**HR (95% CI)**	**P value**
Death	Younger	Healthy	Ref		Ref		Ref	
		MASLD+normal PP	1.31 (1.24, 1.37)	<0.001	1.01 (0.96, 1.07)	0.593	1.04 (0.97, 1.11)	0.263
		MASLD+low PP-NO	2.90 (2.52, 3.33)	<0.001	1.09 (0.95, 1.26)	0.222	1.09 (0.93, 1.28)	0.280
		MASLD+low PP-O	2.84 (2.60, 3.10)	<0.001	1.09 (1.00, 1.20)	0.050	1.10 (0.99, 1.22)	0.085
		MASLD+low PP	2.86 (2.64, 3.09)	<0.001	1.09 (1.01, 1.19)	0.030	1.10 (1.00, 1.21)	0.060
	Older	Healthy	Ref		Ref		Ref	
		MASLD+normal PP	1.21 (1.18, 1.24)	<0.001	0.96 (0.94, 0.99)	0.004	0.99 (0.96, 1.03)	0.608
		MASLD+low PP-NO	2.49 (2.35, 2.65)	<0.001	1.33 (1.25, 1.42)	<0.001	1.32 (1.23, 1.41)	<0.001
		MASLD+low PP-O	2.38 (2.28, 2.48)	<0.001	1.18 (1.13, 1.23)	<0.001	1.21 (1.15, 1.27)	<0.001
		MASLD+low PP	2.41 (2.32, 2.50)	<0.001	1.22 (1.18, 1.27)	<0.001	1.24 (1.18, 1.30)	<0.001
Cirrhosis	Younger	Healthy	Ref		Ref		Ref	
		MASLD+normal PP	2.72 (2.27, 3.24)	<0.001	3.18 (2.62, 3.86)	<0.001	2.84 (2.22, 3.63)	<0.001
		MASLD+low PP-NO	3.77 (2.33, 6.08)	<0.001	3.98 (2.45, 6.47)	<0.001	2.21 (1.27, 3.85)	0.005
		MASLD+low PP-O	6.20 (4.76, 8.07)	<0.001	7.16 (5.44, 9.43)	<0.001	5.76 (4.13, 8.03)	<0.001
		MASLD+low PP	5.55 (4.33, 7.11)	<0.001	6.26 (4.82, 8.12)	<0.001	4.55 (3.31, 6.25)	<0.001
	Older	Healthy	Ref		Ref		Ref	
		MASLD+normal PP	3.28 (2.80, 3.83)	<0.001	3.80 (3.19, 4.51)	<0.001	3.33 (2.70, 4.10)	<0.001
		MASLD+low PP-NO	6.48 (4.87, 8.62)	<0.001	7.00 (5.19, 9.44)	<0.001	3.52 (2.45, 5.06)	<0.001
		MASLD+low PP-O	7.93 (6.51, 9.65)	<0.001	8.85 (7.16, 10.95)	<0.001	6.02 (4.67, 7.76)	<0.001
		MASLD+low PP	7.52 (6.26, 9.04)	<0.001	8.32 (6.81, 10.17)	<0.001	5.28 (4.13, 6.76)	<0.001
HCC	Younger	Healthy	Ref		Ref		Ref	
		MASLD+normal PP	1.68 (1.07, 2.66)	0.025	1.73 (1.05, 2.83)	0.030	1.74 (0.93, 3.26)	0.085
		MASLD+low PP-NO	4.71 (1.79, 12.35)	0.002	4.57 (1.71, 12.23)	0.002	3.42 (1.11, 10.48)	0.032
		MASLD+low PP-O	3.63 (1.71, 7.74)	0.001	3.80 (1.76, 8.23)	0.001	2.84 (1.12, 7.18)	0.028
		MASLD+low PP	3.95 (2.07, 7.56)	<0.001	4.04 (2.07, 7.90)	<0.001	3.03 (1.33, 6.88)	0.008
	Older	Healthy	Ref		Ref		Ref	
		MASLD+normal PP	2.85 (2.16, 3.77)	<0.001	2.93 (2.32, 3.70)	<0.001	1.76 (1.37, 2.26)	<0.001
		MASLD+low PP-NO	5.79 (3.50, 9.58)	<0.001	5.56 (3.49, 8.86)	<0.001	1.94 (1.17, 3.22)	0.010
		MASLD+low PP-O	5.63 (3.83, 8.26)	<0.001	5.30 (3.79, 7.40)	<0.001	2.28 (1.59, 3.27)	<0.001
		MASLD+low PP	5.68 (4.02, 8.02)	<0.001	5.38 (4.04, 7.17)	<0.001	2.17 (1.59, 2.95)	<0.001

CVD	Younger	Healthy	Ref		Ref		Ref	
		MASLD+normal PP	1.57 (1.50, 1.64)	<0.001	1.04 (1.00, 1.09)	0.080	0.93 (0.88, 0.98)	0.011
		MASLD+low PP-NO	2.92 (2.57, 3.31)	<0.001	1.55 (1.36, 1.76)	<0.001	1.39 (1.21, 1.60)	<0.001
		MASLD+low PP-O	3.19 (2.95, 3.45)	<0.001	1.35 (1.25, 1.47)	<0.001	1.14 (1.04, 1.26)	0.007
		MASLD+low PP	3.12 (2.91, 3.34)	<0.001	1.40 (1.30, 1.51)	<0.001	1.20 (1.10, 1.31)	<0.001
	Older	Healthy	Ref		Ref		Ref	
		MASLD+normal PP	1.33 (1.29, 1.36)	<0.001	1.01 (0.99, 1.04)	0.361	0.98 (0.95, 1.02)	0.292
		MASLD+low PP-NO	2.29 (2.14, 2.44)	<0.001	1.48 (1.38, 1.59)	<0.001	1.40 (1.30, 1.51)	<0.001
		MASLD+low PP-O	2.49 (2.39, 2.60)	<0.001	1.45 (1.39, 1.52)	<0.001	1.32 (1.26, 1.39)	<0.001
		MASLD+low PP	2.43 (2.34, 2.53)	<0.001	1.46 (1.40, 1.52)	<0.001	1.34 (1.28, 1.41)	<0.001
CKD	Younger	Healthy	Ref		Ref		Ref	
		MASLD+normal PP	1.96 (1.82, 2.11)	<0.001	1.96 (1.82, 2.11)	<0.001	1.05 (0.95, 1.17)	0.296
		MASLD+low PP-NO	3.79 (3.11, 4.63)	<0.001	3.79 (3.11, 4.63)	<0.001	1.43 (1.13, 1.82)	0.003
		MASLD+low PP-O	5.08 (4.55, 5.67)	<0.001	5.08 (4.55, 5.67)	<0.001	1.48 (1.28, 1.70)	<0.001
		MASLD+low PP	4.77 (4.31, 5.28)	<0.001	1.81 (1.62, 2.03)	<0.001	1.47 (1.28, 1.68)	<0.001
	Older	Healthy	Ref		Ref		Ref	
		MASLD+normal PP	1.76 (1.70, 1.82)	<0.001	1.26 (1.21, 1.31)	<0.001	1.17 (1.12, 1.22)	<0.001
		MASLD+low PP-NO	2.61 (2.39, 2.84)	<0.001	1.60 (1.47, 1.75)	<0.001	1.39 (1.25, 1.53)	<0.001
		MASLD+low PP-O	3.70 (3.52, 3.89)	<0.001	1.91 (1.80, 2.01)	<0.001	1.59 (1.50, 1.70)	<0.001
		MASLD+low PP	3.40 (3.24, 3.56)	<0.001	1.83 (1.74, 1.92)	<0.001	1.54 (1.45, 1.64)	<0.001
Non-liver cancers	Younger	Healthy	Ref		Ref		Ref	
		MASLD+normal PP	1.15 (1.10, 1.20)	<0.001	1.16 (1.12, 1.21)	<0.001	1.11 (1.05, 1.17)	<0.001
		MASLD+low PP-NO	1.29 (1.09, 1.53)	0.003	1.25 (1.05, 1.48)	0.012	1.13 (0.94, 1.37)	0.188
		MASLD+low PP-O	1.38 (1.25, 1.52)	<0.001	1.38 (1.25, 1.52)	<0.001	1.23 (1.09, 1.37)	0.001
		MASLD+low PP	1.36 (1.25, 1.48)	<0.001	1.34 (1.23, 1.47)	<0.001	1.20 (1.08, 1.33)	<0.001
	Older	Healthy	Ref		Ref		Ref	
		MASLD+normal PP	1.13 (1.10, 1.15)	<0.001	1.13 (1.10, 1.16)	<0.001	1.09 (1.06, 1.13)	<0.001
		MASLD+low PP-NO	1.19 (1.10, 1.29)	<0.001	1.15 (1.05, 1.25)	0.002	1.09 (0.99, 1.20)	0.071
		MASLD+low PP-O	1.20 (1.13, 1.26)	<0.001	1.19 (1.12, 1.26)	<0.001	1.11 (1.04, 1.18)	0.002
		MASLD+low PP	1.19 (1.14, 1.25)	<0.001	1.18 (1.12, 1.23)	<0.001	1.10 (1.04, 1.17)	0.001
Obesity-related cancers	Younger	Healthy	Ref		Ref		Ref	

	MASLD+normal PP	1.41 (1.31, 1.51)	<0.001	1.44 (1.34, 1.55)	<0.001	1.30 (1.18, 1.43)	<0.001
	MASLD+low PP-NO	1.44 (1.07, 1.95)	0.017	1.47 (1.09, 1.99)	0.012	1.23 (0.88, 1.71)	0.229
	MASLD+low PP-O	1.94 (1.68, 2.26)	<0.001	1.94 (1.67, 2.26)	<0.001	1.58 (1.33, 1.89)	<0.001
	MASLD+low PP	1.83 (1.60, 2.10)	<0.001	1.84 (1.60, 2.11)	<0.001	1.50 (1.28, 1.77)	<0.001
Older	Healthy	Ref		Ref		Ref	
	MASLD+normal PP	1.34 (1.28, 1.40)	<0.001	1.36 (1.29, 1.43)	<0.001	1.24 (1.16, 1.31)	<0.001
	MASLD+low PP-NO	1.39 (1.20, 1.61)	<0.001	1.34 (1.16, 1.56)	<0.001	1.17 (0.99, 1.38)	0.061
	MASLD+low PP-O	1.51 (1.38, 1.65)	<0.001	1.52 (1.39, 1.66)	<0.001	1.28 (1.15, 1.42)	<0.001
	MASLD+low PP	1.48 (1.37, 1.60)	<0.001	1.47 (1.36, 1.60)	<0.001	1.25 (1.13, 1.37)	<0.001

Table S26. Association between combined MASLD and muscle strength (MS) status and clinical outcomes, stratified by sex.

			Model1		Model2		Model3	
**Outcomes**		**Groups**	**HR (95% CI)**	**P value**	**HR (95% CI)**	**P value**	**HR (95% CI)**	**P value**
Death	Male	Healthy	Ref		Ref		Ref	
		MASLD+normal MS	1.30 (1.26, 1.34)	<0.001	1.01 (0.98, 1.04)	0.509	1.05 (1.01, 1.09)	0.008
		MASLD+low MS-NO	1.89 (1.73, 2.08)	<0.001	1.23 (1.12, 1.36)	<0.001	1.27 (1.15, 1.41)	<0.001
		MASLD+low MS-O	2.20 (2.02, 2.39)	<0.001	1.21 (1.11, 1.32)	<0.001	1.22 (1.11, 1.34)	<0.001
		MASLD+low MS	2.06 (1.93, 2.19)	<0.001	2.06 (1.93, 2.19)	<0.001	1.24 (1.15, 1.33)	<0.001
	Female	Healthy	Ref		Ref		Ref	
		MASLD+normal MS	1.49 (1.44, 1.54)	<0.001	0.98 (0.95, 1.02)	0.344	0.98 (0.94, 1.03)	0.527
		MASLD+low MS-NO	1.75 (1.50, 2.05)	<0.001	1.05 (0.90, 1.23)	0.546	0.97 (0.81, 1.16)	0.728
		MASLD+low MS-O	2.06 (1.88, 2.25)	<0.001	0.92 (0.84, 1.00)	0.058	0.91 (0.82, 1.01)	0.073
		MASLD+low MS	1.97 (1.82, 2.14)	<0.001	0.94 (0.87, 1.02)	0.166	0.92 (0.84, 1.01)	0.090
Cirrhosis	Male	Healthy	Ref		Ref		Ref	
		MASLD+normal MS	2.90 (2.52, 3.35)	<0.001	3.34 (2.85, 3.92)	<0.001	3.14 (2.58, 3.83)	<0.001
		MASLD+low MS-NO	4.39 (3.03, 6.36)	<0.001	4.93 (3.35, 7.28)	<0.001	2.41 (1.48, 3.92)	<0.001
		MASLD+low MS-O	5.51 (4.02, 7.56)	<0.001	6.29 (4.51, 8.77)	<0.001	4.74 (3.25, 6.92)	<0.001
		MASLD+low MS	4.99 (3.86, 6.46)	<0.001	5.66 (4.30, 7.44)	<0.001	3.59 (2.58, 5.01)	<0.001
	Female	Healthy	Ref		Ref		Ref	
		MASLD+normal MS	4.32 (3.60, 5.18)	<0.001	4.94 (4.06, 6.02)	<0.001	3.55 (2.76, 4.57)	<0.001
		MASLD+low MS-NO	5.43 (2.86, 10.30)	<0.001	6.27 (3.29, 11.95)	<0.001	3.88 (1.78, 8.44)	0.001
		MASLD+low MS-O	10.34 (7.60, 14.08)	<0.001	11.74 (8.49, 16.23)	<0.001	7.59 (5.18, 11.12)	<0.001
		MASLD+low MS	9.08 (6.79, 12.14)	<0.001	10.30 (7.59, 13.97)	<0.001	6.69 (4.65, 9.63)	<0.001
HCC	Male	Healthy	Ref		Ref		Ref	
		MASLD+normal MS	2.96 (2.24, 3.91)	<0.001	3.29 (2.41, 4.50)	<0.001	2.14 (1.50, 3.06)	<0.001
		MASLD+low MS-NO	6.26 (3.43, 11.41)	<0.001	6.79 (3.60, 12.83)	<0.001	2.58 (1.20, 5.53)	0.015
		MASLD+low MS-O	6.48 (3.68, 11.44)	<0.001	7.20 (3.95, 13.12)	<0.001	3.60 (1.88, 6.91)	<0.001
		MASLD+low MS	6.38 (4.07, 10.00)	<0.001	7.01 (4.32, 11.35)	<0.001	3.12 (1.78, 5.48)	<0.001
	Female	Healthy	Ref		Ref		Ref	
		MASLD+normal MS	2.30 (1.50, 3.53)	<0.001	2.03 (1.31, 3.13)	0.001	1.06 (0.68, 1.67)	0.789
		MASLD+low MS-NO	0.00 (0.00, Inf)	0.994	NA (NA, NA)	NA	0.00 (0.00, Inf)	0.990
		MASLD+low MS-O	4.14 (1.73, 9.87)	0.001	3.81 (1.59, 9.10)	0.003	2.43 (1.06, 5.60)	0.037
		MASLD+low MS	3.03 (1.27, 7.23)	0.013	2.78 (1.17, 6.65)	0.021	1.80 (0.78, 4.15)	0.165

CVD	Male	Healthy	Ref		Ref		Ref	
		MASLD+normal MS	1.43 (1.39, 1.47)	<0.001	1.05 (1.02, 1.08)	0.001	1.18 (1.14, 1.22)	<0.001
		MASLD+low MS-NO	1.63 (1.48, 1.80)	<0.001	1.21 (1.10, 1.34)	<0.001	1.26 (1.13, 1.40)	<0.001
		MASLD+low MS-O	2.13 (1.95, 2.31)	<0.001	1.25 (1.15, 1.37)	<0.001	1.45 (1.32, 1.59)	<0.001
		MASLD+low MS	1.88 (1.76, 2.01)	<0.001	1.23 (1.15, 1.32)	<0.001	1.13 (1.05, 1.22)	0.001
	Female	Healthy	Ref		Ref		Ref	
		MASLD+normal MS	1.64 (1.59, 1.70)	<0.001	1.09 (1.05, 1.13)	<0.001	1.02 (0.97, 1.07)	0.522
		MASLD+low MS-NO	2.18 (1.90, 2.52)	<0.001	1.43 (1.24, 1.65)	<0.001	1.33 (1.13, 1.56)	0.001
		MASLD+low MS-O	2.50 (2.29, 2.71)	<0.001	1.39 (1.27, 1.52)	<0.001	1.27 (1.15, 1.40)	<0.001
		MASLD+low MS	2.41 (2.24, 2.60)	<0.001	1.40 (1.29, 1.51)	<0.001	1.28 (1.18, 1.40)	<0.001
CKD	Male	Healthy	Ref		Ref		Ref	
		MASLD+normal MS	1.80 (1.73, 1.88)	<0.001	1.25 (1.19, 1.31)	<0.001	1.13 (1.07, 1.20)	<0.001
		MASLD+low MS-NO	2.25 (1.98, 2.57)	<0.001	1.54 (1.34, 1.76)	<0.001	1.33 (1.15, 1.55)	<0.001
		MASLD+low MS-O	3.27 (2.94, 3.65)	<0.001	1.70 (1.52, 1.90)	<0.001	1.33 (1.17, 1.51)	<0.001
		MASLD+low MS	2.78 (2.54, 3.03)	<0.001	1.63 (1.49, 1.79)	<0.001	1.33 (1.20, 1.48)	<0.001
	Female	Healthy	Ref		Ref		Ref	
		MASLD+normal MS	2.24 (2.15, 2.34)	<0.001	1.40 (1.34, 1.47)	<0.001	1.28 (1.21, 1.36)	<0.001
		MASLD+low MS-NO	2.34 (1.97, 2.79)	<0.001	1.40 (1.17, 1.69)	<0.001	1.33 (1.09, 1.62)	0.005
		MASLD+low MS-O	3.58 (3.26, 3.92)	<0.001	1.82 (1.65, 2.00)	<0.001	1.54 (1.38, 1.72)	<0.001
		MASLD+low MS	3.23 (2.97, 3.51)	<0.001	1.71 (1.57, 1.87)	<0.001	1.49 (1.35, 1.65)	<0.001
Non-liver cancers	Male	Healthy	Ref		Ref		Ref	
		MASLD+normal MS	1.09 (1.06, 1.12)	<0.001	1.09 (1.06, 1.12)	<0.001	1.08 (1.04, 1.12)	<0.001
		MASLD+low MS-NO	1.03 (0.92, 1.16)	0.606	1.03 (0.92, 1.16)	0.589	0.97 (0.85, 1.11)	0.661
		MASLD+low MS-O	1.17 (1.05, 1.30)	0.003	1.16 (1.04, 1.29)	0.008	1.09 (0.97, 1.22)	0.167
		MASLD+low MS	1.10 (1.02, 1.19)	0.015	1.10 (1.01, 1.19)	0.024	1.03 (0.94, 1.13)	0.503
	Female	Healthy	Ref		Ref		Ref	
		MASLD+normal MS	1.28 (1.24, 1.33)	<0.001	1.29 (1.24, 1.34)	<0.001	1.16 (1.11, 1.22)	<0.001
		MASLD+low MS-NO	0.90 (0.73, 1.13)	0.369	0.94 (0.75, 1.16)	0.557	0.78 (0.61, 0.99)	0.045
		MASLD+low MS-O	1.36 (1.21, 1.51)	<0.001	1.38 (1.23, 1.54)	<0.001	1.21 (1.06, 1.36)	0.003
		MASLD+low MS	1.23 (1.12, 1.36)	<0.001	1.26 (1.14, 1.39)	<0.001	1.09 (0.97, 1.22)	0.132
Obesity-related cancers	Male	Healthy	Ref		Ref		Ref	

	MASLD+normal MS	1.34 (1.27, 1.41)	<0.001	1.36 (1.29, 1.44)	<0.001	1.26 (1.18, 1.36)	<0.001
	MASLD+low MS-NO	1.25 (1.00, 1.55)	0.048	1.26 (1.01, 1.57)	0.045	1.10 (0.86, 1.40)	0.467
	MASLD+low MS-O	1.64 (1.36, 1.96)	<0.001	1.67 (1.38, 2.01)	<0.001	1.43 (1.16, 1.76)	0.001
	MASLD+low MS	1.45 (1.26, 1.67)	<0.001	1.47 (1.27, 1.70)	<0.001	1.27 (1.08, 1.50)	0.004
Female	Healthy	Ref		Ref		Ref	
	MASLD+normal MS	1.47 (1.39, 1.55)	<0.001	1.48 (1.40, 1.56)	<0.001	1.28 (1.19, 1.37)	<0.001
	MASLD+low MS-NO	0.78 (0.55, 1.12)	0.181	0.82 (0.57, 1.17)	0.266	0.67 (0.45, 1.00)	0.049
	MASLD+low MS-O	1.44 (1.22, 1.70)	<0.001	1.47 (1.24, 1.73)	<0.001	1.21 (1.00, 1.46)	0.047
	MASLD+low MS	1.26 (1.09, 1.47)	0.003	1.29 (1.10, 1.50)	0.001	1.07 (0.90, 1.27)	0.467

Table S27. Association between combined MASLD and muscle mass (MM) status and clinical outcomes, stratified by sex.

			Model1		Model2		Model3	
Outcomes		Groups	HR (95% CI)	P value	HR (95% CI)	P value	HR (95% CI)	P value
Death	Male	Healthy	Ref		Ref		Ref	
		MASLD+normal MM	1.34 (1.30, 1.38)	<0.001	1.02 (0.99, 1.05)	0.122	1.06 (1.02, 1.10)	0.002
		MASLD+low MM-NO	2.27 (1.34, 3.83)	0.002	1.78 (1.05, 3.00)	0.032	2.29 (1.30, 4.04)	0.004
		MASLD+low MM-O	—	—	—	—	—	—
		MASLD+low MM	2.10 (1.24, 3.55)	0.006	1.71 (1.01, 2.90)	0.044	2.17 (1.23, 3.82)	0.008
	Female	Healthy	Ref		Ref		Ref	
		MASLD+normal MM	1.53 (1.47, 1.58)	<0.001	0.98 (0.94, 1.01)	0.184	0.98 (0.93, 1.02)	0.330
		MASLD+low MM-NO	1.93 (1.53, 2.43)	<0.001	1.06 (0.84, 1.34)	0.618	1.05 (0.81, 1.36)	0.704
		MASLD+low MM-O	2.17 (1.43, 3.30)	<0.001	1.57 (1.03, 2.38)	0.036	1.36 (0.84, 2.19)	0.205
		MASLD+low MM	1.98 (1.62, 2.43)	<0.001	1.15 (0.94, 1.40)	0.187	1.11 (0.88, 1.39)	0.382
Cirrhosis	Male	Healthy	Ref		Ref		Ref	
		MASLD+normal MM	3.01 (2.61, 3.47)	<0.001	3.46 (2.95, 4.05)	<0.001	3.17 (2.60, 3.87)	<0.001
		MASLD+low MM-NO	—	—	—	—	—	—
		MASLD+low MM-O	—	—	—	—	—	—
		MASLD+low MM	—	—	—	—	—	—
	Female	Healthy	Ref		Ref		Ref	
		MASLD+normal MM	4.67 (3.91, 5.57)	<0.001	5.35 (4.41, 6.49)	<0.001	3.81 (2.98, 4.88)	<0.001
		MASLD+low MM-NO	5.87 (2.18, 15.85)	<0.001	4.92 (1.56, 15.47)	0.006	1.37 (0.33, 5.69)	0.663
		MASLD+low MM-O	5.37 (0.75, 38.40)	0.094	6.56 (0.92, 46.98)	0.061	NA (NA, NA)	NA
		MASLD+low MM	5.76 (2.36, 14.05)	<0.001	5.25 (1.94, 14.21)	0.001	1.26 (0.30, 5.19)	0.754
HCC	Male	Healthy	Ref		Ref		Ref	
		MASLD+normal MM	3.14 (2.39, 4.13)	<0.001	3.48 (2.56, 4.74)	<0.001	2.17 (1.52, 3.10)	<0.001
		MASLD+low MM-NO	18.64 (2.58, 134.51)	0.004	20.58 (2.83, 149.51)	0.003	11.74 (1.57, 87.77)	0.016
		MASLD+low MM-O	—	—	—	—	—	—
		MASLD+low MM	17.46 (2.42, 126.04)	0.005	19.34 (2.66, 140.49)	0.003	11.12 (1.49, 82.90)	0.019
	Female	Healthy	Ref		Ref		Ref	
		MASLD+normal MM	2.35 (1.54, 3.57)	<0.001	2.08 (1.36, 3.17)	0.001	1.13 (0.72, 1.77)	0.593
		MASLD+low MM-NO	4.87 (0.67, 35.53)	0.119	4.43 (0.61, 32.38)	0.142	1.70 (0.24, 12.19)	0.600
		MASLD+low MM-O	—	—	—	—	—	—

		MASLD+low MM	3.79 (0.52, 27.70)	0.189	3.50 (0.48, 25.60)	0.217	1.51 (0.21, 10.87)	0.681
CVD	Male	Healthy	Ref		Ref		Ref	
		MASLD+normal MM	1.45 (1.42, 1.49)	<0.001	1.06 (1.03, 1.09)	<0.001	1.00 (0.96, 1.03)	0.930
		MASLD+low MM-NO	1.13 (0.54, 2.36)	0.753	1.00 (0.48, 2.09)	0.996	0.92 (0.41, 2.05)	0.836
		MASLD+low MM-O	—	—	—	—	—	—
		MASLD+low MM	1.03 (0.49, 2.16)	0.934	0.87 (0.42, 1.84)	0.723	0.79 (0.36, 1.76)	0.569
	Female	Healthy	Ref		Ref		Ref	
		MASLD+normal MM	1.71 (1.65, 1.77)	<0.001	1.12 (1.08, 1.16)	<0.001	1.04 (0.99, 1.09)	0.112
		MASLD+low MM-NO	1.47 (1.13, 1.92)	0.005	1.08 (0.82, 1.42)	0.582	1.00 (0.74, 1.34)	0.981
		MASLD+low MM-O	1.63 (1.00, 2.67)	0.050	0.98 (0.56, 1.72)	0.940	0.96 (0.53, 1.73)	0.884
		MASLD+low MM	1.51 (1.19, 1.91)	0.001	1.06 (0.83, 1.36)	0.648	0.99 (0.76, 1.29)	0.931
CKD	Male	Healthy	Ref		Ref		Ref	
		MASLD+normal MM	1.86 (1.78, 1.93)	<0.001	1.27 (1.21, 1.33)	<0.001	1.14 (1.08, 1.21)	<0.001
		MASLD+low MM-NO	1.39 (0.52, 3.71)	0.510	1.32 (0.50, 3.53)	0.576	1.02 (0.33, 3.18)	0.970
		MASLD+low MM-O	—	—	—	—	—	—
		MASLD+low MM	1.33 (1.20, 1.48)	<0.001	1.33 (1.20, 1.48)	<0.001	1.33 (1.20, 1.48)	<0.001
	Female	Healthy	Ref		Ref		Ref	
		MASLD+normal MM	2.34 (2.24, 2.44)	<0.001	1.43 (1.37, 1.50)	<0.001	1.31 (1.23, 1.38)	<0.001
		MASLD+low MM-NO	1.83 (1.35, 2.49)	<0.001	1.29 (0.94, 1.77)	0.113	1.04 (0.72, 1.51)	0.818
		MASLD+low MM-O	0.76 (0.32, 1.83)	0.541	0.57 (0.24, 1.37)	0.206	0.39 (0.13, 1.21)	0.103
		MASLD+low MM	1.59 (1.20, 2.13)	0.001	1.13 (0.84, 1.52)	0.429	0.90 (0.63, 1.28)	0.562
Non-liver cancers	Male	Healthy	Ref		Ref		Ref	
		MASLD+normal MM	1.09 (1.06, 1.12)	<0.001	1.09 (1.06, 1.12)	<0.001	1.07 (1.04, 1.11)	<0.001
		MASLD+low MM-NO	0.87 (0.41, 1.82)	0.712	0.91 (0.43, 1.91)	0.799	0.54 (0.20, 1.45)	0.222
		MASLD+low MM-O	—	—	—	—	—	—
		MASLD+low MM	0.82 (0.39, 1.72)	0.600	0.85 (0.41, 1.79)	0.678	0.51 (0.19, 1.35)	0.176
	Female	Healthy	Ref		Ref		Ref	
		MASLD+normal MM	1.28 (1.23, 1.32)	<0.001	1.28 (1.24, 1.33)	<0.001	1.16 (1.10, 1.21)	<0.001
		MASLD+low MM-NO	1.47 (1.11, 1.94)	0.006	1.52 (1.15, 2.00)	0.003	1.23 (0.90, 1.70)	0.196
		MASLD+low MM-O	1.39 (0.79, 2.44)	0.260	1.51 (0.86, 2.66)	0.156	1.39 (0.75, 2.59)	0.294
		MASLD+low MM	1.45 (1.13, 1.87)	0.003	1.51 (1.18, 1.94)	0.001	1.26 (0.95, 1.68)	0.106

Obesity-related cancers	Male	Healthy	Ref		Ref		Ref	
		MASLD+normal MM	1.34 (1.28, 1.42)	<0.001	1.36 (1.29, 1.44)	<0.001	1.26 (1.18, 1.36)	<0.001
		MASLD+low MM-NO	0.55 (0.08, 3.90)	0.549	0.58 (0.08, 4.11)	0.584	0.57 (0.08, 4.02)	0.569
		MASLD+low MM-O	—	—	—	—	—	—
		MASLD+low MM	0.52 (0.07, 3.70)	0.514	0.55 (0.08, 3.90)	0.548	0.53 (0.08, 3.80)	0.531
	Female	Healthy	Ref		Ref		Ref	
		MASLD+normal MM	1.45 (1.38, 1.53)	<0.001	1.46 (1.38, 1.54)	<0.001	1.26 (1.17, 1.36)	<0.001
		MASLD+low MM-NO	1.53 (1.01, 2.30)	0.044	1.59 (1.06, 2.40)	0.027	1.31 (0.83, 2.07)	0.245
		MASLD+low MM-O	1.57 (0.70, 3.50)	0.271	1.69 (0.76, 3.78)	0.197	1.54 (0.64, 3.70)	0.338
		MASLD+low MM	1.53 (1.06, 2.21)	0.022	1.61 (1.12, 2.32)	0.011	1.35 (0.90, 2.04)	0.146

Table S28. Association between combined MASLD and physical performance (PP) status and clinical outcomes, stratified by sex.

			Model1		Model2		Model3	
**Outcomes**		**Groups**	**HR (95% CI)**	**P value**	**HR (95% CI)**	**P value**	**HR (95% CI)**	**P value**
Death	Male	Healthy	Ref		Ref		Ref	
		MASLD+normal PP	1.19 (1.15, 1.22)	<0.001	0.98 (0.95, 1.01)	0.175	1.03 (0.99, 1.07)	0.173
		MASLD+low PP-NO	2.50 (2.34, 2.67)	<0.001	1.37 (1.28, 1.46)	<0.001	1.35 (1.25, 1.46)	<0.001
		MASLD+low PP-O	2.45 (2.33, 2.58)	<0.001	1.18 (1.12, 1.24)	<0.001	1.22 (1.15, 1.30)	<0.001
		MASLD+low PP	2.47 (2.36, 2.58)	<0.001	1.24 (1.19, 1.30)	<0.001	1.27 (1.20, 1.34)	<0.001
	Female	Healthy	Ref		Ref		Ref	
		MASLD+normal PP	1.31 (1.26, 1.37)	<0.001	0.93 (0.90, 0.97)	0.001	0.94 (0.89, 0.99)	0.013
		MASLD+low PP-NO	2.59 (2.31, 2.91)	<0.001	1.19 (1.06, 1.33)	0.004	1.13 (0.99, 1.29)	0.062
		MASLD+low PP-O	2.45 (2.32, 2.59)	<0.001	1.10 (1.03, 1.16)	0.002	1.11 (1.03, 1.19)	0.005
		MASLD+low PP	2.47 (2.35, 2.61)	<0.001	1.11 (1.05, 1.17)	<0.001	1.11 (1.04, 1.19)	0.002
Cirrhosis	Male	Healthy	Ref		Ref		Ref	
		MASLD+normal PP	2.60 (2.25, 3.02)	<0.001	3.01 (2.55, 3.54)	<0.001	2.92 (2.39, 3.57)	<0.001
		MASLD+low PP-NO	4.73 (3.57, 6.27)	<0.001	5.04 (3.76, 6.77)	<0.001	2.81 (1.98, 3.99)	<0.001
		MASLD+low PP-O	6.76 (5.51, 8.29)	<0.001	7.71 (6.20, 9.59)	<0.001	6.04 (4.66, 7.83)	<0.001
		MASLD+low PP	6.04 (5.01, 7.28)	<0.001	6.73 (5.50, 8.24)	<0.001	4.73 (3.70, 6.06)	<0.001
	Female	Healthy	Ref		Ref		Ref	
		MASLD+normal PP	4.00 (3.32, 4.84)	<0.001	4.61 (3.76, 5.65)	<0.001	3.40 (2.63, 4.39)	<0.001
		MASLD+low PP-NO	7.02 (4.31, 11.44)	<0.001	7.37 (4.49, 12.09)	<0.001	4.44 (2.51, 7.84)	<0.001
		MASLD+low PP-O	7.83 (6.14, 9.99)	<0.001	8.80 (6.80, 11.39)	<0.001	5.60 (4.08, 7.67)	<0.001
		MASLD+low PP	7.71 (6.10, 9.73)	<0.001	8.56 (6.68, 10.96)	<0.001	5.40 (3.98, 7.34)	<0.001
HCC	Male	Healthy	Ref		Ref		Ref	
		MASLD+normal PP	2.74 (2.06, 3.63)	<0.001	3.06 (2.23, 4.21)	<0.001	2.07 (1.44, 2.98)	<0.001
		MASLD+low PP-NO	6.28 (3.87, 10.17)	<0.001	6.51 (3.90, 10.86)	<0.001	2.65 (1.46, 4.81)	0.001
		MASLD+low PP-O	5.92 (3.94, 8.90)	<0.001	6.38 (4.12, 9.89)	<0.001	2.92 (1.78, 4.79)	<0.001
		MASLD+low PP	6.05 (4.23, 8.66)	<0.001	6.05 (4.23, 8.66)	<0.001	2.82 (1.79, 4.44)	<0.001
	Female	Healthy	Ref		Ref		Ref	
		MASLD+normal PP	2.08 (1.32, 3.29)	0.002	1.87 (1.18, 2.97)	0.008	1.07 (0.67, 1.71)	0.775
		MASLD+low PP-NO	2.83 (0.68, 11.80)	0.153	2.51 (0.60, 10.47)	0.208	1.31 (0.32, 5.34)	0.706
		MASLD+low PP-O	3.63 (1.92, 6.87)	<0.001	3.07 (1.59, 5.92)	0.001	1.37 (0.68, 2.74)	0.377
		MASLD+low PP	3.50 (1.91, 6.42)	<0.001	2.98 (1.60, 5.54)	0.001	1.36 (0.72, 2.57)	0.348

CVD	Male	Healthy	Ref		Ref		Ref	
		MASLD+normal PP	1.34 (1.30, 1.38)	<0.001	1.02 (0.99, 1.05)	0.230	0.97 (0.93, 1.00)	0.059
		MASLD+low PP-NO	2.19 (2.04, 2.35)	<0.001	1.39 (1.30, 1.50)	<0.001	1.28 (1.18, 1.39)	<0.001
		MASLD+low PP-O	2.58 (2.45, 2.71)	<0.001	1.40 (1.33, 1.48)	<0.001	1.26 (1.18, 1.33)	<0.001
		MASLD+low PP	2.44 (2.33, 2.55)	<0.001	1.40 (1.34, 1.47)	<0.001	1.26 (1.20, 1.33)	<0.001
	Female	Healthy	Ref		Ref		Ref	
		MASLD+normal PP	1.47 (1.42, 1.53)	<0.001	1.02 (0.98, 1.06)	0.363	0.96 (0.91, 1.01)	0.113
		MASLD+low PP-NO	3.13 (2.81, 3.49)	<0.001	1.83 (1.64, 2.05)	<0.001	1.77 (1.56, 2.00)	<0.001
		MASLD+low PP-O	2.70 (2.56, 2.86)	<0.001	1.45 (1.37, 1.54)	<0.001	1.30 (1.22, 1.40)	<0.001
		MASLD+low PP	2.77 (2.63, 2.92)	<0.001	1.51 (1.43, 1.59)	<0.001	1.37 (1.28, 1.46)	<0.001
CKD	Male	Healthy	Ref		Ref		Ref	
		MASLD+normal PP	1.65 (1.58, 1.73)	<0.001	1.20 (1.15, 1.26)	<0.001	1.11 (1.04, 1.17)	0.001
		MASLD+low PP-NO	2.53 (2.29, 2.79)	<0.001	1.54 (1.39, 1.71)	<0.001	1.29 (1.15, 1.44)	<0.001
		MASLD+low PP-O	3.71 (3.47, 3.98)	<0.001	1.78 (1.66, 1.92)	<0.001	1.44 (1.32, 1.57)	<0.001
		MASLD+low PP	1.33 (1.20, 1.48)	<0.001	1.33 (1.20, 1.48)	<0.001	1.33 (1.20, 1.48)	<0.001
	Female	Healthy	Ref		Ref		Ref	
		MASLD+normal PP	1.96 (1.87, 2.05)	<0.001	1.29 (1.22, 1.35)	<0.001	1.20 (1.13, 1.27)	<0.001
		MASLD+low PP-NO	3.14 (2.74, 3.60)	<0.001	1.78 (1.55, 2.05)	<0.001	1.58 (1.35, 1.85)	<0.001
		MASLD+low PP-O	4.06 (3.81, 4.31)	<0.001	2.00 (1.87, 2.14)	<0.001	1.71 (1.58, 1.86)	<0.001
		MASLD+low PP	3.90 (3.68, 4.14)	<0.001	1.97 (1.84, 2.10)	<0.001	1.70 (1.57, 1.83)	<0.001
Non-liver cancers	Male	Healthy	Ref		Ref		Ref	
		MASLD+normal PP	1.09 (1.06, 1.12)	<0.001	1.09 (1.06, 1.12)	<0.001	1.08 (1.04, 1.11)	<0.001
		MASLD+low PP-NO	1.13 (1.04, 1.23)	0.004	1.09 (1.00, 1.19)	0.054	1.06 (0.97, 1.17)	0.206
		MASLD+low PP-O	1.13 (1.06, 1.20)	<0.001	1.12 (1.05, 1.19)	0.001	1.06 (0.98, 1.14)	0.126
		MASLD+low PP	1.13 (1.07, 1.19)	<0.001	1.11 (1.05, 1.17)	<0.001	1.06 (1.00, 1.13)	0.066
	Female	Healthy	Ref		Ref		Ref	
		MASLD+normal PP	1.25 (1.20, 1.30)	<0.001	1.26 (1.21, 1.31)	<0.001	1.14 (1.09, 1.20)	<0.001
		MASLD+low PP-NO	1.45 (1.24, 1.69)	<0.001	1.40 (1.20, 1.63)	<0.001	1.20 (1.01, 1.43)	0.038
		MASLD+low PP-O	1.41 (1.31, 1.51)	<0.001	1.40 (1.31, 1.51)	<0.001	1.23 (1.13, 1.34)	<0.001
		MASLD+low PP	1.41 (1.32, 1.51)	<0.001	1.40 (1.31, 1.50)	<0.001	1.23 (1.14, 1.33)	<0.001
Obesity-related cancers	Male	Healthy	Ref		Ref		Ref	

	MASLD+normal PP	1.33 (1.26, 1.40)	<0.001	1.35 (1.28, 1.43)	<0.001	1.27 (1.18, 1.36)	<0.001
	MASLD+low PP-NO	1.36 (1.16, 1.60)	<0.001	1.32 (1.12, 1.56)	0.001	1.19 (1.00, 1.43)	0.052
	MASLD+low PP-O	1.51 (1.34, 1.70)	<0.001	1.52 (1.35, 1.72)	<0.001	1.26 (1.10, 1.45)	0.001
	MASLD+low PP	1.46 (1.32, 1.61)	<0.001	1.45 (1.31, 1.61)	<0.001	1.24 (1.10, 1.39)	<0.001
Female	Healthy	Ref		Ref		Ref	
	MASLD+normal PP	1.40 (1.33, 1.48)	<0.001	1.42 (1.34, 1.50)	<0.001	1.24 (1.15, 1.34)	<0.001
	MASLD+low PP-NO	1.46 (1.16, 1.85)	0.001	1.44 (1.13, 1.82)	0.003	1.14 (0.87, 1.49)	0.356
	MASLD+low PP-O	1.70 (1.54, 1.88)	<0.001	1.69 (1.53, 1.87)	<0.001	1.41 (1.25, 1.59)	<0.001
	MASLD+low PP	1.66 (1.51, 1.82)	<0.001	1.65 (1.50, 1.82)	<0.001	1.37 (1.22, 1.54)	<0.001

Table S29. Association between combined MASLD and muscle strength (MS) status and clinical outcomes, stratified by diabetes.

			Model1		Model2		Model3	
**Outcomes**		**Groups**	**HR (95% CI)**	**P value**	**HR (95% CI)**	**P value**	**HR (95% CI)**	**P value**
Death	DM	Healthy	Ref		Ref		Ref	
		MASLD+normal MS	1.16 (1.10, 1.23)	<0.001	1.04 (0.98, 1.10)	0.163	1.03 (0.95, 1.11)	0.451
		MASLD+low MS-NO	1.48 (1.27, 1.72)	<0.001	1.28 (1.10, 1.48)	0.002	1.22 (1.03, 1.44)	0.023
		MASLD+low MS-O	1.57 (1.42, 1.73)	<0.001	1.21 (1.10, 1.34)	<0.001	1.12 (0.99, 1.25)	0.066
		MASLD+low MS	1.54 (1.41, 1.69)	<0.001	1.23 (1.12, 1.34)	<0.001	1.14 (1.03, 1.27)	0.014
	Non-DM	Healthy	Ref		Ref		Ref	
		MASLD+normal MS	1.24 (1.21, 1.27)	<0.001	1.06 (1.03, 1.09)	<0.001	1.03 (1.00, 1.07)	0.039
		MASLD+low MS-NO	1.78 (1.62, 1.96)	<0.001	1.12 (1.02, 1.23)	0.021	1.08 (0.97, 1.20)	0.164
		MASLD+low MS-O	1.85 (1.71, 2.01)	<0.001	1.23 (1.13, 1.34)	<0.001	1.15 (1.05, 1.26)	0.002
		MASLD+low MS	1.82 (1.71, 1.94)	<0.001	1.18 (1.11, 1.26)	<0.001	1.12 (1.04, 1.20)	0.002
Cirrhosis	DM	Healthy	Ref		Ref		Ref	
		MASLD+normal MS	2.12 (1.69, 2.66)	<0.001	2.91 (2.18, 3.89)	<0.001	2.40 (1.71, 3.36)	<0.001
		MASLD+low MS-NO	2.83 (1.74, 4.62)	<0.001	4.03 (2.40, 6.78)	<0.001	2.84 (1.56, 5.17)	0.001
		MASLD+low MS-O	3.69 (2.69, 5.05)	<0.001	4.85 (3.36, 7.01)	<0.001	4.00 (2.63, 6.08)	<0.001
		MASLD+low MS	3.46 (2.58, 4.65)	<0.001	4.64 (3.27, 6.57)	<0.001	3.69 (2.47, 5.51)	<0.001
	Non-DM	Healthy	Ref		Ref		Ref	
		MASLD+normal MS	2.48 (2.16, 2.85)	<0.001	2.73 (2.36, 3.16)	<0.001	2.88 (2.39, 3.46)	<0.001
		MASLD+low MS-NO	3.91 (2.53, 6.03)	<0.001	3.99 (2.52, 6.30)	<0.001	1.81 (1.01, 3.25)	0.046
		MASLD+low MS-O	4.06 (2.79, 5.91)	<0.001	4.46 (3.02, 6.60)	<0.001	4.90 (3.19, 7.52)	<0.001
		MASLD+low MS	3.99 (2.97, 5.36)	<0.001	4.25 (3.12, 5.80)	<0.001	3.19 (2.20, 4.63)	<0.001
HCC	DM	Healthy	Ref		Ref		Ref	
		MASLD+normal MS	2.05 (1.33, 3.17)	0.001	2.56 (1.50, 4.37)	0.001	1.45 (0.81, 2.62)	0.214
		MASLD+low MS-NO	1.88 (0.65, 5.43)	0.245	1.85 (0.53, 6.42)	0.332	1.01 (0.28, 3.67)	0.987
		MASLD+low MS-O	2.34 (1.17, 4.71)	0.017	2.96 (1.37, 6.40)	0.006	1.99 (0.89, 4.47)	0.094
		MASLD+low MS	2.21 (1.17, 4.18)	0.015	2.64 (1.28, 5.45)	0.009	1.68 (0.78, 3.63)	0.185
	Non-DM	Healthy	Ref		Ref		Ref	
		MASLD+normal MS	1.76 (1.31, 2.36)	<0.001	1.70 (1.28, 2.25)	<0.001	1.51 (1.04, 2.18)	0.030
		MASLD+low MS-NO	5.43 (2.71, 10.88)	<0.001	5.49 (2.82, 10.73)	<0.001	2.96 (1.26, 6.91)	0.012
		MASLD+low MS-O	4.74 (2.37, 9.49)	<0.001	4.32 (2.13, 8.77)	<0.001	4.20 (1.93, 9.11)	<0.001
		MASLD+low MS	5.06 (3.02, 8.49)	<0.001	4.87 (2.96, 8.01)	<0.001	3.54 (1.90, 6.60)	<0.001

CVD	DM	Healthy	Ref		Ref		Ref	
		MASLD+normal MS	1.17 (1.12, 1.24)	<0.001	1.06 (1.00, 1.12)	0.045	0.99 (0.93, 1.06)	0.794
		MASLD+low MS-NO	1.31 (1.14, 1.51)	<0.001	1.19 (1.02, 1.38)	0.024	1.07 (0.91, 1.26)	0.393
		MASLD+low MS-O	1.55 (1.41, 1.70)	<0.001	1.36 (1.23, 1.49)	<0.001	1.24 (1.11, 1.38)	<0.001
		MASLD+low MS	1.48 (1.36, 1.61)	<0.001	1.31 (1.20, 1.43)	<0.001	1.19 (1.08, 1.32)	0.001
	Non-DM	Healthy	Ref		Ref		Ref	
		MASLD+normal MS	1.33 (1.30, 1.36)	<0.001	1.07 (1.04, 1.09)	<0.001	1.01 (0.98, 1.04)	0.535
		MASLD+low MS-NO	1.63 (1.48, 1.80)	<0.001	1.32 (1.19, 1.45)	<0.001	1.23 (1.10, 1.37)	<0.001
		MASLD+low MS-O	1.84 (1.69, 2.00)	<0.001	1.29 (1.18, 1.40)	<0.001	1.18 (1.08, 1.30)	<0.001
		MASLD+low MS	1.75 (1.64, 1.87)	<0.001	1.30 (1.21, 1.39)	<0.001	1.20 (1.11, 1.29)	<0.001
CKD	DM	Healthy	Ref		Ref		Ref	
		MASLD+normal MS	1.38 (1.29, 1.47)	<0.001	1.28 (1.19, 1.37)	<0.001	1.15 (1.05, 1.25)	0.001
		MASLD+low MS-NO	1.46 (1.23, 1.73)	<0.001	1.38 (1.15, 1.64)	<0.001	1.23 (1.02, 1.50)	0.032
		MASLD+low MS-O	1.94 (1.75, 2.15)	<0.001	1.77 (1.58, 1.97)	<0.001	1.42 (1.25, 1.61)	<0.001
		MASLD+low MS	1.81 (1.64, 1.99)	<0.001	1.66 (1.50, 1.84)	<0.001	1.37 (1.22, 1.54)	<0.001
	Non-DM	Healthy	Ref		Ref		Ref	
		MASLD+normal MS	1.65 (1.59, 1.71)	<0.001	1.32 (1.27, 1.37)	<0.001	1.22 (1.17, 1.28)	<0.001
		MASLD+low MS-NO	2.08 (1.82, 2.38)	<0.001	1.58 (1.37, 1.82)	<0.001	1.41 (1.21, 1.64)	<0.001
		MASLD+low MS-O	2.52 (2.27, 2.80)	<0.001	1.70 (1.53, 1.90)	<0.001	1.43 (1.27, 1.62)	<0.001
		MASLD+low MS	2.34 (2.15, 2.55)	<0.001	1.65 (1.51, 1.81)	<0.001	1.42 (1.29, 1.57)	<0.001
Non-liver cancers	DM	Healthy	Ref		Ref		Ref	
		MASLD+normal MS	0.98 (0.92, 1.04)	0.497	0.99 (0.93, 1.06)	0.861	0.98 (0.90, 1.06)	0.644
		MASLD+low MS-NO	0.88 (0.72, 1.07)	0.206	0.89 (0.72, 1.09)	0.250	0.88 (0.70, 1.09)	0.247
		MASLD+low MS-O	0.93 (0.82, 1.06)	0.279	0.94 (0.82, 1.07)	0.354	0.89 (0.76, 1.03)	0.121
		MASLD+low MS	0.92 (0.82, 1.03)	0.133	0.92 (0.82, 1.04)	0.192	0.89 (0.77, 1.01)	0.073
	Non-DM	Healthy	Ref		Ref		Ref	
		MASLD+normal MS	1.09 (1.06, 1.11)	<0.001	1.09 (1.06, 1.11)	<0.001	1.10 (1.06, 1.13)	<0.001
		MASLD+low MS-NO	0.96 (0.86, 1.09)	0.547	0.98 (0.87, 1.10)	0.709	0.91 (0.80, 1.04)	0.169
		MASLD+low MS-O	1.16 (1.05, 1.28)	0.003	1.17 (1.06, 1.29)	0.003	1.16 (1.04, 1.29)	0.008
		MASLD+low MS	1.07 (0.99, 1.16)	0.070	1.08 (1.00, 1.17)	0.049	1.05 (0.96, 1.14)	0.302
Obesity-related cancers	DM	Healthy	Ref		Ref		Ref	
		MASLD+normal MS	1.20 (1.09, 1.33)	<0.001	1.25 (1.12, 1.40)	<0.001	1.16 (1.01, 1.33)	0.031

	MASLD+low MS-NO	0.80 (0.55, 1.14)	0.218	0.83 (0.57, 1.21)	0.336	0.73 (0.48, 1.10)	0.128
	MASLD+low MS-O	1.20 (0.99, 1.46)	0.060	1.28 (1.05, 1.57)	0.016	1.18 (0.94, 1.48)	0.150
	MASLD+low MS	1.10 (0.92, 1.31)	0.314	1.16 (0.96, 1.40)	0.114	1.06 (0.86, 1.31)	0.600
Non-DM	Healthy	Ref		Ref		Ref	
	MASLD+normal MS	1.26 (1.21, 1.31)	<0.001	1.27 (1.21, 1.32)	<0.001	1.23 (1.17, 1.30)	<0.001
	MASLD+low MS-NO	1.05 (0.85, 1.31)	0.629	1.06 (0.85, 1.32)	0.599	0.97 (0.76, 1.23)	0.801
	MASLD+low MS-O	1.25 (1.05, 1.47)	0.011	1.24 (1.04, 1.47)	0.017	1.15 (0.95, 1.39)	0.153
	MASLD+low MS	1.17 (1.02, 1.33)	0.025	1.16 (1.01, 1.33)	0.032	1.07 (0.92, 1.25)	0.360

Table S30. Association between combined MASLD and muscle mass (MM) status and clinical outcomes, stratified by diabetes.

			Model1		Model2		Model3	
Outcomes		Groups	HR (95% CI)	P value	HR (95% CI)	P value	HR (95% CI)	P value
Death	DM	Healthy	Ref		Ref		Ref	
		MASLD+normal MM	1.20 (1.13, 1.27)	<0.001	1.06 (1.00, 1.12)	0.042	1.04 (0.97, 1.12)	0.302
		MASLD+low MM-NO	1.68 (1.04, 2.71)	0.033	1.53 (0.95, 2.46)	0.084	1.77 (1.07, 2.90)	0.025
		MASLD+low MM-O	1.17 (0.44, 3.11)	0.759	1.69 (0.63, 4.51)	0.295	1.52 (0.49, 4.74)	0.466
		MASLD+low MM	1.55 (1.01, 2.39)	0.046	1.56 (1.01, 2.39)	0.045	1.72 (1.09, 2.72)	0.020
	Non-DM	Healthy	Ref		Ref		Ref	
		MASLD+normal MM	1.27 (1.24, 1.31)	<0.001	1.07 (1.04, 1.10)	<0.001	1.04 (1.01, 1.07)	0.015
		MASLD+low MM-NO	1.81 (1.43, 2.29)	<0.001	1.02 (0.81, 1.29)	0.851	1.03 (0.79, 1.33)	0.845
		MASLD+low MM-O	2.01 (1.27, 3.20)	0.003	1.44 (0.91, 2.29)	0.121	1.19 (0.70, 2.01)	0.521
		MASLD+low MM	1.85 (1.50, 2.28)	<0.001	1.09 (0.88, 1.34)	0.434	1.05 (0.83, 1.33)	0.657
Cirrhosis	DM	Healthy	Ref		Ref		Ref	
		MASLD+normal MM	2.24 (1.79, 2.80)	<0.001	3.06 (2.30, 4.08)	<0.001	2.52 (1.80, 3.52)	<0.001
		MASLD+low MM-NO	5.81 (1.83, 18.44)	0.003	5.11 (1.24, 21.07)	0.024	1.77 (0.24, 13.26)	0.577
		MASLD+low MM-O	6.60 (0.92, 47.49)	0.061	9.73 (1.34, 70.75)	0.025	—	—
		MASLD+low MM	5.99 (2.19, 16.39)	<0.001	6.07 (1.89, 19.53)	0.002	1.48 (0.20, 11.02)	0.700
	Non-DM	Healthy	Ref		Ref		Ref	
		MASLD+normal MM	2.57 (2.25, 2.94)	<0.001	2.82 (2.44, 3.26)	<0.001	2.95 (2.45, 3.54)	<0.001
		MASLD+low MM-NO	1.42 (0.20, 10.15)	0.724	1.54 (0.22, 11.02)	0.664	0.62 (0.09, 4.45)	0.631
		MASLD+low MM-O	—	—	—	—	—	—
		MASLD+low MM	1.17 (0.16, 8.31)	0.878	1.29 (0.18, 9.20)	0.799	0.58 (0.08, 4.19)	0.590
HCC	DM	Healthy	Ref		Ref		Ref	
		MASLD+normal MM	2.07 (1.35, 3.19)	0.001	2.57 (1.51, 4.39)	0.001	1.48 (0.82, 2.66)	0.189
		MASLD+low MM-NO	—	—	—	—	—	—
		MASLD+low MM-O	—	—	—	—	—	—
		MASLD+low MM	—	—	—	—	—	—
	Non-DM	Healthy	Ref		Ref		Ref	
		MASLD+normal MM	1.92 (1.44, 2.55)	<0.001	1.84 (1.39, 2.45)	<0.001	1.61 (1.12, 2.32)	0.011
		MASLD+low MM-NO	11.05 (2.70, 45.23)	0.001	10.38 (2.58, 41.81)	0.001	3.90 (0.87, 17.53)	0.076
		MASLD+low MM-O	—	—	—	—	—	—
		MASLD+low MM	9.09 (2.22, 37.26)	0.002	8.65 (2.15, 34.85)	0.002	3.65 (0.82, 16.26)	0.090

CVD	DM	Healthy	Ref		Ref		Ref	
		MASLD+normal MM	1.20 (1.14, 1.27)	<0.001	1.08 (1.02, 1.14)	0.006	1.01 (0.94, 1.08)	0.789
		MASLD+low MM-NO	1.57 (0.99, 2.51)	0.056	1.62 (1.00, 2.61)	0.050	1.62 (0.97, 2.70)	0.064
		MASLD+low MM-O	1.32 (0.55, 3.19)	0.531	1.30 (0.49, 3.46)	0.605	1.06 (0.34, 3.29)	0.924
		MASLD+low MM	1.51 (1.00, 2.28)	0.049	1.54 (1.00, 2.38)	0.049	1.49 (0.93, 2.38)	0.096
	Non-DM	Healthy	Ref		Ref		Ref	
		MASLD+normal MM	1.36 (1.32, 1.39)	<0.001	1.08 (1.05, 1.11)	<0.001	1.02 (0.99, 1.05)	0.183
		MASLD+low MM-NO	1.21 (0.89, 1.63)	0.222	0.93 (0.69, 1.26)	0.656	0.86 (0.62, 1.19)	0.361
		MASLD+low MM-O	1.34 (0.74, 2.43)	0.328	0.77 (0.39, 1.55)	0.469	0.83 (0.42, 1.66)	0.602
		MASLD+low MM	1.23 (0.94, 1.61)	0.128	0.90 (0.68, 1.19)	0.475	0.85 (0.63, 1.15)	0.295
CKD	DM	Healthy	Ref		Ref		Ref	
		MASLD+normal MM	1.43 (1.34, 1.52)	<0.001	1.32 (1.23, 1.41)	<0.001	1.17 (1.08, 1.27)	<0.001
		MASLD+low MM-NO	1.13 (0.64, 2.00)	0.673	1.27 (0.72, 2.25)	0.406	1.05 (0.54, 2.03)	0.888
		MASLD+low MM-O	0.26 (0.04, 1.88)	0.183	0.31 (0.04, 2.24)	0.248	—	—
		MASLD+low MM	0.90 (0.52, 1.56)	0.714	1.03 (0.60, 1.78)	0.911	0.80 (0.42, 1.55)	0.517
	Non-DM	Healthy	Ref		Ref		Ref	
		MASLD+normal MM	1.69 (1.63, 1.75)	<0.001	1.35 (1.30, 1.40)	<0.001	1.24 (1.18, 1.30)	<0.001
		MASLD+low MM-NO	1.62 (1.16, 2.27)	0.005	1.21 (0.85, 1.72)	0.288	0.97 (0.64, 1.46)	0.873
		MASLD+low MM-O	0.77 (0.29, 2.06)	0.604	0.61 (0.23, 1.63)	0.325	0.51 (0.16, 1.58)	0.244
		MASLD+low MM	1.45 (1.06, 2.00)	0.022	1.09 (0.78, 1.52)	0.616	0.88 (0.59, 1.29)	0.503
Non-liver cancers	DM	Healthy	Ref		Ref		Ref	
		MASLD+normal MM	0.97 (0.92, 1.03)	0.367	0.99 (0.92, 1.05)	0.694	0.97 (0.90, 1.05)	0.482
		MASLD+low MM-NO	1.20 (0.66, 2.17)	0.550	1.22 (0.67, 2.21)	0.509	1.26 (0.68, 2.37)	0.463
		MASLD+low MM-O	0.69 (0.17, 2.78)	0.605	0.76 (0.19, 3.06)	0.704	0.90 (0.22, 3.60)	0.879
		MASLD+low MM	1.08 (0.62, 1.86)	0.788	1.12 (0.65, 1.93)	0.688	1.18 (0.67, 2.10)	0.564
	Non-DM	Healthy	Ref		Ref		Ref	
		MASLD+normal MM	1.09 (1.06, 1.11)	<0.001	1.09 (1.06, 1.11)	<0.001	1.09 (1.06, 1.13)	<0.001
		MASLD+low MM-NO	1.18 (0.88, 1.58)	0.263	1.22 (0.92, 1.63)	0.173	0.96 (0.68, 1.36)	0.827
		MASLD+low MM-O	1.24 (0.67, 2.30)	0.502	1.33 (0.72, 2.48)	0.366	1.24 (0.62, 2.47)	0.551
		MASLD+low MM	1.19 (0.91, 1.55)	0.195	1.24 (0.95, 1.61)	0.107	1.01 (0.74, 1.37)	0.970

Obesity-related cancers	DM	Healthy	Ref		Ref		Ref	
		MASLD+normal MM	1.19 (1.08, 1.31)	0.001	1.24 (1.11, 1.39)	<0.001	1.15 (1.00, 1.31)	0.044
		MASLD+low MM-NO	2.13 (1.06, 4.28)	0.035	2.26 (1.12, 4.55)	0.023	2.35 (1.16, 4.77)	0.018
		MASLD+low MM-O	1.80 (0.45, 7.24)	0.406	2.08 (0.52, 8.35)	0.303	2.24 (0.55, 9.06)	0.257
		MASLD+low MM	2.05 (1.10, 3.84)	0.025	2.22 (1.18, 4.16)	0.013	2.33 (1.23, 4.40)	0.009
	Non-DM	Healthy	Ref		Ref		Ref	
		MASLD+normal MM	1.25 (1.20, 1.31)	<0.001	1.26 (1.21, 1.32)	<0.001	1.23 (1.16, 1.29)	<0.001
		MASLD+low MM-NO	1.07 (0.65, 1.74)	0.793	1.11 (0.68, 1.81)	0.687	0.85 (0.48, 1.50)	0.575
		MASLD+low MM-O	1.19 (0.45, 3.18)	0.723	1.28 (0.48, 3.40)	0.625	1.09 (0.35, 3.38)	0.884
		MASLD+low MM	1.09 (0.70, 1.69)	0.698	1.14 (0.73, 1.76)	0.568	0.89 (0.53, 1.48)	0.650

Table S31. Association between combined MASLD and physical performance (PP) status and clinical outcomes, stratified by diabetes.

			Model1		Model2		Model3	
**Outcomes**		**Groups**	**HR (95% CI)**	**P value**	**HR (95% CI)**	**P value**	**HR (95% CI)**	**P value**
Death	DM	Healthy	Ref		Ref		Ref	
		MASLD+normal PP	1.01 (0.96, 1.08)	0.635	0.96 (0.91, 1.02)	0.238	0.96 (0.89, 1.04)	0.335
		MASLD+low PP-NO	1.74 (1.56, 1.94)	<0.001	1.37 (1.22, 1.52)	<0.001	1.31 (1.15, 1.48)	<0.001
		MASLD+low PP-O	1.76 (1.64, 1.89)	<0.001	1.29 (1.20, 1.39)	<0.001	1.24 (1.14, 1.36)	<0.001
		MASLD+low PP	1.76 (1.64, 1.88)	<0.001	1.31 (1.22, 1.40)	<0.001	1.26 (1.15, 1.37)	<0.001
	Non-DM	Healthy	Ref		Ref		Ref	
		MASLD+normal PP	1.14 (1.11, 1.17)	<0.001	1.01 (0.99, 1.04)	0.308	1.00 (0.97, 1.03)	0.960
		MASLD+low PP-NO	2.52 (2.35, 2.69)	<0.001	1.27 (1.19, 1.36)	<0.001	1.18 (1.10, 1.28)	<0.001
		MASLD+low PP-O	2.11 (2.01, 2.22)	<0.001	1.40 (1.33, 1.47)	<0.001	1.29 (1.22, 1.36)	<0.001
		MASLD+low PP	2.24 (2.15, 2.34)	<0.001	1.35 (1.29, 1.41)	<0.001	1.25 (1.19, 1.32)	<0.001
Cirrhosis	DM	Healthy	Ref		Ref		Ref	
		MASLD+normal PP	1.93 (1.53, 2.43)	<0.001	2.65 (1.97, 3.55)	<0.001	2.28 (1.62, 3.20)	<0.001
		MASLD+low PP-NO	2.65 (1.79, 3.92)	<0.001	3.61 (2.35, 5.56)	<0.001	2.01 (1.21, 3.33)	0.007
		MASLD+low PP-O	3.34 (2.59, 4.31)	<0.001	4.52 (3.30, 6.18)	<0.001	3.47 (2.41, 4.99)	<0.001
		MASLD+low PP	3.21 (2.50, 4.12)	<0.001	4.35 (3.19, 5.91)	<0.001	3.18 (2.22, 4.56)	<0.001
	Non-DM	Healthy	Ref		Ref		Ref	
		MASLD+normal PP	2.36 (2.05, 2.71)	<0.001	2.60 (2.24, 3.02)	<0.001	2.77 (2.30, 3.35)	<0.001
		MASLD+low PP-NO	4.65 (3.39, 6.38)	<0.001	4.47 (3.22, 6.21)	<0.001	2.79 (1.90, 4.10)	<0.001
		MASLD+low PP-O	3.85 (2.99, 4.95)	<0.001	4.17 (3.21, 5.41)	<0.001	4.46 (3.32, 5.98)	<0.001
		MASLD+low PP	4.11 (3.32, 5.08)	<0.001	4.27 (3.42, 5.33)	<0.001	3.74 (2.88, 4.87)	<0.001
HCC	DM	Healthy	Ref		Ref		Ref	
		MASLD+normal PP	1.95 (1.25, 3.03)	0.003	2.47 (1.44, 4.26)	0.001	1.52 (0.84, 2.74)	0.169
		MASLD+low PP-NO	2.55 (1.24, 5.23)	0.011	2.92 (1.30, 6.54)	0.010	0.86 (0.32, 2.30)	0.767
		MASLD+low PP-O	2.43 (1.45, 4.08)	0.001	2.83 (1.53, 5.23)	0.001	1.41 (0.71, 2.77)	0.326
		MASLD+low PP	2.45 (1.49, 4.03)	<0.001	2.85 (1.57, 5.16)	0.001	1.28 (0.66, 2.50)	0.466
	Non-DM	Healthy	Ref		Ref		Ref	
		MASLD+normal PP	1.72 (1.28, 2.32)	<0.001	1.65 (1.24, 2.18)	<0.001	1.44 (0.98, 2.11)	0.061
		MASLD+low PP-NO	5.19 (2.91, 9.27)	<0.001	4.88 (2.84, 8.41)	<0.001	3.54 (1.85, 6.76)	<0.001
		MASLD+low PP-O	2.83 (1.59, 5.02)	<0.001	2.80 (1.63, 4.82)	<0.001	2.32 (1.22, 4.44)	0.011
		MASLD+low PP	3.65 (2.36, 5.67)	<0.001	3.55 (2.38, 5.29)	<0.001	2.81 (1.69, 4.67)	<0.001

CVD	DM	Healthy	Ref		Ref		Ref	
		MASLD+normal PP	1.08 (1.02, 1.14)	0.005	0.99 (0.93, 1.05)	0.739	0.94 (0.88, 1.01)	0.104
		MASLD+low PP-NO	1.57 (1.41, 1.75)	<0.001	1.34 (1.20, 1.50)	<0.001	1.21 (1.07, 1.37)	0.003
		MASLD+low PP-O	1.65 (1.55, 1.76)	<0.001	1.41 (1.31, 1.51)	<0.001	1.26 (1.16, 1.37)	<0.001
		MASLD+low PP	1.64 (1.54, 1.74)	<0.001	1.39 (1.30, 1.49)	<0.001	1.25 (1.16, 1.36)	<0.001
	Non-DM	Healthy	Ref		Ref		Ref	
		MASLD+normal PP	1.25 (1.22, 1.28)	<0.001	1.02 (1.00, 1.05)	0.087	0.97 (0.94, 1.01)	0.122
		MASLD+low PP-NO	2.26 (2.10, 2.42)	<0.001	1.56 (1.45, 1.68)	<0.001	1.48 (1.37, 1.60)	<0.001
		MASLD+low PP-O	2.14 (2.03, 2.25)	<0.001	1.43 (1.36, 1.51)	<0.001	1.29 (1.21, 1.37)	<0.001
		MASLD+low PP	2.18 (2.08, 2.27)	<0.001	1.47 (1.41, 1.54)	<0.001	1.35 (1.28, 1.42)	<0.001
CKD	DM	Healthy	Ref		Ref		Ref	
		MASLD+normal PP	1.24 (1.16, 1.33)	<0.001	1.17 (1.09, 1.26)	<0.001	1.09 (1.00, 1.18)	0.060
		MASLD+low PP-NO	1.68 (1.48, 1.90)	<0.001	1.54 (1.35, 1.76)	<0.001	1.28 (1.10, 1.49)	0.001
		MASLD+low PP-O	2.03 (1.88, 2.19)	<0.001	1.79 (1.64, 1.94)	<0.001	1.46 (1.33, 1.61)	<0.001
		MASLD+low PP	1.96 (1.82, 2.11)	<0.001	1.74 (1.61, 1.89)	<0.001	1.43 (1.30, 1.57)	<0.001
	Non-DM	Healthy	Ref		Ref		Ref	
		MASLD+normal PP	1.53 (1.47, 1.59)	<0.001	1.25 (1.20, 1.30)	<0.001	1.17 (1.11, 1.23)	<0.001
		MASLD+low PP-NO	2.36 (2.13, 2.62)	<0.001	1.63 (1.46, 1.81)	<0.001	1.44 (1.28, 1.62)	<0.001
		MASLD+low PP-O	2.90 (2.72, 3.10)	<0.001	1.94 (1.81, 2.07)	<0.001	1.65 (1.53, 1.79)	<0.001
		MASLD+low PP	2.74 (2.58, 2.90)	<0.001	1.85 (1.74, 1.96)	<0.001	1.59 (1.48, 1.71)	<0.001
Non-liver cancers	DM	Healthy	Ref		Ref		Ref	
		MASLD+normal PP	0.98 (0.92, 1.05)	0.610	1.00 (0.94, 1.07)	0.932	0.99 (0.91, 1.07)	0.766
		MASLD+low PP-NO	0.92 (0.80, 1.07)	0.278	0.89 (0.76, 1.04)	0.130	0.87 (0.73, 1.03)	0.106
		MASLD+low PP-O	0.95 (0.87, 1.03)	0.196	0.95 (0.87, 1.04)	0.297	0.93 (0.84, 1.03)	0.181
		MASLD+low PP	0.94 (0.87, 1.02)	0.135	0.94 (0.86, 1.02)	0.157	0.92 (0.83, 1.02)	0.097
	Non-DM	Healthy	Ref		Ref		Ref	
		MASLD+normal PP	1.08 (1.05, 1.10)	<0.001	1.08 (1.05, 1.11)	<0.001	1.09 (1.06, 1.13)	<0.001
		MASLD+low PP-NO	1.20 (1.10, 1.31)	<0.001	1.16 (1.06, 1.27)	0.001	1.15 (1.04, 1.26)	0.005
		MASLD+low PP-O	1.13 (1.06, 1.20)	<0.001	1.12 (1.05, 1.20)	<0.001	1.11 (1.03, 1.19)	0.004
		MASLD+low PP	1.15 (1.09, 1.21)	<0.001	1.13 (1.08, 1.20)	<0.001	1.12 (1.06, 1.19)	<0.001
Obesity-related cancers	DM	Healthy	Ref		Ref		Ref	
		MASLD+normal PP	1.20 (1.08, 1.33)	<0.001	1.26 (1.12, 1.41)	<0.001	1.17 (1.02, 1.34)	0.023

	MASLD+low PP-NO	1.04 (0.81, 1.33)	0.758	1.04 (0.80, 1.34)	0.773	0.91 (0.68, 1.22)	0.540
	MASLD+low PP-O	1.20 (1.05, 1.37)	0.009	1.24 (1.07, 1.43)	0.004	1.12 (0.94, 1.33)	0.192
	MASLD+low PP	1.17 (1.03, 1.33)	0.019	1.20 (1.04, 1.38)	0.011	1.08 (0.92, 1.28)	0.352
Non-DM	Healthy	Ref		Ref		Ref	
	MASLD+normal PP	1.24 (1.19, 1.29)	<0.001	1.25 (1.19, 1.31)	<0.001	1.22 (1.15, 1.29)	<0.001
	MASLD+low PP-NO	1.35 (1.15, 1.58)	<0.001	1.32 (1.13, 1.55)	0.001	1.25 (1.05, 1.48)	0.013
	MASLD+low PP-O	1.35 (1.22, 1.50)	<0.001	1.35 (1.22, 1.50)	<0.001	1.27 (1.13, 1.43)	<0.001
	MASLD+low PP	1.35 (1.24, 1.47)	<0.001	1.34 (1.23, 1.47)	<0.001	1.26 (1.14, 1.40)	<0.001

Table S32. Association between combined MASLD and muscle strength (MS) status and clinical outcomes, stratified by FIB-4.

			Model1		Model2		Model3	
**Outcomes**		**Groups**	**HR (95% CI)**	**P value**	**HR (95% CI)**	**P value**	**HR (95% CI)**	**P value**
Death	FIB-4 <1.3	Healthy	Ref		Ref		Ref	
		MASLD+normal MS	1.38 (1.33, 1.44)	<0.001	1.03 (1.00, 1.07)	0.086	1.02 (0.97, 1.06)	0.515
		MASLD+low MS-NO	1.78 (1.56, 2.03)	<0.001	1.10 (0.96, 1.25)	0.175	0.99 (0.85, 1.15)	0.864
		MASLD+low MS-O	2.01 (1.82, 2.22)	<0.001	1.02 (0.93, 1.13)	0.673	0.96 (0.86, 1.08)	0.513
		MASLD+low MS	1.93 (1.78, 2.09)	<0.001	1.05 (0.96, 1.14)	0.283	0.97 (0.89, 1.07)	0.544
	1.3≤ FIB-4≤2.67	Healthy	Ref		Ref		Ref	
		MASLD+normal MS	1.38 (1.34, 1.43)	<0.001	1.00 (0.97, 1.03)	0.973	1.02 (0.98, 1.07)	0.266
		MASLD+low MS-NO	1.95 (1.75, 2.18)	<0.001	1.36 (1.22, 1.52)	<0.001	1.39 (1.23, 1.57)	<0.001
		MASLD+low MS-O	2.28 (2.09, 2.49)	<0.001	1.19 (1.09, 1.30)	<0.001	1.18 (1.07, 1.30)	0.001
		MASLD+low MS	2.15 (2.00, 2.30)	<0.001	1.25 (1.16, 1.34)	<0.001	1.25 (1.16, 1.35)	<0.001
	FIB-4 >2.67	Healthy	Ref		Ref		Ref	
		MASLD+normal MS	1.73 (1.56, 1.91)	<0.001	1.18 (1.06, 1.31)	0.002	1.24 (1.09, 1.41)	0.001
		MASLD+low MS-NO	2.75 (2.05, 3.69)	<0.001	1.06 (0.79, 1.44)	0.687	1.15 (0.82, 1.60)	0.411
		MASLD+low MS-O	2.32 (1.81, 2.98)	<0.001	1.27 (0.99, 1.64)	0.058	1.39 (1.05, 1.83)	0.022
		MASLD+low MS	2.48 (2.03, 3.02)	<0.001	1.18 (0.96, 1.45)	0.108	1.28 (1.02, 1.61)	0.034
Cirrhosis	FIB-4 <1.3	Healthy	Ref		Ref		Ref	
		MASLD+normal MS	2.86 (2.25, 3.64)	<0.001	3.01 (2.36, 3.84)	<0.001	2.56 (1.89, 3.47)	<0.001
		MASLD+low MS-NO	4.66 (2.33, 9.31)	<0.001	4.96 (2.48, 9.93)	<0.001	2.69 (1.13, 6.39)	0.025
		MASLD+low MS-O	8.18 (5.29, 12.66)	<0.001	9.15 (5.90, 14.18)	<0.001	6.16 (3.65, 10.40)	<0.001
		MASLD+low MS	6.89 (4.65, 10.23)	<0.001	7.57 (5.09, 11.25)	<0.001	4.82 (2.97, 7.83)	<0.001
	1.3≤ FIB-4≤2.67	Healthy	Ref		Ref		Ref	
		MASLD+normal MS	5.13 (4.28, 6.14)	<0.001	5.38 (4.49, 6.45)	<0.001	3.71 (2.96, 4.65)	<0.001
		MASLD+low MS-NO	5.79 (3.40, 9.87)	<0.001	6.17 (3.62, 10.51)	<0.001	4.41 (2.51, 7.77)	<0.001
		MASLD+low MS-O	9.56 (6.77, 13.49)	<0.001	9.76 (6.92, 13.77)	<0.001	5.77 (3.89, 8.54)	<0.001
		MASLD+low MS	8.17 (6.00, 11.11)	<0.001	8.47 (6.23, 11.51)	<0.001	5.30 (3.74, 7.53)	<0.001
	FIB-4 >2.67	Healthy	Ref		Ref		Ref	
		MASLD+normal MS	6.44 (4.94, 8.39)	<0.001	6.30 (4.83, 8.21)	<0.001	5.23 (3.76, 7.27)	<0.001
		MASLD+low MS-NO	10.13 (5.85, 17.52)	<0.001	9.36 (5.41, 16.21)	<0.001	4.29 (2.16, 8.55)	<0.001
		MASLD+low MS-O	8.64 (5.48, 13.62)	<0.001	8.85 (5.62, 13.95)	<0.001	7.96 (4.78, 13.28)	<0.001
		MASLD+low MS	9.15 (6.19, 13.51)	<0.001	9.04 (6.12, 13.34)	<0.001	6.37 (4.01, 10.10)	<0.001
HCC	FIB-4 <1.3	Healthy	Ref		Ref		Ref	

		MASLD+normal MS	1.05 (0.62, 1.76)	0.861	1.10 (0.65, 1.85)	0.722	0.76 (0.39, 1.46)	0.407
		MASLD+low MS-NO	3.45 (1.03, 11.62)	0.046	3.81 (1.13, 12.86)	0.031	2.62 (0.71, 9.60)	0.147
		MASLD+low MS-O	2.93 (1.01, 8.50)	0.047	3.29 (1.13, 9.56)	0.029	2.15 (0.68, 6.83)	0.194
		MASLD+low MS	3.13 (1.34, 7.34)	0.009	3.49 (1.49, 8.21)	0.004	2.33 (0.89, 6.06)	0.084
	1.3≤ FIB-4≤2.67	Healthy	Ref		Ref		Ref	
		MASLD+normal MS	3.08 (2.17, 4.35)	<0.001	3.19 (2.25, 4.51)	<0.001	1.44 (0.95, 2.19)	0.086
		MASLD+low MS-NO	4.60 (1.81, 11.66)	0.001	4.89 (1.93, 12.39)	0.001	2.41 (0.91, 6.38)	0.076
		MASLD+low MS-O	3.81 (1.62, 8.96)	0.002	3.95 (1.67, 9.29)	0.002	1.76 (0.72, 4.31)	0.219
		MASLD+low MS	4.13 (2.12, 8.03)	<0.001	4.32 (2.22, 8.41)	<0.001	2.00 (0.98, 4.09)	0.057
	FIB-4 >2.67	Healthy	Ref		Ref		Ref	
		MASLD+normal MS	8.03 (4.62, 13.94)	<0.001	7.84 (4.51, 13.62)	<0.001	4.55 (2.45, 8.46)	<0.001
		MASLD+low MS-NO	10.12 (3.33, 30.77)	<0.001	9.41 (3.09, 28.62)	<0.001	3.46 (0.94, 12.75)	0.062
		MASLD+low MS-O	17.26 (7.61, 39.18)	<0.001	17.58 (7.75, 39.84)	<0.001	11.91 (4.93, 28.75)	<0.001
		MASLD+low MS	14.32 (6.80, 30.17)	<0.001	14.05 (6.68, 29.55)	<0.001	7.68 (3.38, 17.42)	<0.001
CVD	FIB-4 <1.3	Healthy	Ref		Ref		Ref	
		MASLD+normal MS	1.60 (1.54, 1.65)	<0.001	1.09 (1.05, 1.13)	<0.001	1.01 (0.97, 1.06)	0.604
		MASLD+low MS-NO	2.02 (1.79, 2.27)	<0.001	1.36 (1.21, 1.54)	<0.001	1.26 (1.11, 1.44)	0.001
		MASLD+low MS-O	2.47 (2.26, 2.70)	<0.001	1.36 (1.24, 1.49)	<0.001	1.26 (1.15, 1.39)	<0.001
		MASLD+low MS	2.29 (2.13, 2.47)	<0.001	1.36 (1.26, 1.47)	<0.001	1.26 (1.16, 1.37)	<0.001
	1.3≤ FIB-4≤2.67	Healthy	Ref		Ref		Ref	
		MASLD+normal MS	1.49 (1.45, 1.54)	<0.001	1.07 (1.04, 1.11)	<0.001	1.02 (0.98, 1.06)	0.400
		MASLD+low MS-NO	1.69 (1.50, 1.90)	<0.001	1.22 (1.09, 1.38)	0.001	1.13 (0.99, 1.28)	0.062
		MASLD+low MS-O	2.24 (2.05, 2.44)	<0.001	1.32 (1.21, 1.45)	<0.001	1.18 (1.07, 1.30)	0.001
		MASLD+low MS	2.01 (1.87, 2.16)	<0.001	1.29 (1.19, 1.38)	<0.001	1.16 (1.07, 1.26)	<0.001
	FIB-4 >2.67	Healthy	Ref		Ref		Ref	
		MASLD+normal MS	1.50 (1.34, 1.67)	<0.001	1.05 (0.93, 1.18)	0.421	0.99 (0.86, 1.15)	0.898
		MASLD+low MS-NO	1.82 (1.25, 2.65)	0.002	1.39 (0.95, 2.02)	0.086	1.20 (0.79, 1.83)	0.388
		MASLD+low MS-O	2.01 (1.50, 2.70)	<0.001	1.26 (0.93, 1.69)	0.133	1.17 (0.84, 1.62)	0.349
		MASLD+low MS	1.94 (1.53, 2.46)	<0.001	1.30 (1.02, 1.66)	0.033	1.18 (0.90, 1.55)	0.230
CKD	FIB-4 <1.3	Healthy	Ref		Ref		Ref	
		MASLD+normal MS	2.23 (2.12, 2.35)	<0.001	1.35 (1.28, 1.43)	<0.001	1.25 (1.17, 1.34)	<0.001
		MASLD+low MS-NO	2.91 (2.48, 3.41)	<0.001	1.75 (1.49, 2.05)	<0.001	1.59 (1.34, 1.90)	<0.001

		MASLD+low MS-O	3.90 (3.50, 4.36)	<0.001	1.81 (1.62, 2.03)	<0.001	1.51 (1.34, 1.71)	<0.001
		MASLD+low MS	3.54 (3.22, 3.90)	<0.001	1.79 (1.63, 1.98)	<0.001	1.54 (1.38, 1.71)	<0.001
	1.3≤ FIB-4≤2.67	Healthy	Ref		Ref		Ref	
		MASLD+normal MS	2.02 (1.94, 2.11)	<0.001	1.35 (1.29, 1.41)	<0.001	1.23 (1.17, 1.30)	<0.001
		MASLD+low MS-NO	1.98 (1.69, 2.32)	<0.001	1.33 (1.14, 1.57)	<0.001	1.22 (1.03, 1.45)	0.022
		MASLD+low MS-O	3.40 (3.07, 3.76)	<0.001	1.80 (1.62, 2.00)	<0.001	1.46 (1.30, 1.64)	<0.001
		MASLD+low MS	2.83 (2.59, 3.09)	<0.001	1.64 (1.50, 1.79)	<0.001	1.38 (1.24, 1.53)	<0.001
	FIB-4 >2.67	Healthy	Ref		Ref		Ref	
		MASLD+normal MS	2.15 (1.85, 2.49)	<0.001	1.39 (1.19, 1.63)	<0.001	1.19 (0.98, 1.44)	0.087
		MASLD+low MS-NO	3.45 (2.32, 5.13)	<0.001	2.33 (1.56, 3.47)	<0.001	1.84 (1.17, 2.90)	0.009
		MASLD+low MS-O	3.31 (2.39, 4.58)	<0.001	1.77 (1.26, 2.47)	0.001	1.40 (0.96, 2.03)	0.079
		MASLD+low MS	3.36 (2.58, 4.38)	<0.001	1.96 (1.49, 2.57)	<0.001	1.54 (1.13, 2.10)	0.007
Non-liver cancers	FIB-4 <1.3	Healthy	Ref		Ref		Ref	
		MASLD+normal MS	1.16 (1.13, 1.20)	<0.001	1.15 (1.11, 1.19)	<0.001	1.14 (1.09, 1.19)	<0.001
		MASLD+low MS-NO	0.98 (0.83, 1.14)	0.765	0.96 (0.82, 1.13)	0.643	0.91 (0.76, 1.08)	0.281
		MASLD+low MS-O	1.25 (1.12, 1.40)	<0.001	1.25 (1.12, 1.40)	<0.001	1.18 (1.04, 1.34)	0.008
		MASLD+low MS	1.15 (1.04, 1.26)	0.004	1.14 (1.04, 1.25)	0.006	1.08 (0.97, 1.20)	0.154
	1.3≤ FIB-4≤2.67	Healthy	Ref		Ref		Ref	
		MASLD+normal MS	1.14 (1.10, 1.17)	<0.001	1.12 (1.09, 1.16)	<0.001	1.09 (1.05, 1.14)	<0.001
		MASLD+low MS-NO	1.04 (0.90, 1.19)	0.633	1.03 (0.90, 1.19)	0.658	0.94 (0.80, 1.10)	0.438
		MASLD+low MS-O	1.18 (1.06, 1.33)	0.004	1.18 (1.05, 1.32)	0.004	1.09 (0.96, 1.24)	0.166
		MASLD+low MS	1.12 (1.02, 1.23)	0.013	1.12 (1.02, 1.23)	0.015	1.03 (0.93, 1.14)	0.581
	FIB-4 >2.67	Healthy	Ref		Ref		Ref	
		MASLD+normal MS	1.22 (1.08, 1.38)	0.001	1.21 (1.07, 1.36)	0.002	1.16 (1.00, 1.36)	0.055
		MASLD+low MS-NO	1.40 (0.91, 2.15)	0.124	1.34 (0.87, 2.05)	0.183	1.24 (0.79, 1.97)	0.352
		MASLD+low MS-O	1.47 (1.04, 2.07)	0.030	1.49 (1.05, 2.10)	0.024	1.35 (0.91, 1.98)	0.132
		MASLD+low MS	1.44 (1.09, 1.90)	0.010	1.43 (1.08, 1.88)	0.012	1.30 (0.96, 1.78)	0.094
Obesity-related cancers	FIB-4 <1.3	Healthy	Ref		Ref		Ref	
		MASLD+normal MS	1.43 (1.35, 1.52)	<0.001	1.43 (1.35, 1.51)	<0.001	1.32 (1.23, 1.42)	<0.001
		MASLD+low MS-NO	1.07 (0.81, 1.43)	0.622	1.07 (0.80, 1.42)	0.642	1.03 (0.76, 1.39)	0.870
		MASLD+low MS-O	1.43 (1.19, 1.73)	<0.001	1.44 (1.20, 1.73)	<0.001	1.26 (1.03, 1.54)	0.025
		MASLD+low MS	1.31 (1.12, 1.53)	0.001	1.31 (1.12, 1.53)	0.001	1.18 (0.99, 1.40)	0.062

1.3≤ FIB-4≤2.67	Healthy	Ref		Ref		Ref	
	MASLD+normal MS	1.38 (1.30, 1.46)	<0.001	1.37 (1.29, 1.45)	<0.001	1.24 (1.16, 1.34)	<0.001
	MASLD+low MS-NO	1.03 (0.78, 1.36)	0.820	1.03 (0.78, 1.36)	0.823	0.83 (0.61, 1.13)	0.241
	MASLD+low MS-O	1.55 (1.29, 1.86)	<0.001	1.55 (1.29, 1.86)	<0.001	1.30 (1.06, 1.60)	0.011
	MASLD+low MS	1.35 (1.15, 1.57)	<0.001	1.34 (1.15, 1.57)	<0.001	1.12 (0.94, 1.34)	0.209
FIB-4 >2.67	Healthy	Ref		Ref		Ref	
	MASLD+normal MS	1.76 (1.44, 2.14)	<0.001	1.71 (1.40, 2.09)	<0.001	1.46 (1.13, 1.87)	0.003
	MASLD+low MS-NO	2.04 (1.07, 3.86)	0.029	1.95 (1.03, 3.70)	0.041	1.32 (0.64, 2.75)	0.451
	MASLD+low MS-O	2.46 (1.53, 3.98)	<0.001	2.46 (1.52, 3.97)	<0.001	2.06 (1.21, 3.51)	0.008
	MASLD+low MS	2.30 (1.54, 3.42)	<0.001	2.25 (1.51, 3.36)	<0.001	1.74 (1.11, 2.75)	0.017

Table S33. Association between combined MASLD and muscle mass (MM) status and clinical outcomes, stratified by FIB-4.

			Model1		Model2		Model3	
Outcomes		Groups	HR (95% CI)	P value	HR (95% CI)	P value	HR (95% CI)	P value
Death	FIB-4 <1.3	Healthy	Ref		Ref		Ref	
		MASLD+normal MM	1.42 (1.37, 1.47)	<0.001	1.39 (1.34, 1.44)	<0.001	1.24 (1.18, 1.30)	<0.001
		MASLD+low MM-NO	1.83 (1.34, 2.51)	<0.001	1.76 (1.29, 2.40)	<0.001	1.59 (1.14, 2.23)	0.007
		MASLD+low MM-O	2.12 (1.23, 3.66)	0.007	2.09 (1.21, 3.61)	0.008	1.90 (1.02, 3.53)	0.043
		MASLD+low MM	1.90 (1.45, 2.49)	<0.001	1.21 (0.92, 1.59)	0.168	1.20 (0.89, 1.61)	0.230
	1.3≤ FIB-4≤2.67	Healthy	Ref		Ref		Ref	
		MASLD+normal MM	1.43 (1.39, 1.48)	<0.001	1.02 (0.99, 1.05)	0.286	1.04 (1.00, 1.08)	0.059
		MASLD+low MM-NO	2.10 (1.55, 2.86)	<0.001	1.32 (0.97, 1.79)	0.079	1.33 (0.94, 1.87)	0.109
		MASLD+low MM-O	0.97 (0.36, 2.58)	0.947	0.97 (0.36, 2.58)	0.945	1.01 (0.38, 2.71)	0.978
		MASLD+low MM	1.90 (1.42, 2.55)	<0.001	1.28 (0.95, 1.71)	0.104	1.28 (0.93, 1.78)	0.134
	FIB-4 >2.67	Healthy	Ref		Ref		Ref	
		MASLD+normal MM	1.78 (1.61, 1.96)	<0.001	1.17 (1.06, 1.30)	0.002	1.24 (1.09, 1.41)	0.001
		MASLD+low MM-NO	1.51 (0.56, 4.03)	0.415	1.10 (0.41, 2.95)	0.849	0.84 (0.31, 2.28)	0.729
		MASLD+low MM-O	19.03 (7.09, 51.10)	<0.001	12.37 (4.59, 33.31)	<0.001	11.45 (2.77, 47.27)	0.001
		MASLD+low MM	2.79 (1.39, 5.62)	0.004	2.02 (1.00, 4.09)	0.049	1.22 (0.53, 2.78)	0.639
Cirrhosis	FIB-4 <1.3	Healthy	Ref		Ref		Ref	
		MASLD+normal MM	3.09 (2.44, 3.92)	<0.001	3.27 (2.57, 4.15)	<0.001	2.69 (1.99, 3.64)	<0.001
		MASLD+low MM-NO	—	—	—	—	—	—
		MASLD+low MM-O	—	—	—	—	—	—
		MASLD+low MM	—	—	—	—	—	—
	1.3≤ FIB-4≤2.67	Healthy	Ref		Ref		Ref	
		MASLD+normal MM	5.33 (4.45, 6.37)	<0.001	5.59 (4.67, 6.69)	<0.001	3.83 (3.06, 4.79)	<0.001
		MASLD+low MM-NO	6.32 (1.56, 25.53)	0.010	6.83 (1.69, 27.59)	0.007	2.12 (0.29, 15.36)	0.457
		MASLD+low MM-O	—	—	—	—	—	—
		MASLD+low MM	5.25 (1.30, 21.19)	0.020	5.69 (1.41, 23.01)	0.015	1.85 (0.26, 13.35)	0.543
	FIB-4 >2.67	Healthy	Ref		Ref		Ref	
		MASLD+normal MM	6.67 (5.13, 8.68)	<0.001	6.55 (5.03, 8.52)	<0.001	5.34 (3.84, 7.41)	<0.001
		MASLD+low MM-NO	3.05 (0.42, 22.04)	0.268	2.52 (0.35, 18.22)	0.360	0.97 (0.13, 7.22)	0.978
		MASLD+low MM-O	23.69 (3.26, 172.28)	0.002	18.80 (2.57, 137.32)	0.004	—	—
		MASLD+low MM	5.41 (1.32, 22.13)	0.019	4.44 (1.08, 18.24)	0.039	0.96 (0.13, 7.13)	0.968

HCC	FIB-4 <1.3	Healthy	Ref		Ref		Ref	
		MASLD+normal MM	1.16 (0.70, 1.90)	0.570	1.22 (0.74, 2.01)	0.446	0.83 (0.44, 1.57)	0.570
		MASLD+low MM-NO	8.26 (1.11, 61.56)	0.039	8.98 (1.20, 66.95)	0.032	6.05 (0.77, 47.24)	0.086
		MASLD+low MM-O	—	—	—	—	—	—
		MASLD+low MM	6.41 (0.86, 47.79)	0.070	7.02 (0.94, 52.40)	0.058	5.19 (0.67, 40.30)	0.116
	1.3≤ FIB-4≤2.67	Healthy	Ref		Ref		Ref	
		MASLD+normal MM	3.12 (2.21, 4.40)	0.000	3.23 (2.29, 4.57)	<0.001	1.46 (0.96, 2.21)	0.075
		MASLD+low MM-NO	9.96 (1.37, 72.62)	0.023	10.48 (1.44, 76.43)	0.021	6.48 (0.87, 48.33)	0.069
		MASLD+low MM-O	—	—	—	—	—	—
		MASLD+low MM	8.47 (1.16, 61.81)	0.035	8.99 (1.23, 65.61)	0.030	5.68 (0.76, 42.29)	0.090
	FIB-4 >2.67	Healthy	Ref		Ref		Ref	
		MASLD+normal MM	8.54 (4.94, 14.77)	<0.001	8.37 (4.83, 14.48)	<0.001	4.80 (2.59, 8.89)	<0.001
		MASLD+low MM-NO	—	—	—	—	—	—
		MASLD+low MM-O	—	—	—	—	—	—
		MASLD+low MM	—	—	—	—	—	—
CVD	FIB-4 <1.3	Healthy	Ref		Ref		Ref	
		MASLD+normal MM	1.64 (1.59, 1.69)	<0.001	1.11 (1.07, 1.14)	<0.001	1.03 (0.98, 1.07)	0.238
		MASLD+low MM-NO	1.60 (1.14, 2.25)	0.007	1.21 (0.86, 1.70)	0.281	1.12 (0.77, 1.63)	0.542
		MASLD+low MM-O	1.23 (0.59, 2.59)	0.582	0.92 (0.44, 1.93)	0.826	0.87 (0.39, 1.95)	0.741
		MASLD+low MM	1.52 (1.11, 2.08)	0.008	1.14 (0.84, 1.56)	0.395	1.07 (0.76, 1.50)	0.699
	1.3≤ FIB-4≤2.67	Healthy	Ref		Ref		Ref	
		MASLD+normal MM	1.53 (1.48, 1.58)	<0.001	1.09 (1.06, 1.12)	<0.001	1.03 (0.99, 1.07)	0.181
		MASLD+low MM-NO	1.37 (0.93, 2.03)	0.116	1.04 (0.70, 1.54)	0.849	0.99 (0.65, 1.50)	0.946
		MASLD+low MM-O	1.36 (0.56, 3.26)	0.497	1.07 (0.45, 2.58)	0.877	1.07 (0.45, 2.58)	0.872
		MASLD+low MM	1.37 (0.96, 1.96)	0.087	1.04 (0.73, 1.50)	0.813	1.00 (0.68, 1.46)	0.996
	FIB-4 >2.67	Healthy	Ref		Ref		Ref	
		MASLD+normal MM	1.54 (1.38, 1.72)	0.000	1.08 (0.96, 1.21)	0.220	1.01 (0.87, 1.17)	0.891
		MASLD+low MM-NO	0.43 (0.06, 3.06)	0.399	0.40 (0.06, 2.87)	0.363	0.35 (0.05, 2.52)	0.295
		MASLD+low MM-O	—	—	—	—	—	—
		MASLD+low MM	0.35 (0.05, 2.47)	0.290	0.31 (0.04, 2.21)	0.243	0.27 (0.04, 1.98)	0.199
CKD	FIB-4 <1.3	Healthy	Ref		Ref		Ref	
		MASLD+normal MM	2.33 (2.22, 2.46)	<0.001	1.39 (1.32, 1.47)	<0.001	1.27 (1.19, 1.36)	<0.001
		MASLD+low MM-NO	1.80 (1.17, 2.77)	0.007	1.34 (0.87, 2.06)	0.181	1.19 (0.74, 1.92)	0.480
		MASLD+low MM-O	0.86 (0.28, 2.66)	0.791	0.55 (0.18, 1.71)	0.300	0.47 (0.12, 1.89)	0.288

		MASLD+low MM	1.58 (1.06, 2.37)	0.026	1.14 (0.76, 1.70)	0.533	1.02 (0.65, 1.61)	0.917
	1.3≤ FIB-4≤2.67	Healthy	Ref		Ref		Ref	
		MASLD+normal MM	2.09 (2.00, 2.18)	<0.001	1.37 (1.31, 1.43)	<0.001	1.25 (1.18, 1.32)	<0.001
		MASLD+low MM-NO	1.79 (1.17, 2.76)	0.008	1.31 (0.85, 2.01)	0.217	0.99 (0.59, 1.68)	0.979
		MASLD+low MM-O	0.84 (0.21, 3.36)	0.807	0.61 (0.15, 2.45)	0.489	0.29 (0.04, 2.08)	0.220
		MASLD+low MM	1.63 (1.08, 2.46)	0.019	1.19 (0.79, 1.80)	0.400	0.86 (0.51, 1.43)	0.551
	FIB-4 >2.67	Healthy	Ref		Ref		Ref	
		MASLD+normal MM	2.28 (1.97, 2.63)	<0.001	1.45 (1.24, 1.69)	<0.001	1.23 (1.01, 1.48)	0.038
		MASLD+low MM-NO	0.63 (0.09, 4.52)	0.650	0.65 (0.09, 4.62)	0.664	0.71 (0.10, 5.14)	0.731
		MASLD+low MM-O	—	—	—	—	—	—
		MASLD+low MM	0.49 (0.07, 3.48)	0.474	0.49 (0.07, 3.53)	0.482	0.59 (0.08, 4.27)	0.598
Non-liver cancers	FIB-4 <1.3	Healthy	Ref		Ref		Ref	
		MASLD+normal MM	1.16 (1.13, 1.20)	<0.001	1.15 (1.11, 1.19)	<0.001	1.14 (1.09, 1.18)	<0.001
		MASLD+low MM-NO	1.30 (0.91, 1.85)	0.143	1.28 (0.90, 1.83)	0.164	1.10 (0.73, 1.66)	0.654
		MASLD+low MM-O	1.22 (0.61, 2.44)	0.579	1.20 (0.60, 2.40)	0.603	1.50 (0.75, 3.00)	0.253
		MASLD+low MM	1.28 (0.94, 1.76)	0.120	1.27 (0.92, 1.74)	0.141	1.18 (0.83, 1.68)	0.360
	1.3≤ FIB-4≤2.67	Healthy	Ref		Ref		Ref	
		MASLD+normal MM	1.14 (1.10, 1.17)	0.000	1.12 (1.09, 1.16)	<0.001	1.09 (1.05, 1.13)	<0.001
		MASLD+low MM-NO	1.18 (0.78, 1.80)	0.430	1.16 (0.77, 1.77)	0.479	0.98 (0.61, 1.58)	0.938
		MASLD+low MM-O	0.71 (0.18, 2.83)	0.623	0.72 (0.18, 2.87)	0.639	0.35 (0.05, 2.50)	0.296
		MASLD+low MM	1.12 (0.75, 1.67)	0.578	1.11 (0.74, 1.65)	0.623	0.89 (0.56, 1.42)	0.630
	FIB-4 >2.67	Healthy	Ref		Ref		Ref	
		MASLD+normal MM	1.23 (1.09, 1.39)	0.001	1.22 (1.08, 1.37)	0.001	1.17 (1.01, 1.37)	0.042
		MASLD+low MM-NO	2.04 (0.76, 5.47)	0.157	1.94 (0.72, 5.22)	0.188	1.05 (0.32, 3.39)	0.940
		MASLD+low MM-O	5.73 (1.42, 23.06)	0.014	5.57 (1.38, 22.44)	0.016	7.92 (1.09, 57.46)	0.041
		MASLD+low MM	2.60 (1.16, 5.82)	0.021	2.48 (1.10, 5.58)	0.028	1.35 (0.49, 3.78)	0.562
Obesity-related cancers	FIB-4 <1.3	Healthy	Ref		Ref		Ref	
		MASLD+normal MM	1.43 (1.35, 1.51)	<0.001	1.42 (1.34, 1.50)	<0.001	1.32 (1.22, 1.41)	<0.001
		MASLD+low MM-NO	1.46 (0.84, 2.51)	0.178	1.44 (0.84, 2.49)	0.187	1.31 (0.72, 2.38)	0.373
		MASLD+low MM-O	1.19 (0.38, 3.68)	0.769	1.18 (0.38, 3.67)	0.773	1.44 (0.46, 4.48)	0.525
		MASLD+low MM	1.40 (0.85, 2.28)	0.184	1.39 (0.85, 2.27)	0.193	1.34 (0.79, 2.27)	0.281
	1.3≤ FIB-4≤2.67	Healthy	Ref		Ref		Ref	

	MASLD+normal MM	1.38 (1.31, 1.46)	<0.001	1.37 (1.29, 1.45)	<0.001	1.24 (1.15, 1.33)	<0.001
	MASLD+low MM-NO	0.97 (0.46, 2.03)	0.931	0.96 (0.46, 2.01)	0.905	0.86 (0.38, 1.92)	0.713
	MASLD+low MM-O	1.70 (0.43, 6.82)	0.452	1.72 (0.43, 6.89)	0.443	0.80 (0.11, 5.69)	0.824
	MASLD+low MM	1.07 (0.56, 2.06)	0.839	1.06 (0.55, 2.04)	0.860	0.85 (0.40, 1.79)	0.671
FIB-4 >2.67	Healthy	Ref		Ref		Ref	
	MASLD+normal MM	1.77 (1.46, 2.15)	<0.001	1.73 (1.42, 2.10)	<0.001	1.46 (1.14, 1.87)	0.003
	MASLD+low MM-NO	5.89 (2.17, 15.97)	<0.001	5.32 (1.95, 14.49)	0.001	2.79 (0.83, 9.32)	0.096
	MASLD+low MM-O	5.87 (0.82, 42.15)	0.079	5.86 (0.82, 42.08)	0.079	24.59 (3.29, 183.85)	0.002
	MASLD+low MM	5.88 (2.40, 14.42)	<0.001	5.42 (2.20, 13.33)	<0.001	3.66 (1.26, 10.57)	0.017

Table S34. Association between combined MASLD and physical performance (PP) status and clinical outcomes, stratified by FIB-4.

			Model1		Model2a		Model3b	
**Outcomes**		**Groups**	**HR (95% CI)**	**P value**	**HR (95% CI)**	**P value**	**HR (95% CI)**	**P value**
Death	FIB-4 <1.3	Healthy	Ref		Ref		Ref	
		MASLD+normal PP	1.24 (1.19, 1.28)	<0.001	1.01 (0.97, 1.05)	0.651	0.99 (0.95, 1.04)	0.779
		MASLD+low PP-NO	2.42 (2.21, 2.65)	<0.001	1.19 (1.08, 1.30)	<0.001	1.14 (1.03, 1.27)	0.011
		MASLD+low PP-O	2.51 (2.36, 2.66)	<0.001	1.11 (1.05, 1.19)	0.001	1.08 (1.01, 1.16)	0.032
		MASLD+low PP	2.48 (2.36, 2.62)	<0.001	1.13 (1.07, 1.20)	<0.001	1.10 (1.03, 1.17)	0.005
	1.3≤ FIB-4≤2.67	Healthy	Ref		Ref		Ref	
		MASLD+normal PP	1.25 (1.21, 1.30)	<0.001	0.97 (0.94, 1.00)	0.065	1.00 (0.96, 1.04)	0.895
		MASLD+low PP-NO	2.62 (2.42, 2.84)	<0.001	1.41 (1.30, 1.53)	<0.001	1.35 (1.24, 1.48)	<0.001
		MASLD+low PP-O	2.49 (2.36, 2.63)	<0.001	1.18 (1.12, 1.25)	<0.001	1.22 (1.14, 1.30)	<0.001
		MASLD+low PP	2.53 (2.41, 2.65)	<0.001	1.24 (1.18, 1.31)	<0.001	1.26 (1.18, 1.33)	<0.001
	FIB-4 >2.67	Healthy	Ref		Ref		Ref	
		MASLD+normal PP	1.55 (1.40, 1.72)	<0.001	1.11 (0.99, 1.24)	0.062	1.18 (1.03, 1.34)	0.015
		MASLD+low PP-NO	3.66 (2.98, 4.51)	<0.001	1.50 (1.21, 1.86)	<0.001	1.44 (1.12, 1.85)	0.005
		MASLD+low PP-O	2.78 (2.36, 3.29)	<0.001	1.45 (1.23, 1.72)	<0.001	1.61 (1.32, 1.96)	<0.001
		MASLD+low PP	3.05 (2.65, 3.51)	<0.001	1.47 (1.27, 1.70)	<0.001	1.55 (1.30, 1.85)	<0.001
Cirrhosis	FIB-4 <1.3	Healthy	Ref		Ref		Ref	
		MASLD+normal PP	2.53 (1.97, 3.24)	<0.001	2.69 (2.09, 3.46)	<0.001	2.33 (1.71, 3.18)	<0.001
		MASLD+low PP-NO	4.91 (2.89, 8.34)	<0.001	4.70 (2.76, 8.00)	<0.001	3.62 (1.99, 6.59)	<0.001
		MASLD+low PP-O	7.19 (5.21, 9.92)	<0.001	7.51 (5.43, 10.39)	<0.001	5.65 (3.80, 8.39)	<0.001
		MASLD+low PP	6.61 (4.86, 8.97)	<0.001	6.75 (4.95, 9.20)	<0.001	5.10 (3.49, 7.46)	<0.001
	1.3≤ FIB-4≤2.67	Healthy	Ref		Ref		Ref	
		MASLD+normal PP	4.50 (3.74, 5.42)	<0.001	4.78 (3.97, 5.76)	<0.001	3.54 (2.82, 4.45)	<0.001
		MASLD+low PP-NO	8.24 (5.72, 11.88)	<0.001	7.92 (5.49, 11.42)	<0.001	3.49 (2.27, 5.37)	<0.001
		MASLD+low PP-O	11.07 (8.71, 14.05)	<0.001	10.85 (8.55, 13.78)	<0.001	5.96 (4.46, 7.98)	<0.001
		MASLD+low PP	10.31 (8.22, 12.92)	<0.001	10.05 (8.02, 12.60)	<0.001	5.23 (3.96, 6.92)	<0.001
	FIB-4 >2.67	Healthy	Ref		Ref		Ref	
		MASLD+normal PP	6.07 (4.64, 7.94)	<0.001	5.97 (4.56, 7.81)	<0.001	5.17 (3.70, 7.22)	<0.001
		MASLD+low PP-NO	8.13 (5.11, 12.95)	<0.001	6.83 (4.27, 10.92)	<0.001	3.50 (1.99, 6.16)	<0.001
		MASLD+low PP-O	10.06 (7.13, 14.20)	<0.001	10.20 (7.23, 14.38)	<0.001	7.26 (4.82, 10.95)	<0.001
		MASLD+low PP	9.46 (6.86, 13.04)	<0.001	9.03 (6.55, 12.45)	<0.001	5.85 (3.96, 8.64)	<0.001
HCC	FIB-4 <1.3	Healthy	Ref		Ref		Ref	

		MASLD+normal PP	1.14 (0.68, 1.91)	0.630	1.20 (0.71, 2.02)	0.503	0.87 (0.46, 1.67)	0.679
		MASLD+low PP-NO	2.64 (0.90, 7.76)	0.077	2.74 (0.93, 8.10)	0.068	1.80 (0.55, 5.89)	0.330
		MASLD+low PP-O	1.05 (0.36, 3.03)	0.931	1.12 (0.39, 3.26)	0.833	0.51 (0.14, 1.83)	0.302
		MASLD+low PP	1.49 (0.67, 3.35)	0.331	1.58 (0.70, 3.57)	0.270	0.85 (0.33, 2.23)	0.742
	1.3≤ FIB-4≤2.67	Healthy	Ref		Ref		Ref	
		MASLD+normal PP	2.48 (1.72, 3.56)	<0.001	2.60 (1.80, 3.74)	<0.001	1.32 (0.86, 2.02)	0.207
		MASLD+low PP-NO	6.06 (3.10, 11.87)	<0.001	5.91 (3.01, 11.60)	<0.001	1.81 (0.81, 4.03)	0.145
		MASLD+low PP-O	7.60 (4.78, 12.09)	<0.001	7.45 (4.68, 11.87)	<0.001	2.63 (1.51, 4.61)	0.001
		MASLD+low PP	7.15 (4.64, 11.01)	<0.001	7.00 (4.54, 10.79)	<0.001	2.38 (1.40, 4.05)	0.001
	FIB-4 >2.67	Healthy	Ref		Ref		Ref	
		MASLD+normal PP	8.20 (4.71, 14.28)	<0.001	8.08 (4.63, 14.09)	<0.001	5.00 (2.69, 9.31)	<0.001
		MASLD+low PP-NO	11.87 (5.13, 27.47)	<0.001	10.41 (4.47, 24.25)	<0.001	4.30 (1.65, 11.19)	0.003
		MASLD+low PP-O	8.96 (4.20, 19.11)	<0.001	8.87 (4.16, 18.90)	<0.001	3.28 (1.38, 7.80)	0.007
		MASLD+low PP	9.96 (5.09, 19.51)	<0.001	9.43 (4.81, 18.49)	<0.001	3.63 (1.67, 7.91)	0.001
CVD	FIB-4 <1.3	Healthy	Ref		Ref		Ref	
		MASLD+normal PP	1.48 (1.43, 1.53)	<0.001	1.05 (1.01, 1.09)	0.008	0.98 (0.94, 1.02)	0.385
		MASLD+low PP-NO	2.51 (2.30, 2.74)	<0.001	1.50 (1.37, 1.63)	<0.001	1.41 (1.28, 1.56)	<0.001
		MASLD+low PP-O	2.76 (2.61, 2.92)	<0.001	1.41 (1.33, 1.50)	<0.001	1.26 (1.18, 1.35)	<0.001
		MASLD+low PP	2.69 (2.56, 2.83)	<0.001	1.43 (1.36, 1.51)	<0.001	1.30 (1.22, 1.38)	<0.001
	1.3≤ FIB-4≤2.67	Healthy	Ref		Ref		Ref	
		MASLD+normal PP	1.38 (1.33, 1.42)	<0.001	1.02 (0.99, 1.06)	0.152	0.98 (0.94, 1.02)	0.300
		MASLD+low PP-NO	2.42 (2.23, 2.64)	<0.001	1.53 (1.41, 1.67)	<0.001	1.41 (1.29, 1.55)	<0.001
		MASLD+low PP-O	2.62 (2.48, 2.77)	<0.001	1.47 (1.39, 1.56)	<0.001	1.31 (1.23, 1.40)	<0.001
		MASLD+low PP	2.57 (2.44, 2.69)	<0.001	1.49 (1.41, 1.56)	<0.001	1.34 (1.26, 1.42)	<0.001
	FIB-4 >2.67	Healthy	Ref		Ref		Ref	
		MASLD+normal PP	1.39 (1.24, 1.56)	<0.001	1.00 (0.89, 1.13)	0.976	0.95 (0.82, 1.11)	0.539
		MASLD+low PP-NO	2.15 (1.62, 2.86)	<0.001	1.43 (1.07, 1.91)	0.014	1.18 (0.84, 1.66)	0.349
		MASLD+low PP-O	2.40 (1.97, 2.93)	<0.001	1.49 (1.21, 1.83)	<0.001	1.43 (1.13, 1.81)	0.003
		MASLD+low PP	2.32 (1.95, 2.76)	<0.001	1.47 (1.22, 1.76)	<0.001	1.35 (1.09, 1.67)	0.006
CKD	FIB-4 <1.3	Healthy	Ref		Ref		Ref	
		MASLD+normal PP	1.99 (1.89, 2.10)	<0.001	1.28 (1.21, 1.35)	<0.001	1.20 (1.12, 1.28)	<0.001
		MASLD+low PP-NO	3.16 (2.79, 3.58)	<0.001	1.74 (1.54, 1.97)	<0.001	1.52 (1.32, 1.75)	<0.001
		MASLD+low PP-O	4.41 (4.10, 4.75)	<0.001	1.94 (1.79, 2.09)	<0.001	1.62 (1.48, 1.77)	<0.001
		MASLD+low PP	4.09 (3.82, 4.38)	<0.001	1.89 (1.76, 2.03)	<0.001	1.60 (1.47, 1.74)	<0.001

	1.3≤ FIB-4≤2.67	Healthy	Ref		Ref		Ref	
		MASLD+normal PP	1.82 (1.74, 1.91)	<0.001	1.28 (1.22, 1.34)	<0.001	1.18 (1.12, 1.25)	<0.001
		MASLD+low PP-NO	2.67 (2.38, 3.00)	<0.001	1.60 (1.43, 1.80)	<0.001	1.40 (1.23, 1.59)	<0.001
		MASLD+low PP-O	3.84 (3.59, 4.10)	<0.001	1.92 (1.79, 2.06)	<0.001	1.60 (1.48, 1.74)	<0.001
		MASLD+low PP	3.51 (3.30, 3.73)	<0.001	1.84 (1.73, 1.96)	<0.001	1.55 (1.44, 1.67)	<0.001
	FIB-4 >2.67	Healthy	Ref		Ref		Ref	
		MASLD+normal PP	1.93 (1.65, 2.25)	<0.001	1.30 (1.10, 1.53)	0.002	1.14 (0.94, 1.39)	0.191
		MASLD+low PP-NO	2.96 (2.12, 4.13)	<0.001	1.89 (1.35, 2.65)	<0.001	1.27 (0.83, 1.95)	0.264
		MASLD+low PP-O	4.22 (3.39, 5.25)	<0.001	2.24 (1.77, 2.83)	<0.001	1.77 (1.35, 2.32)	<0.001
		MASLD+low PP	3.80 (3.12, 4.64)	<0.001	2.13 (1.73, 2.64)	<0.001	1.63 (1.27, 2.10)	<0.001
Non-liver cancers	FIB-4 <1.3	Healthy	Ref		Ref		Ref	
		MASLD+normal PP	1.14 (1.10, 1.18)	<0.001	1.14 (1.10, 1.17)	<0.001	1.13 (1.08, 1.18)	<0.001
		MASLD+low PP-NO	1.22 (1.09, 1.36)	<0.001	1.16 (1.04, 1.30)	0.010	1.11 (0.98, 1.25)	0.113
		MASLD+low PP-O	1.33 (1.24, 1.42)	<0.001	1.29 (1.21, 1.39)	<0.001	1.22 (1.13, 1.32)	<0.001
		MASLD+low PP	1.30 (1.22, 1.38)	<0.001	1.26 (1.18, 1.34)	<0.001	1.19 (1.11, 1.28)	<0.001
	1.3≤ FIB-4≤2.67	Healthy	Ref		Ref		Ref	
		MASLD+normal PP	1.13 (1.10, 1.17)	<0.001	1.12 (1.09, 1.16)	<0.001	1.09 (1.05, 1.14)	<0.001
		MASLD+low PP-NO	1.17 (1.05, 1.31)	0.004	1.13 (1.01, 1.26)	0.033	1.10 (0.98, 1.23)	0.123
		MASLD+low PP-O	1.16 (1.08, 1.25)	<0.001	1.14 (1.06, 1.23)	<0.001	1.07 (0.99, 1.17)	0.095
		MASLD+low PP	1.17 (1.10, 1.24)	<0.001	1.14 (1.07, 1.21)	<0.001	1.08 (1.01, 1.16)	0.036
	FIB-4 >2.67	Healthy	Ref		Ref		Ref	
		MASLD+normal PP	1.22 (1.08, 1.39)	0.001	1.21 (1.07, 1.38)	0.002	1.18 (1.01, 1.38)	0.039
		MASLD+low PP-NO	1.66 (1.22, 2.27)	0.001	1.53 (1.11, 2.09)	0.008	1.35 (0.94, 1.93)	0.107
		MASLD+low PP-O	1.17 (0.91, 1.51)	0.223	1.16 (0.90, 1.50)	0.252	1.05 (0.78, 1.41)	0.735
		MASLD+low PP	1.32 (1.08, 1.63)	0.008	1.28 (1.04, 1.58)	0.021	1.15 (0.89, 1.47)	0.279
Obesity-related cancers	FIB-4 <1.3	Healthy	Ref		Ref		Ref	
		MASLD+normal PP	1.39 (1.31, 1.47)	<0.001	1.39 (1.31, 1.47)	<0.001	1.30 (1.20, 1.40)	<0.001
		MASLD+low PP-NO	1.37 (1.12, 1.67)	0.002	1.33 (1.09, 1.63)	0.005	1.20 (0.96, 1.50)	0.101
		MASLD+low PP-O	1.73 (1.55, 1.93)	<0.001	1.71 (1.53, 1.91)	<0.001	1.49 (1.32, 1.69)	<0.001
		MASLD+low PP	1.64 (1.49, 1.81)	<0.001	1.62 (1.47, 1.79)	<0.001	1.42 (1.27, 1.60)	<0.001
	1.3≤ FIB-4≤2.67	Healthy	Ref		Ref		Ref	
		MASLD+normal PP	1.36 (1.28, 1.44)	<0.001	1.35 (1.27, 1.43)	<0.001	1.24 (1.15, 1.33)	<0.001
		MASLD+low PP-NO	1.37 (1.13, 1.67)	0.002	1.32 (1.09, 1.61)	0.005	1.17 (0.94, 1.45)	0.153

	MASLD+low PP-O	1.53 (1.36, 1.72)	<0.001	1.50 (1.33, 1.69)	<0.001	1.24 (1.08, 1.43)	0.002
	MASLD+low PP	1.49 (1.34, 1.65)	<0.001	1.45 (1.31, 1.62)	<0.001	1.22 (1.08, 1.39)	0.002
FIB-4 >2.67	Healthy	Ref		Ref		Ref	
	MASLD+normal PP	1.78 (1.45, 2.17)	<0.001	1.74 (1.42, 2.13)	<0.001	1.48 (1.15, 1.91)	0.002
	MASLD+low PP-NO	2.38 (1.49, 3.80)	<0.001	2.17 (1.36, 3.47)	0.001	1.62 (0.95, 2.77)	0.078
	MASLD+low PP-O	1.71 (1.16, 2.52)	0.006	1.67 (1.14, 2.46)	0.009	1.36 (0.88, 2.10)	0.167
	MASLD+low PP	1.92 (1.40, 2.64)	<0.001	1.84 (1.33, 2.52)	<0.001	1.45 (0.99, 2.10)	0.053

Table S35. Sensitivity analysis of the association between combined MASLD and muscle strength (MS) status and clinical outcomes, restricted to White participants.

		Model1		Model2		Model3	
**Outcomes**	**Groups**	**HR (95% CI)**	**P value**	**HR (95% CI)**	**P value**	**HR (95% CI)**	**P value**
Death	Healthy	Ref		Ref		Ref	
	MASLD+normal MS	1.36 (1.33, 1.39)	<0.001	1.00 (0.98, 1.03)	0.813	1.02 (0.99, 1.05)	0.126
	MASLD+low MS-NO	1.90 (1.75, 2.07)	<0.001	1.15 (1.06, 1.25)	0.001	1.17 (1.07, 1.29)	0.001
	MASLD+low MS-O	2.11 (1.98, 2.25)	<0.001	1.08 (1.01, 1.15)	0.018	1.06 (0.99, 1.14)	0.110
	MASLD+low MS	2.03 (1.93, 2.14)	<0.001	1.11 (1.05, 1.17)	<0.001	1.10 (1.03, 1.17)	0.002
Cirrhosis	Healthy	Ref		Ref		Ref	
	MASLD+normal MS	3.51 (3.12, 3.95)	<0.001	2.58 (2.25, 2.95)	<0.001	2.51 (2.13, 2.95)	<0.001
	MASLD+low MS-NO	5.32 (3.82, 7.40)	<0.001	3.78 (2.68, 5.35)	<0.001	2.48 (1.64, 3.75)	<0.001
	MASLD+low MS-O	7.66 (6.10, 9.62)	<0.001	4.00 (3.12, 5.12)	<0.001	4.10 (3.11, 5.40)	<0.001
	MASLD+low MS	6.78 (5.55, 8.28)	<0.001	7.66 (6.20, 9.46)	<0.001	5.16 (4.01, 6.63)	<0.001
HCC	Healthy	Ref		Ref		Ref	
	MASLD+normal MS	2.73 (2.15, 3.45)	<0.001	2.81 (2.18, 3.61)	<0.001	1.78 (1.32, 2.41)	<0.001
	MASLD+low MS-NO	5.50 (3.07, 9.84)	<0.001	5.48 (2.98, 10.08)	<0.001	2.53 (1.26, 5.11)	0.009
	MASLD+low MS-O	5.89 (3.62, 9.56)	<0.001	6.03 (3.65, 9.97)	<0.001	3.23 (1.86, 5.59)	<0.001
	MASLD+low MS	5.73 (3.84, 8.53)	<0.001	5.81 (3.83, 8.82)	<0.001	2.95 (1.83, 4.78)	<0.001
CVD	Healthy	Ref		Ref		Ref	
	MASLD+normal MS	1.51 (1.47, 1.54)	<0.001	1.07 (1.05, 1.10)	<0.001	1.01 (0.98, 1.04)	0.670
	MASLD+low MS-NO	1.79 (1.64, 1.95)	<0.001	1.27 (1.17, 1.39)	<0.001	1.17 (1.07, 1.29)	0.001
	MASLD+low MS-O	2.29 (2.15, 2.43)	<0.001	1.31 (1.23, 1.40)	<0.001	1.20 (1.12, 1.29)	<0.001
	MASLD+low MS	2.09 (1.98, 2.20)	<0.001	1.30 (1.23, 1.37)	<0.001	1.19 (1.12, 1.26)	<0.001
CKD	Healthy	Ref		Ref		Ref	
	MASLD+normal MS	2.00 (1.94, 2.07)	<0.001	1.32 (1.27, 1.36)	<0.001	1.20 (1.15, 1.25)	<0.001
	MASLD+low MS-NO	2.34 (2.09, 2.62)	<0.001	1.51 (1.35, 1.70)	<0.001	1.37 (1.21, 1.55)	<0.001
	MASLD+low MS-O	3.48 (3.24, 3.75)	<0.001	1.77 (1.63, 1.91)	<0.001	1.45 (1.33, 1.59)	<0.001
	MASLD+low MS	3.05 (2.86, 3.25)	<0.001	1.68 (1.57, 1.80)	<0.001	1.43 (1.33, 1.54)	<0.001
Non-liver cancers	Healthy	Ref		Ref		Ref	
	MASLD+normal MS	1.15 (1.12, 1.17)	<0.001	1.15 (1.12, 1.17)	<0.001	1.11 (1.07, 1.14)	<0.001

	MASLD+low MS-NO	1.03 (0.93, 1.15)	0.555	1.05 (0.94, 1.16)	0.421	0.95 (0.85, 1.07)	0.417
	MASLD+low MS-O	1.26 (1.16, 1.36)	<0.001	1.25 (1.16, 1.36)	<0.001	1.15 (1.05, 1.25)	0.002
	MASLD+low MS	1.17 (1.10, 1.24)	<0.001	1.17 (1.10, 1.25)	<0.001	1.07 (1.00, 1.15)	0.066
Obesity-related cancers	Healthy	Ref		Ref		Ref	
	MASLD+normal MS	1.39 (1.34, 1.44)	<0.001	1.41 (1.35, 1.47)	<0.001	1.27 (1.21, 1.34)	<0.001
	MASLD+low MS-NO	1.13 (0.94, 1.37)	0.205	1.14 (0.94, 1.39)	0.175	0.98 (0.79, 1.21)	0.822
	MASLD+low MS-O	1.56 (1.38, 1.77)	<0.001	1.58 (1.39, 1.80)	<0.001	1.33 (1.15, 1.53)	<0.001
	MASLD+low MS	1.40 (1.26, 1.56)	<0.001	1.42 (1.27, 1.58)	<0.001	1.20 (1.06, 1.36)	0.003

Table S36. Sensitivity analysis of the association between combined MASLD and muscle mass (MM) status and clinical outcomes, restricted to White participants.

		Model1		Model2		Model3	
**Outcomes**	**Groups**	**HR (95% CI)**	**P value**	**HR (95% CI)**	**P value**	**HR (95% CI)**	**P value**
Death	Healthy	Ref		Ref		Ref	
	MASLD+normal MM	1.40 (1.37, 1.43)	<0.001	1.41 (1.38, 1.44)	<0.001	1.24 (1.20, 1.28)	<0.001
	MASLD+low MM-NO	1.87 (1.50, 2.33)	<0.001	1.83 (1.47, 2.28)	<0.001	1.33 (1.04, 1.70)	0.022
	MASLD+low MM-O	1.98 (1.29, 3.04)	0.002	2.17 (1.41, 3.33)	<0.001	1.77 (1.08, 2.89)	0.023
	MASLD+low MM	1.89 (1.56, 2.30)	<0.001	1.22 (1.01, 1.48)	0.044	1.22 (0.99, 1.52)	0.066
Cirrhosis	Healthy	Ref		Ref		Ref	
	MASLD+normal MM	3.71 (3.30, 4.16)	<0.001	4.27 (3.75, 4.85)	<0.001	3.67 (3.13, 4.30)	<0.001
	MASLD+low MM-NO	4.42 (1.65, 11.84)	0.003	3.73 (1.20, 11.65)	0.023	1.43 (0.35, 5.77)	0.620
	MASLD+low MM-O	4.62 (0.65, 32.89)	0.127	5.57 (0.78, 39.73)	0.087	—	—
	MASLD+low MM	4.46 (1.84, 10.78)	0.001	4.07 (1.52, 10.93)	0.005	1.30 (0.32, 5.24)	0.717
HCC	Healthy	Ref		Ref		Ref	
	MASLD+normal MM	2.89 (2.29, 3.64)	<0.001	2.97 (2.32, 3.81)	<0.001	1.83 (1.36, 2.47)	<0.001
	MASLD+low MM-NO	9.07 (2.23, 36.92)	0.002	9.01 (2.21, 36.80)	0.002	3.98 (0.94, 16.82)	0.061
	MASLD+low MM-O	—	—	—	—	—	—
	MASLD+low MM	7.40 (1.82, 30.17)	0.005	7.41 (1.81, 30.27)	0.005	3.65 (0.87, 15.37)	0.078
CVD	Healthy	Ref		Ref		Ref	
	MASLD+normal MM	1.54 (1.51, 1.58)	<0.001	1.09 (1.06, 1.11)	<0.001	1.02 (0.99, 1.05)	0.224
	MASLD+low MM-NO	1.42 (1.10, 1.85)	0.008	1.10 (0.84, 1.43)	0.486	1.01 (0.76, 1.35)	0.922
	MASLD+low MM-O	1.22 (0.69, 2.15)	0.486	0.75 (0.39, 1.44)	0.388	0.71 (0.35, 1.41)	0.325
	MASLD+low MM	1.38 (1.09, 1.75)	0.007	1.03 (0.81, 1.32)	0.803	0.95 (0.73, 1.24)	0.730
CKD	Healthy	Ref		Ref		Ref	
	MASLD+normal MM	2.08 (2.02, 2.15)	<0.001	1.34 (1.30, 1.39)	<0.001	1.22 (1.17, 1.27)	<0.001
	MASLD+low MM-NO	1.74 (1.29, 2.35)	0.000	1.25 (0.92, 1.71)	0.153	1.05 (0.73, 1.50)	0.808
	MASLD+low MM-O	0.61 (0.23, 1.64)	0.330	0.47 (0.18, 1.24)	0.128	0.27 (0.07, 1.07)	0.062
	MASLD+low MM	1.51 (1.13, 2.01)	0.005	1.09 (0.81, 1.46)	0.579	0.88 (0.62, 1.25)	0.491
Non-liver cancers	Healthy	Ref		Ref		Ref	
	MASLD+normal MM	1.15 (1.12, 1.17)	<0.001	1.15 (1.12, 1.17)	<0.001	1.10 (1.07, 1.14)	<0.001

	MASLD+low MM-NO	1.31 (1.01, 1.71)	0.042	1.36 (1.04, 1.76)	0.023	1.10 (0.81, 1.49)	0.540
	MASLD+low MM-O	1.28 (0.73, 2.25)	0.395	1.36 (0.77, 2.40)	0.285	1.32 (0.71, 2.45)	0.382
	MASLD+low MM	1.31 (1.03, 1.66)	0.027	1.36 (1.07, 1.72)	0.012	1.14 (0.86, 1.49)	0.360
Obesity-related cancers	Healthy	Ref		Ref		Ref	
	MASLD+normal MM	1.39 (1.34, 1.44)	<0.001	1.41 (1.35, 1.47)	<0.001	1.27 (1.21, 1.34)	<0.001
	MASLD+low MM-NO	1.40 (0.93, 2.10)	0.111	1.45 (0.96, 2.18)	0.077	1.20 (0.76, 1.89)	0.428
	MASLD+low MM-O	1.56 (0.70, 3.47)	0.277	1.66 (0.75, 3.70)	0.215	1.56 (0.65, 3.75)	0.321
	MASLD+low MM	1.43 (0.99, 2.06)	0.056	1.35 (0.94, 1.95)	0.107	1.17 (0.78, 1.76)	0.434

Table S37. Sensitivity analysis of the association between combined MASLD and physical performance (PP) status and clinical outcomes, restricted to White participants.

		Model1		Model2		Model3	
**Outcomes**	**Groups**	**HR (95% CI)**	**P value**	**HR (95% CI)**	**P value**	**HR (95% CI)**	**P value**
Death	Healthy	Ref		Ref		Ref	
	MASLD+normal PP	1.22 (1.19, 1.25)	<0.001	1.24 (1.21, 1.27)	<0.001	1.13 (1.09, 1.16)	<0.001
	MASLD+low PP-NO	2.60 (2.45, 2.75)	<0.001	2.38 (2.24, 2.53)	<0.001	1.89 (1.77, 2.02)	<0.001
	MASLD+low PP-O	2.46 (2.36, 2.55)	<0.001	2.43 (2.34, 2.53)	<0.001	1.89 (1.81, 1.98)	<0.001
	MASLD+low PP	2.49 (2.41, 2.58)	<0.001	1.19 (1.15, 1.23)	<0.001	1.20 (1.15, 1.26)	<0.001
Cirrhosis	Healthy	Ref		Ref		Ref	
	MASLD+normal PP	3.15 (2.79, 3.55)	<0.001	3.64 (3.19, 4.16)	<0.001	3.31 (2.81, 3.89)	<0.001
	MASLD+low PP-NO	5.68 (4.41, 7.32)	<0.001	5.97 (4.59, 7.76)	<0.001	3.04 (2.22, 4.15)	<0.001
	MASLD+low PP-O	7.64 (6.50, 8.97)	<0.001	8.65 (7.29, 10.27)	<0.001	6.35 (5.18, 7.80)	<0.001
	MASLD+low PP	7.11 (6.11, 8.27)	<0.001	7.90 (6.72, 9.28)	<0.001	5.32 (4.36, 6.49)	<0.001
HCC	Healthy	Ref		Ref		Ref	
	MASLD+normal PP	2.50 (1.96, 3.19)	<0.001	2.60 (2.01, 3.36)	<0.001	1.73 (1.27, 2.35)	<0.001
	MASLD+low PP-NO	5.77 (3.66, 9.09)	<0.001	5.66 (3.53, 9.08)	<0.001	2.22 (1.27, 3.89)	0.005
	MASLD+low PP-O	5.30 (3.74, 7.49)	<0.001	5.22 (3.63, 7.53)	<0.001	2.45 (1.60, 3.75)	<0.001
	MASLD+low PP	5.44 (3.99, 7.41)	<0.001	5.36 (3.86, 7.43)	<0.001	2.38 (1.61, 3.52)	<0.001
CVD	Healthy	Ref		Ref		Ref	
	MASLD+normal PP	1.39 (1.36, 1.42)	<0.001	1.03 (1.00, 1.05)	0.038	0.97 (0.94, 1.00)	0.060
	MASLD+low PP-NO	2.45 (2.30, 2.60)	<0.001	1.51 (1.41, 1.60)	<0.001	1.40 (1.31, 1.50)	<0.001
	MASLD+low PP-O	2.64 (2.54, 2.75)	<0.001	1.43 (1.38, 1.49)	<0.001	1.28 (1.22, 1.34)	<0.001
	MASLD+low PP	2.59 (2.50, 2.68)	<0.001	1.45 (1.40, 1.51)	<0.001	1.31 (1.26, 1.37)	<0.001
CKD	Healthy	Ref		Ref		Ref	
	MASLD+normal PP	1.80 (1.74, 1.86)	<0.001	1.24 (1.20, 1.29)	<0.001	1.15 (1.10, 1.20)	<0.001
	MASLD+low PP-NO	2.77 (2.55, 3.01)	<0.001	1.62 (1.49, 1.77)	<0.001	1.40 (1.27, 1.54)	<0.001
	MASLD+low PP-O	3.94 (3.76, 4.14)	<0.001	1.90 (1.80, 2.00)	<0.001	1.58 (1.48, 1.67)	<0.001
	MASLD+low PP	3.63 (3.47, 3.79)	<0.001	1.83 (1.75, 1.92)	<0.001	1.53 (1.45, 1.62)	<0.001
Non-liver cancers	Healthy	Ref		Ref		Ref	

	MASLD+normal PP	1.13 (1.11, 1.16)	<0.001	1.14 (1.11, 1.17)	<0.001	1.10 (1.07, 1.13)	<0.001
	MASLD+low PP-NO	1.21 (1.12, 1.30)	<0.001	1.16 (1.07, 1.25)	<0.001	1.09 (1.00, 1.19)	0.049
	MASLD+low PP-O	1.25 (1.19, 1.31)	<0.001	1.24 (1.18, 1.30)	<0.001	1.14 (1.08, 1.21)	<0.001
	MASLD+low PP	1.23 (1.18, 1.29)	<0.001	0.98 (0.94, 1.03)	0.420	0.97 (0.92, 1.02)	0.183
Obesity-related cancers	Healthy	Ref		Ref		Ref	
	MASLD+normal PP	1.36 (1.31, 1.42)	<0.001	1.38 (1.33, 1.44)	<0.001	1.26 (1.20, 1.33)	<0.001
	MASLD+low PP-NO	1.40 (1.22, 1.61)	<0.001	1.38 (1.20, 1.58)	<0.001	1.21 (1.04, 1.41)	0.015
	MASLD+low PP-O	1.61 (1.49, 1.75)	<0.001	1.62 (1.49, 1.75)	<0.001	1.35 (1.23, 1.49)	<0.001
	MASLD+low PP	1.56 (1.45, 1.67)	<0.001	1.56 (1.45, 1.67)	<0.001	1.32 (1.21, 1.43)	<0.001

Table S38. Long-term mortality rates according to categories of MASLD and sarcopenia-related abnormalities.

		Incidence			
**Outcomes**	**Groups**	**No. participants**	**Incident cases**	**PYs**	**Incidence (10,000 PYs)**
Muscle strength	Healthy	4930	253	33435.17	75.67 (66.63, 85.59)
	MASLD+normal MS	3797	282	25428.08	110.90 (98.33, 124.63)
	MASLD+low MS	73	28	409.92	683.07 (453.89, 987.22)
	Non-obesity	19	6	110.58	542.58 (199.12, 1180.96)
	Obesity	54	22	299.33	734.97 (460.60, 1112.75)
Muscle mass	Healthy	5185	47	26526.67	17.72 (13.02, 23.56)
	MASLD+normal MM	4302	75	21459.42	34.95 (27.49, 43.81)
	MASLD+low MM	35	3	182.08	164.76 (33.98, 481.50)
	Non-obesity	33	3	169.33	177.17 (36.54, 517.75)
	Obesity	2	0	12.75	0.00 (0.00, 2893.24)

Table S39. Association between combined MASLD and sarcopenia-related abnormalities status with death.

		Model1		Model2		Model3	
**Outcomes**	**Groups**	**HR (95% CI)**	**P value**	**HR (95% CI)**	**P value**	**HR (95% CI)**	**P value**
Muscle strength	Healthy						
	MASLD+normal MS	1.16 (0.97, 1.39)	0.102	1.18 (0.99, 1.41)	0.067	0.74 (0.54, 1.02)	0.066
	MASLD+low MS	2.20 (1.47, 3.30)	<0.001	1.98 (1.29, 3.02)	0.002	1.86 (1.08, 3.19)	0.024
	Non-obesity	1.80 (0.80, 4.06)	0.158	1.77 (0.78, 3.99)	0.171	1.43 (0.56, 3.65)	0.45
	Obesity	2.35 (1.50, 3.70)	<0.001	2.06 (1.27, 3.33)	0.003	2.10 (1.14, 3.87)	0.018
Muscle mass	Healthy						
	MASLD+normal MM	1.44 (0.98, 2.13)	0.065	1.46 (0.99, 2.17)	0.058	0.58 (0.31, 1.09)	0.089
	MASLD+low MM	5.17 (1.58, 16.91)	0.007	5.20 (1.59, 17.03)	0.006	1.51 (0.18, 12.50)	0.705
	Non-obesity	5.38 (1.64, 17.59)	0.005	5.41 (1.65, 17.71)	0.005	1.51 (0.18, 12.50)	0.705
	Obesity	—	—	—	—	—	—

Model 1 was adjusted for age, sex, race/ethnicity, education level, and poverty-income ratio. Model 2 further adjusted for the fibrosis-4 (FIB-4) score. Model 3 additionally adjusted for alanine aminotransferase (ALT), albumin, alkaline phosphatase (ALP), total cholesterol, gamma-glutamyl transferase (GGT), glycated hemoglobin (HbA1c), platelet count, high-density lipoprotein cholesterol (HDL-C), low-density lipoprotein cholesterol (LDL-C), and triglycerides.
